# *Streptococcus pneumoniae* serotype 33H: a novel serotype with frameshift mutations in the acetyltransferase gene *wciG*

**DOI:** 10.1186/s41479-025-00162-2

**Published:** 2025-03-25

**Authors:** Sam Manna, Belinda D. Ortika, Joel P. Werren, Casey L. Pell, Ilche Gjuroski, Stephanie W. Lo, Jason Hinds, Odgerel Tundev, Eileen M. Dunne, Bradford D. Gessner, Fiona M. Russell, E. Kim Mulholland, Tuya Mungun, Claire von Mollendorf, Stephen D. Bentley, Markus Hilty, Neil Ravenscroft, Catherine Satzke

**Affiliations:** 1 Infection, Immunity and Global Health, Murdoch Children’s Research Institute, Melbourne, Australia; 2 Department of Paediatrics, The University of Melbourne, Melbourne, Australia; 3 Department of Microbiology and Immunology, The University of Melbourne at the Peter Doherty Institute for Infection and Immunity, Melbourne, Australia; 4 Institute for Infectious Diseases, University of Bern, Bern, Switzerland; 5 Department of Chemistry and Biochemistry, University of Bern, Bern, Switzerland; 6 Milner Center for Evolution, Life Sciences Department, University of Bath, Bath, United Kingdom; 7 Parasites and Microbes, Wellcome Sanger Institute, Hinxton, United Kingdom; 8 Institute for Infection and Immunity, University of London, St. George’s, United Kingdom; 9 BUGS Bioscience, London Bioscience Innovation Centre, London, United Kingdom; 10 National Center for Communicable Diseases, Ministry of Health, Ulaanbaatar, Mongolia; 11 Pfizer Vaccines, Collegeville, Pennsylvania United States of America; 12 Department of Infectious Disease Epidemiology, London School of Hygiene & Tropical Medicine, London, United Kingdom; 13 Department of Chemistry, University of Cape Town, Rondebosch, South Africa

**Keywords:** *Streptococcus pneumoniae*, Capsule, Serotypes, vaccines

## Abstract

**Background:**

*Streptococcus pneumoniae* (the pneumococcus) is a leading cause of community-acquired pneumonia. Pneumococci are categorised into serotypes, based on the type of capsular polysaccharide produced, which has important implications for virulence, vaccine impact and global surveillance. Recently, we identified a novel serotype, which we named 33G, that is comprised of an O-acetylated hexasaccharide repeat unit. In this study, we report and describe variants of 33G, designated 33G-like, which we isolated from the nasopharynx of two adults hospitalised with pneumonia in Mongolia.

**Methods:**

Serological comparison of 33G and 33G-like pneumococci were conducted by Quellung serotyping. Genetic analysis of the capsular polysaccharide loci was performed using whole genome sequencing. Polysaccharide composition was determined using ^1^H nuclear magnetic resonance.

**Results:**

By Quellung serotyping, 33G pneumococci type as both 10B and 33B whereas 33G-like pneumococci type as both 10B and 33F. Genomic analysis of the capsular polysaccharide locus revealed 33G-like loci are identical to 33G, except for frameshift mutations in the *wciG* gene which encodes an acetyltransferase responsible for the O-acetylation of beta-galactofuranose (β-Gal*f*) in the capsular polysaccharide repeat unit. We constructed an artificial 33G-like by deleting *wciG* in a 33G strain and confirmed this gene was responsible for the serological differences between 33G and 33G-like pneumococci. Lastly, ^1^H nuclear magnetic resonance confirmed the O-acetylation present in the 33G polysaccharide is absent in the 33G-like polysaccharide.

**Conclusions:**

Here, we have provided serological, genetic and biochemical evidence that the 33G-like capsule differs to 33G and all other pneumococcal serotypes, meeting the requirements to be designated as a new serotype, which we have named 33H.

**Supplementary Information:**

The online version contains supplementary material available at 10.1186/s41479-025-00162-2.

## Background

*Streptococcus pneumoniae* (the pneumococcus) is a bacterial pathogen of global importance and a major causative agent of pneumonia as well as other diseases including sepsis and meningitis [1]. Surrounding the bacterial cell is a polysaccharide capsule, with over 100 biochemically distinct capsule types (‘serotypes’) reported to date [2, 3]. Pneumococcal serotype plays an important role in virulence and is the basis for current vaccine formulations.

Novel serotypes or molecular variants of existing serotypes have the potential to reduce the accuracy of serotyping data used to inform decisions around vaccine introduction or maintenance [4–6]. Furthermore, novel serotypes may be more virulent or transmissible. For example, discovered in the last 15 years, serotypes 11E and 35D are both reported to be more invasive than related members of serogroups 11 and 35 [7–9]. Therefore, it is essential we monitor and investigate these serotypes as they emerge so that prevention strategies can be adapted.

Recently, we reported a novel serotype of pneumococcus detected in Mongolia [2]. The 33G capsular polysaccharide (*cps*) locus is likely derived from a series of recombination events with several other serotypes and streptococcal species [2]. To date, over 25 isolates of this serotype have been identified across three countries [2, 10, 11]. The 33G capsular repeat unit consists of an O-acetylated hexasaccharide with no side chains. When typed by serological methods (Quellung reaction or latex agglutination), 33G pneumococci react with typing sera such that the interpretation yields two serotyping results (10B and 33B) [2].

During our investigation of serotype variants across the Asia-Pacific region, we isolated novel variants (33G-like). The 33G-like isolates exhibit similar but distinct properties to serotype 33G, and also differ to all other known serotypes. Here, we determined the serological, genetic and biochemical basis of these differences and provide evidence that 33G-like meets the criteria to be designated a new serotype, which we have named 33H.

## Methods

### Human ethics approval

See declarations section.

### Pneumococcal identification and serotyping

Nasopharyngeal swabs from the adult pneumonia surveillance program [12] were collected, stored and tested in accordance with the World Health Organization recommendations [13]. Screening of swabs for the presence of pneumococci, as well as culture and serotyping by DNA microarray (Senti-SP v 1.5, BUGS Bioscience) and Quellung was conducted as described previously [14, 15].

### Whole genome sequencing

Bacterial DNA was extracted from either pure isolates or plate sweeps cultured on horse blood agar or horse blood agar containing 5 µg/ml gentamicin (Thermo Fisher Scientific), respectively, using the QIAamp 96 DNA QIAcube HT kit [14]. For isolates, whole genome sequencing was conducted with the Illumina DNA Prep kit (Illumina) on the NovaSeq 6000 platform (2 × 150 bp paired end reads). Genome assembly and annotation was conducted as described previously [2]. For plate sweeps, deep sequencing was conducted as described above except that samples were sequenced to a greater depth (14 million reads). Verification of *wciG* mutations in the deep sequenced samples was conducted using snippy 4.6.0 (https://github.com/tseemann/snippy) against the 33G *cps* locus using default parameters. The 33H *cps* loci have been deposited in Genbank (accession numbers PQ281427 and PQ281428).

### PCR and amplicon sequencing

PCR targeting the *wciG* gene was conducted using the KAPA HiFi HotStart ReadyMix (KAPA Biosystems). PCR reactions contained 1x KAPA HiFi HotStart ReadyMix, 1 µl of DNA template and 300 nM of primers flanking the *wciG* gene (for primer sequences, see Supplementary Table 1). PCRs were run under the following cycling conditions: 95 °C for 3 min, 35 cycles of 98 °C for 20 s, 60 °C for 15 s and 72 °C for 2 min, followed by a final extension at 72 °C for 3 min. Following cleanup using the Wizard SV gel and PCR cleanup system (Promega), the amplicons were sent to the Australian Genome Resource Facility for Sanger sequencing using the primers listed in Supplementary Table 1. Sequences were then assembled using cap3 10.2011 [16] and aligned to the reference *wciG* gene from 33G by Clustal Omega (https://www.ebi.ac.uk/jdispatcher/msa/clustalo).

### Construction of the ∆*wciG* mutant

The ∆*wciG* mutant was constructed in PMP1612 (a known 33G strain characterised previously [2]) using an overlapping PCR product consisting of a Janus cassette (containing a kanamycin resistance marker) [17], flanked by ~ 3 kb of pneumococcal DNA sequences that are upstream and downstream of the *wciG* gene (5’ and 3’ homology arms, respectively). To construct the overlapping PCR product, each product was first amplified individually using the KAPA HiFi HotStart ReadyMix (KAPA Biosystems). PCR reactions contained 1x KAPA HiFi HotStart ReadyMix, 1 µl of DNA template and 300 nM of primers (see Supplementary Table 1) and was run under the following cycling conditions; initial denaturation of 95 °C for 3 min, 35 cycles of 98 °C for 20 s, 65 °C for 15 s and 72 °C for 3 min followed by a final extension at 72 °C for 6 min. Overlapping PCR combining the amplicons was then conducted with the reaction containing 1x KAPA HiFi HotStart ReadyMix and 20ng of each of the three individual PCR products. This was run at initial denaturation of 95 °C for 3 min, 15 cycles of 98 °C for 20 s, 65 °C for 15 s and 72 °C for 8 min followed by a final extension at 72 °C for 16 min at which point 300 nM of the forward 5’ homology arm primer and the reverse 3’ homology arm primer were added. The reaction was then run under the following cycling conditions; initial denaturation of 95 °C for 3 min, 20 cycles of 98 °C for 20 s, 70 °C for 15 s and 72 °C for 8 min.

For transformation, PMP1612 was incubated in 500 µl CTM (1% [w/v] Casamino Acids, 0.5% [w/v] tryptone, 0.5% [w/v] NaCl, 1% [w/v] yeast extract, 16 µM K_2_HPO_4_, 0.2% [w/v] glucose, 150 µg/ml glutamine) and 55 ng CSP-2 competence stimulating peptide for 10 min at 37 °C with 5% CO_2_. Following incubation, 10 µl of overlapping PCR product was added and the culture was incubated at 32 °C for 30 min. The culture was then moved to 37 °C with 5% CO_2_ and incubated for 4 h before being cultured on horse blood agar plates supplemented with 500 µg/ml of kanamycin.

### NMR analysis

Extraction, purification and ^1^H NMR analysis of 33G and 33G-like polysaccharides was performed as described previously [2]. Briefly, 1D ^1^H NMR of polysaccharides was recorded at 500 MHz at 25 °C using a Bruker Neo 500 MHz NMR spectrometer. Signals from low molecular weight species (e.g. solvent, amino acids and residuals from sample processing) were removed from the proton spectra using the DOSY Bruker programme ledbpg2s1d.

## Results

### Identification of 33G-like pneumococci in Mongolia

In Mongolia, we conducted pneumococcal carriage surveillance in adults hospitalised with pneumonia from 2019 to 2022. The participants in this program did not receive the 13-valent pneumococcal conjugate vaccine (PCV13), which was introduced into the infant immunisation schedule in 2016 [12]. Nasopharyngeal swabs were collected and screened for pneumococci by qPCR targeting the *lytA* gene. Culture positive samples were then serotyped by DNA microarray. Interestingly, DNA microarray identified two samples containing a presumptive serotype 33G, which DNA microarray designates as ‘35A/10B-like’. However, when these pneumococci were isolated and serotyped by Quellung reaction, they did not type as 33G as was expected. When typed by Quellung, 33G pneumococci yield two serotype results; 10B and 33B (including positive reactions with factor sera 10b, 10d and 33f) [2]. Instead, these pneumococcal isolates (PMP1615 and PMP1623, Table 1) typed as 10B and 33F (including positive reactions with factor sera 10b, 10d and 33b, Table 2). We designated these variants ‘33G-like’. To date, no other 33G-likes were identified in our other studies including in over 9,500 nasopharyngeal swabs from healthy children [18, 19] as well as children hospitalised with pneumonia [20] in Mongolia, nor from the over 18,000 swabs from five other countries we have tested previously [2].

**Table 1:** Pneumococcal strains used in this study

Strain	Serotype	GPSC^a^	Source/Description	Year	Reference
PMP1615	33G-like	687	Nasopharyngeal isolate from a participant (aged 30–39 years) from the Sukhbaatar district in Ulaanbaatar, Mongolia, hospitalised with pneumonia	2020	This study
PMP1623	33G-like	230	Nasopharyngeal isolate from a participant (aged 40–49 years) from the Sukhbaatar district in Ulaanbaatar, Mongolia, hospitalised with pneumonia	2020	This study
PMP1612	33G	687	Nasopharyngeal isolate from a participant (aged 30–39 years) from the Chingeltei district in Ulaanbaatar, Mongolia, hospitalised with pneumonia. Used as a control 33G strain for this study	2020	[2]
*∆wciG*	Artificial 33G-like	687	*wciG* mutant constructed in PMP1612 background	N/A	This study

^**a**^ Global Pneumococcal Sequencing Cluster [21]

**Table 2:** Quellung reaction profile of representative 33G and 33G-like Pneumococci with ‘+’ and ‘-’ denoting a positive and negative reaction with typing Sera from SSI diagnostica, respectively

	Sera	33G	33G-like
**First pool**	A	-	-
	B	-	-
	C	-	-
	D	-	-
	E	+	+
	F	-	-
	G	-	-
	H	-	-
	I	-	-
**Second pool**	P	-	-
	Q	-	-
	R	-	-
	S	+	+
	T	+	+
**Group**	10	+	+
	33	+	+
**Group 10 factors**	10b	+	+
	10d	+ (weak)^a^	+ (weak)^a^
	10f	-	-
**Group 33 factors**	33b	-	+
	33e	-	-
	33f	+	-
	6a	-	-
	20b	-	-
**Serotype result**		10B and 33B	10B and 33F

^a^ Reaction is weak with one 1 µl loopful of 10d factor sera. A stronger reaction was obtained using two 1 µl loopfuls of sera

### Genetic analysis of the 33G-like *cps* locus

We next conducted whole genome sequencing on the two 33G-like isolates. Each isolate belonged to a different multi-locus sequence type and global pneumococcal sequencing cluster as inferred from pubMLST [22, 23] and popPUNK [24], respectively (ST6318 and GPSC687 for PMP1615, ST2754 and GPSC230 for PMP1623). The 33G pneumococci identified in Mongolia previously belong to these same lineages (*n* = 3 for ST2754 and GPSC230, *n* = 17 for ST6318 and GPSC687) [2].

**Fig. 1:**
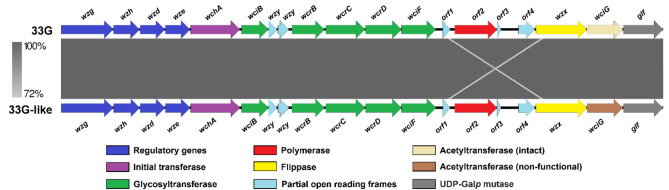
Schematic comparison of the 33G and 33G-like *cps* loci. Shading indicates DNA sequence identity (%). The light grey cross indicates a related sequence between *orf1* and *wzx*, which may be a remnant of the recombination event that gave rise to the 33G *cps* locus. Image was created using Easyfig version 2.2.5 [28]. For full DNA sequence alignments, see Supplementary Figs. 1 and 2

We next compared the *cps* locus from each 33G-like isolate to the 33G *cps* locus. The 33G-like and 33G *cps* loci were identical except that both 33G-like isolates had frameshift mutations in the *wciG* gene (Supplementary Figs. 1 and 2, Fig. 1). This gene encodes an acetyltransferase responsible for the O-acetylation of the galactofuranose (Gal*f*) residue in the polysaccharide repeat unit [25–27]. PMP1615 has a single nucleotide deletion in *wciG*, whereas PMP1623 has two frameshift mutations (a single nucleotide insertion and an 11 bp deletion in *wciG*), (Table 3). These mutations are predicted to result in the generation of premature stop codons and therefore *wciG* is unlikely to encode a functional acetyltransferase in these 33G-like isolates (Supplementary Figs. 3 and 4). All mutations were verified by traditional Polymerase Chain Reaction (PCR) amplification and Sanger sequencing of *wciG* from these isolates (Supplementary Fig. 5).

The same mutations were also detected when we re-cultured the nasopharyngeal samples and conducted traditional PCR targeting *wciG* using DNA extracted from a sweep of bacterial growth from the culture plate (Supplementary Fig. 6). We also conducted deep sequencing on the DNA extracted from the plate sweep. For the sample from which PMP1615 is derived, 99.3% (278/280) of reads covering position 313 of *wciG* matched the mutant allele observed in the isolate. Similarly, for the sample from which PMP1623 was isolated, 100% of reads covering positions 163 (298/298) and 766 (141/141) of *wciG* matched the mutant allele in the isolate.

**Table 3:** Frameshift mutations in *wciG* of 33G-like Pneumococci relative to *wciG* in 33G

Isolate	Gene size (bp)^a^	Mutation	Nucleotide position^b^	Amino acid change	Amino acid length^c^	Predicted consequence
PMP1615	998	Single nucleotide deletion (A)	313	Amino acid 105 change from Ile to Leu	105	Frameshift generating premature stop codon at codon 106
PMP1623	989	Single nucleotide insertion (T)	163	Amino acid 55 change from Met to Tyr	95	Frameshift generating premature stop codon at codon 96
		Deletion of 11 bp (ATTTTCTCACT)^d^	766^d^			

^a^ Size of in frame gene in 33G = 999 bp. ^b^ Nucleotide positions are inferred from the snippy 4.6.0 tool (https://github.com/tseemann/snippy) and are approximate because all mutations are located in homopolymeric/ambiguous regions and therefore the exact position cannot be ascertained. This also applies to all DNA alignments in supplementary data. ^c^ full length of WciG in 33G = 332 amino acids. ^d^ As the 11 bp deletion occurs within a 14 bp region flanked by ‘ACT’ on either end, it is plausible the deletion is ACTATTTTCTC starting at position 763

To verify that the mutations in *wciG* were responsible for the serological differences observed in the Quellung reaction, we deleted *wciG* in PMP1612 (a 33G isolate) to create an artificial 33G-like strain (Supplementary Figs. 7 and 8). Consistent with the 33G-like pneumococci, Δ*wciG* typed as both 10B and 33F by Quellung whereas its parent 33G strain (PMP1612) typed as both 10B and 33B, which is consistent with the reaction profiles shown in Table 2. These data confirm the serological differences between 33G and 33G-like pneumococci are attributable to *wciG.*

### Elucidation of 33G-like polysaccharide repeat unit structure

**Fig. 2:**
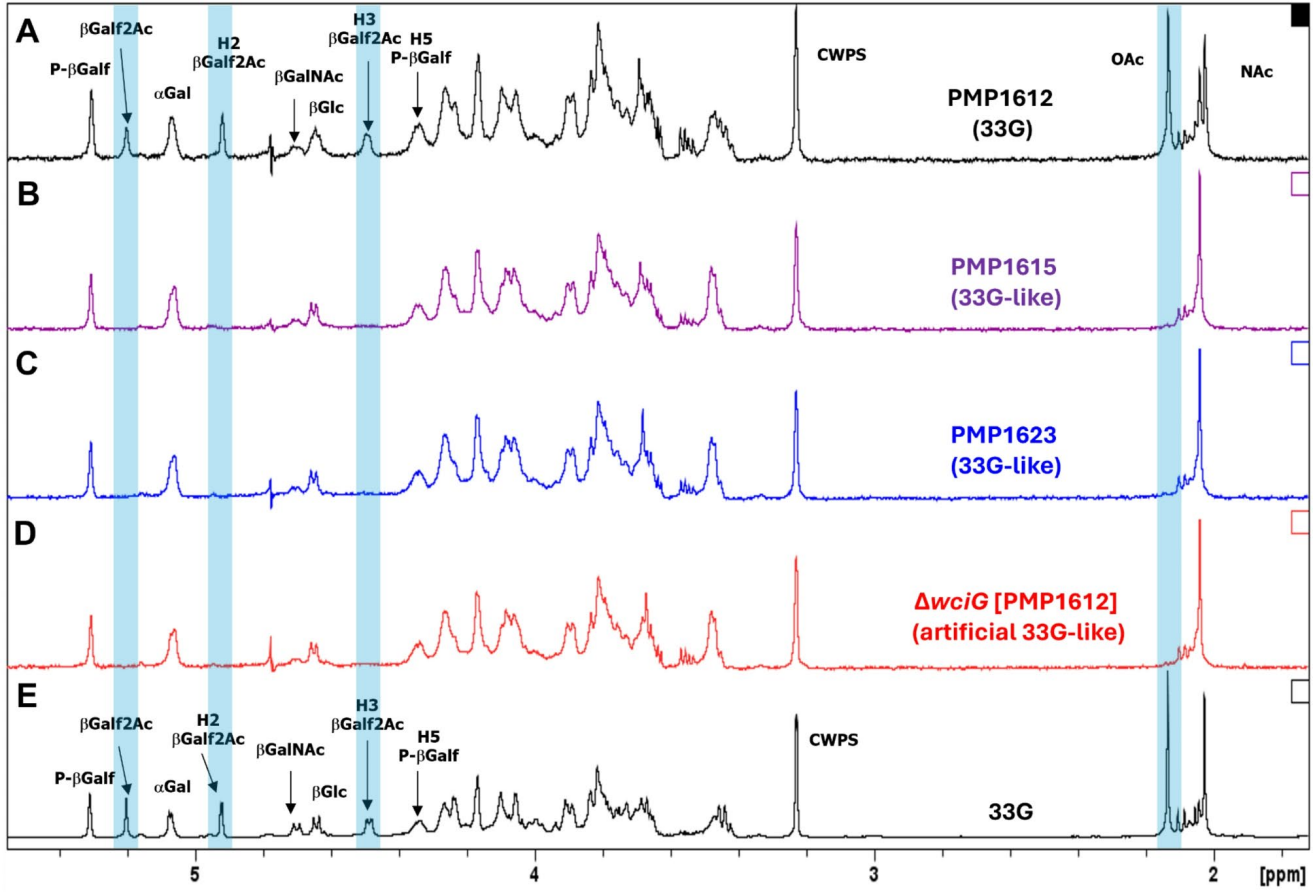
^1^H diffusion-ordered spectroscopy (DOSY) NMR spectra of 33G (**A**), 33G-like (**B**-**C**) and artificial 33G-like (**D**) pneumococcal isolates identified in this study compared with the 33G polysaccharide spectrum reported previously (**E**) [2] at 500 MHz and 25 °C. CWPS = cell wall polysaccharide. Highlighted areas denote the location of acetylation peaks

**Fig. 3:**
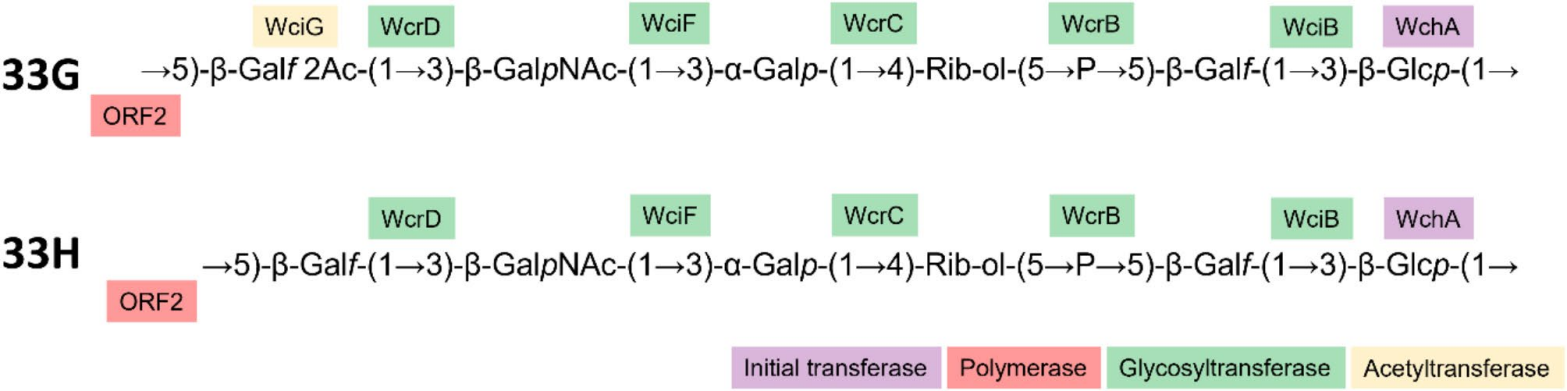
Polysaccharide repeat unit structure of 33G [2] and the new serotype 33H (33G-like) identified in this study. Enzymatic assignments have been designated based on the literature [29]

To verify the lack of O-acetylated Gal*f* in the 33G-like capsule, the polysaccharide from both 33G-like pneumococcal isolates (PMP1615 and PMP1623), a 33G isolate (PMP1612) and the artificial 33G-like (∆*wciG* [PMP1612]) strain were purified and analysed by ^1^H nuclear magnetic resonance (NMR). PMP1612 polysaccharide (serotype 33G) exhibited the same NMR spectra as reported for this serotype previously [2], including an O-acetyl signal at 2.14 ppm and the diagnostic signal for the presence of O-acetylated Gal*f* (β-Gal*f* 2Ac) at 5.20, 4.93 and 4.48 ppm for H1, H2 and H3, respectively (Fig. 2). In contrast, all four of these acetylation signals were absent in the 33G-like isolates and in the artificial *wciG* mutant strain (Fig. 2). This biochemical analysis, taken together with the genetic and serological differences to 33G and other previously described serotypes, confirm that 33G-like is a novel pneumococcal serotype, which we hereby name ‘33H’ (Fig. 3).

## Discussion

Previously, we characterised isolates from Mongolia that expressed a novel capsule and were therefore designated a new serotype, which we named 33G [2]. Now, we describe two new isolates in Mongolia that we designated 33G-like. The serological properties of the 33G-like pneumococci differed to the traditional 33G pneumococci as indicated by different reactions to the group 33 factor sera. Genetic analysis confirmed that in contrast with 33G, 33G-like pneumococci have frameshift mutations in *wciG.* The *wciG* gene in the *cps* locus encodes an acetyltransferase responsible for the O-acetylation of Gal*f* [26, 27]. Biochemical analysis confirmed the absence of acetylated Gal*f* in the 33G-like capsule repeat unit. Taken together, these data satisfy the criteria for 33G-like to be designated as a novel serotype, which we have named 33H.

Novel pneumococcal serotypes resulting from small mutations in genes within the *cps* locus have been described previously. The most recent example is serotype 33E, which has a *cps* locus and capsule structure identical to 33F, except for a frameshift mutation in the *wciE* glycosyltransferase gene resulting in the lack of the α-Gal*p* branch [26]. Serotypes 11E, 33F and 9A have mutations in the *wcjE* gene, which encodes an acetyltransferase, but otherwise have identical *cps* loci and capsule structures to 11A, 33A and 9V, respectively [30–32]. Serotype 15C is identical to 15B, except for mutations in the *wciZ* acetyltransferase gene [33]. The closest analogy to 33G and 33H may be serotypes 35B and 35D, where 35D capsules lack O-acetylation due to mutations in *wciG* compared with 35B [34]. Interestingly, the *wciG* genes from serotypes 35B and 33G are over 85% identical (Supplementary Fig. 9) and the same two single nucleotide mutations observed in *wciG* in our 33H isolates have also been reported in the *wciG* gene of 35D isolates [7]. Both mutations occur in homopolymeric regions, which are susceptible to slipped-strand mispairing leading to the insertion or deletion of a single nucleotide [4, 33, 35].

Acetyl groups on capsular polysaccharides are immunodominant epitopes and therefore are readily recognised by the immune system [36, 37]. It has therefore been proposed that there may be selective pressure on O-acetylated serotypes to lose this modification. The loss of acetylation is associated with increased immune evasion [7, 38]. For 11A/11E and 35B/35D, there is a disproportionate distribution of these serotypes isolated in carriage versus invasive pneumococcal disease, with the acetyltransferase mutant serotypes more commonly associated with invasive pneumococcal disease and acetyltransferase proficient serotypes more commonly identified in carriage [7–9]. These observations have led to a within-host microevolutionary model where it is proposed that the acetyltransferase proficient serotypes 11A and 35B show greater fitness in carriage and transmission but reduced fitness during invasive disease. In contrast, acetyltransferase mutant serotypes 11E and 35D would be more fit in a disease state but are likely an evolutionary dead-end because they are less likely to be transmitted from sites such as the blood [8]. Interestingly, this may not be the case for 33G/33H since the 33H isolates described in this study (PMP1615 and PMP1623) were isolated from the nasopharynx of pneumonia patients. Additionally, deep sequencing of plate sweeps of bacteria cultured directly from the nasopharyngeal swab only detected 33H with over 99% of sequence reads matching the mutated *wciG* allele (33H). Although we cannot exclude within-host evolution, the deep sequencing results suggest it is more likely that these pneumococci were already 33H at the time of acquisition. Future studies should focus on the association between acetylation status and invasiveness for 33G/33H.

The accuracy of methods used for pneumococcal serotyping is essential for generating reliable data for disease surveillance and/or measuring vaccine impact. Unfortunately, most molecular approaches for serotyping are currently unable to distinguish 33H from 33G. This is because most of the *cps* loci are identical, except for the mutations in the *wciG* gene, of which the site/type can vary by strain. However, distinguishing 33G and 33H with a molecular approach could be undertaken using PCR amplification and sequencing of the *wciG* gene, or by whole genome sequencing and manual interrogation of the *cps* locus for *wciG* mutations. Additionally, it may be possible to create a custom database in whole genome sequencing serotyping tools such as PneumoCaT or SeroBA, where a screen for *wciG* could be incorporated to determine if the gene is in frame, similar to screens already conducted for other serotypes that contain frameshifted capsule genes [39, 40].

Our study has identified 33H in nasopharyngeal swabs in patients hospitalised with pneumonia in Mongolia. Interestingly, one of the putative 33G invasive isolates from South Africa (GPS_ZA_887, ENA sample accession SAMEA2553998, isolated from the blood of a 5 month old child) identified in the Global Pneumococcal Sequencing project [21] contains a single T insertion in *wciG* at nucleotide position 558, resulting in a frameshift and formation of a premature stop codon, suggesting this isolate is serotype 33H. Therefore, evidence indicates that 33H is present in at least two countries and can cause invasive disease. Future studies should focus on understanding the epidemiology of 33H in other countries. Lastly, given 33H reacts with the same pool, group and factor sera as 33F, it is evident that there is cross-reactivity between these serotypes. Importantly, serotype 33F is a component of recent vaccine formulations including PCV15 and PCV20 [41]. Thus, future studies should explore the potential of these vaccines to provide cross-protection against 33H.

## Conclusion

Here we provide genetic, serological and biochemical evidence that the 33G-like capsular polysaccharide differs to the related serotype 33G, as well as all other known pneumococcal serotypes. Taken together, these data satisfy the criteria to designate 33G-like as a new capsular serotype, hereby named 33H. Future work investigating 33H including its geographical distribution and potential for enhanced immune evasion over 33G, will shed light on the evolution of this serotype as well as improve our understanding of drivers mediating the emergence of new serotypes.

## Electronic supplementary material

Below is the link to the electronic supplementary material.

Supplementary Material 1

## Data Availability

The 33H cps loci have been deposited in Genbank (accession numbers PQ281427 and PQ281428). All other data generated during this study are included in the published article.

## Abbreviations

cps: Capsular polysaccharide locus
GPSC: Global pneumococcal sequencing cluster
MLST: Multi-locus sequence type
Gal*f*: Galactofuranose
PCR: Polymerase chain reaction
NMR: Nuclear magnetic resonance
Gal*f* 2Ac: O-acetylated galactofuranose
DOSY: Diffusion-ordered spectroscopy
CWPS: Cell wall polysaccharide
