# Supplementary material for: Streptococcus pneumoniae serotype 33H: a novel serotype with frameshift mutations in the acetyltransferase gene wciG
Source: Pneumonia (Nathan). 2025 Mar 25;17:7. doi: 10.1186/s41479-025-00162-2 (PMC11934437; doi:10.1186/s41479-025-00162-2)
Supplement: Supplementary file 1 — Supplementary Material 1 [file 41479_2025_162_MOESM1_ESM.docx]

**Supplementary data: Manna *et al.***

***Streptococcus pneumoniae* serotype 33H: a novel serotype with frameshift mutations in the acetyltransferase gene *wciG***

Supplementary Table 1: List of primers used in this study.

| **Purpose** | **Sequence (5’ to 3’)** |
| --- | --- |
| PCR amplification of *wciG* | **Forward:**  TTCTAGCATTTGCAGCGCTTGGTGG  **Reverse:**  GAGACCAGCACCAACAACAAGATAATCG |
| Sanger sequencing of *wciG* PCR product | **Forward:**  ACTATCTTTTAGGTGGTTATATAGCG  ATTTATATTATTTAGGTACTTACTCTATTCCCC  **Reverse:**  TTTGAATTTAGAAATTGTCTCACAATTGGC  TTATTAGTAAAAGTGGTAAGCGTAGG |
| Overlapping PCR for construction of Δ*wciG* | **5’ homology arm forward:**  CGCTTCAGCAAGTATTCGATATGATGATTATGTAGCAGG  **5’ homology arm reverse^a,b^:**  CATTATCCATTAAAAATCAAACGGGCAAGCACTTTTAGTAAATCTAGG  **Janus cassette forward^a,b^:**  CCTAGATTTACTAAAAGTGCTTGCCCGTTTGATTTTTAATGGATAATG  **Janus cassette reverse^b,c^:**  CCATACCTTCATAATATAAGTGTGTTTTCTCTTTATCCCCTTTCC  **3’ homology arm forward^b,c^:**  GGAAAGGGGATAAAGAGAAAACACACTTATATTATGAAGGTATGG  **3’ homology arm reverse:**  CAATAGCCTGACGGAAATCCTTGTTTAAGAGAGCC |

^a^ Primers are reverse complement of one another for overlapping PCR.

^b^ Underlined sequence corresponds to Janus cassette sequence primers adapted from Sung et al. (1).

^c^ Primers are reverse complement of one another for overlapping PCR.

PMP1612_cps ATGAGTAGACGTTTTAAAAAATCAGGTTCACAGAAAGTGAAGCGAAGTGTTAATATCGTT 60

PMP1615_cps ATGAGTAGACGTTTTAAAAAATCAGGTTCACAGAAAGTGAAGCGAAGTGTTAATATCGTT 60

************************************************************

PMP1612_cps TTGCTGACTATTTATTTATTGTTAGTTGGTTTTTTATTGTTCTTAATCTTTAAGTACAAT 120

PMP1615_cps TTGCTGACTATTTATTTATTGTTAGTTGGTTTTTTATTGTTCTTAATCTTTAAGTACAAT 120

************************************************************

PMP1612_cps ATCCTTGCTTTTAGATATCTTAACCTAGTGGTAACTGCGTTAGTCCTACTAGTTGCCTTG 180

PMP1615_cps ATCCTTGCTTTTAGATATCTTAACCTAGTGGTAACTGCGTTAGTCCTACTAGTTGCCTTG 180

************************************************************

PMP1612_cps GTAGGGCTACTCTTGGTTATCTATAAAAAAGCTGAAAAATTTACTATTTTTCTGTTGCTG 240

PMP1615_cps GTAGGGCTACTCTTGGTTATCTATAAAAAAGCTGAAAAATTTACTATTTTTCTGTTGCTG 240

************************************************************

PMP1612_cps TTCTCTATCCTTGTCAGCTCTGTGTCGCTCTTTGCAGTACAGCAGTTTGTTGGACTGACC 300

PMP1615_cps TTCTCTATCCTTGTCAGCTCTGTGTCGCTCTTTGCAGTACAGCAGTTTGTTGGACTGACC 300

************************************************************

PMP1612_cps AATCGTTTAAATGCGACTTCTAATTACTCAGAATATTCAATCAGTGTCGCTGTTTTAGCA 360

PMP1615_cps AATCGTTTAAATGCGACTTCTAATTACTCAGAATATTCAATCAGTGTCGCTGTTTTAGCA 360

************************************************************

PMP1612_cps GATAGTGATATCGAAAATGTTACGCAACTGACGAGTGTGACAGCACCGACTGGGACTGAT 420

PMP1615_cps GATAGTGATATCGAAAATGTTACGCAACTGACGAGTGTGACAGCACCGACTGGGACTGAT 420

************************************************************

PMP1612_cps AATGAAAATATTCAAAAACTACTAGCTGATATCAAGTCAAGTCAGAATACCGATTTGACG 480

PMP1615_cps AATGAAAATATTCAAAAACTACTAGCTGATATCAAGTCAAGTCAGAATACCGATTTGACG 480

************************************************************

PMP1612_cps GTCAACCAGAGTTCGTCTTACTTGGCAGCTTACAAGAGTTTGATTGCAGGGGAGACTAAG 540

PMP1615_cps GTCAACCAGAGTTCGTCTTACTTGGCAGCTTACAAGAGTTTGATTGCAGGGGAGACTAAG 540

************************************************************

PMP1612_cps GCCATTGTCCTAAATAGTGTCTTTGAAAATATCATCGAGTCAGAGTATCCAGACTACGCA 600

PMP1615_cps GCCATTGTCCTAAATAGTGTCTTTGAAAATATCATCGAGTCAGAGTATCCAGACTACGCA 600

************************************************************

PMP1612_cps TCGAAGATAAAAAAGATTTATACCAAGGGATTCACTAAAAAAGTAGAAGCTCCTAAGACG 660

PMP1615_cps TCGAAGATAAAAAAGATTTATACCAAGGGATTCACTAAAAAAGTAGAAGCTCCTAAGACG 660

************************************************************

PMP1612_cps TCTAAGAATCAGTCTTTCAATATCTATGTTAGTGGAATTGACACCTATGGCCCTATTAGT 720

PMP1615_cps TCTAAGAATCAGTCTTTCAATATCTATGTTAGTGGAATTGACACCTATGGCCCTATTAGT 720

************************************************************

PMP1612_cps TCGGTGTCGCGATCAGATGTCAATATCCTGATGACTGTCAATCGAGATACCAAGAAAATC 780

PMP1615_cps TCGGTGTCGCGATCAGATGTCAATATCCTGATGACTGTCAATCGAGATACCAAGAAAATC 780

************************************************************

PMP1612_cps CTCTTGACCACAACGCCACGTGATGCCTATGTACCAATCGCAGATGGTGGAAATAATCAA 840

PMP1615_cps CTCTTGACCACAACGCCACGTGATGCCTATGTACCAATCGCAGATGGTGGAAATAATCAA 840

************************************************************

PMP1612_cps AAAGATAAATTAACCCATGCAGGCATTTATGGAGTTGATTCGTCCATTCACACCTTAGAA 900

PMP1615_cps AAAGATAAATTAACCCATGCAGGCATTTATGGAGTTGATTCGTCCATTCACACCTTAGAA 900

************************************************************

PMP1612_cps AATCTCTATGGAGTGGATATCAATTACTATGTGCGATTGAACTTCACTTCGTTTTTGAAA 960

PMP1615_cps AATCTCTATGGAGTGGATATCAATTACTATGTGCGATTGAACTTCACTTCGTTTTTGAAA 960

************************************************************

PMP1612_cps TTGATTGATTTGTTGGGTGGAATTGATGTTTATAATGATCAAGAATTTACTGCCCATACG 1020

PMP1615_cps TTGATTGATTTGTTGGGTGGAATTGATGTTTATAATGATCAAGAATTTACTGCCCATACG 1020

************************************************************

PMP1612_cps AATGGAAAGTATTACCCTGCAGGCAATGTTCATCTTGATTCAGAACAGGCTCTCGGTTTT 1080

PMP1615_cps AATGGAAAGTATTACCCTGCAGGCAATGTTCATCTTGATTCAGAACAGGCTCTCGGTTTT 1080

************************************************************

PMP1612_cps GTTCGTGAGCGCTACTCCCTAGCAGATGGCGATCGTGACCGCGGGCGCAATCAACAAAAG 1140

PMP1615_cps GTTCGTGAGCGCTACTCCCTAGCAGATGGCGATCGTGACCGCGGGCGCAATCAACAAAAG 1140

************************************************************

PMP1612_cps GTGATTGTGGCTATCCTTCAAAAATTAACGTCAACCGAAGCACTGAAAAATTATAGTACG 1200

PMP1615_cps GTGATTGTGGCTATCCTTCAAAAATTAACGTCAACCGAAGCACTGAAAAATTATAGTACG 1200

************************************************************

PMP1612_cps ATCATTGATAGCTTGCAAGATTCTATCCAAACAAATATGCCACTTGAGACAATGATAAAT 1260

PMP1615_cps ATCATTGATAGCTTGCAAGATTCTATCCAAACAAATATGCCACTTGAGACAATGATAAAT 1260

************************************************************

PMP1612_cps TTGGTCAATGCTCAGTTGGAAAGTGGAGGGAATTATAAAGTAAATTCTCAAGATTTAAAA 1320

PMP1615_cps TTGGTCAATGCTCAGTTGGAAAGTGGAGGGAATTATAAAGTAAATTCTCAAGATTTAAAA 1320

************************************************************

PMP1612_cps GGGACAGGTCGGATGGATCTTCCTTCTTATGCAATGCCAGACAGTAACCTCTATGTGATG 1380

PMP1615_cps GGGACAGGTCGGATGGATCTTCCTTCTTATGCAATGCCAGACAGTAACCTCTATGTGATG 1380

************************************************************

PMP1612_cps GAAATAGATGATAGTAGTTTAGCTGTAGTTAAAGCAGCTATACAGGATGTGATGGAGGGT 1440

PMP1615_cps GAAATAGATGATAGTAGTTTAGCTGTAGTTAAAGCAGCTATACAGGATGTGATGGAGGGT 1440

************************************************************

PMP1612_cps AGATGAAATGATAGACATCCATTCGCACATCGTCTTTGATGTAGATGATGGTCCCAAGTC 1500

PMP1615_cps AGATGAAATGATAGACATCCATTCGCACATCGTCTTTGATGTAGATGATGGTCCCAAGTC 1500

************************************************************

PMP1612_cps AAGGGAGGAAAGCAAGACTCTCTTGGCAGAAGCCTATAGACAGGGGGTGCGAACCATTGT 1560

PMP1615_cps AAGGGAGGAAAGCAAGACTCTCTTGGCAGAAGCCTATAGACAGGGGGTGCGAACCATTGT 1560

************************************************************

PMP1612_cps CTCTACCTCTCACCGTCGCAAGGGCATGTTTGAAACTCCGGAAGAGAAGATAGCAGAAAA 1620

PMP1615_cps CTCTACCTCTCACCGTCGCAAGGGCATGTTTGAAACTCCGGAAGAGAAGATAGCAGAAAA 1620

************************************************************

PMP1612_cps CTTTCTTCAGGTTCGGGAAATAGCTAAGGAAGTGGCGAGTGACTTGGTCATTGCTTACGG 1680

PMP1615_cps CTTTCTTCAGGTTCGGGAAATAGCTAAGGAAGTGGCGAGTGACTTGGTCATTGCTTACGG 1680

************************************************************

PMP1612_cps GGCTGAAATTTACTACACACCAGATATTCTGGATAAGCTGGAAAAAAAGCGGATTCCGAC 1740

PMP1615_cps GGCTGAAATTTACTACACACCAGATATTCTGGATAAGCTGGAAAAAAAGCGGATTCCGAC 1740

************************************************************

PMP1612_cps CCTCAATGATAGTCGTTATGCCTTGATAGAGTTTAGTGTGAACACTCCTTATCGCGATAT 1800

PMP1615_cps CCTCAATGATAGTCGTTATGCCTTGATAGAGTTTAGTGTGAACACTCCTTATCGCGATAT 1800

************************************************************

PMP1612_cps TCATAGCGCCTTGAGCAAGATCTTGATGTTGGGAATTACTCCAGTCATTGCCCACATTGA 1860

PMP1615_cps TCATAGCGCCTTGAGCAAGATCTTGATGTTGGGAATTACTCCAGTCATTGCCCACATTGA 1860

************************************************************

PMP1612_cps GCGCTATGATGCTCTTGAAAATAATGAAAAACGCGTTCGAGAACTGATCGATATGGGCTG 1920

PMP1615_cps GCGCTATGATGCTCTTGAAAATAATGAAAAACGCGTTCGAGAACTGATCGATATGGGCTG 1920

************************************************************

PMP1612_cps TTACACGCAAGTAAATAGTTCACATGTCCTCAAACCCAAACTTTTTGGCGAACGTTATAA 1980

PMP1615_cps TTACACGCAAGTAAATAGTTCACATGTCCTCAAACCCAAACTTTTTGGCGAACGTTATAA 1980

************************************************************

PMP1612_cps ATTCATGAAAAAAAGAGCTCAGTATTTTTTAGAGCAGGATTTGGTTCATGTCATTGCAAG 2040

PMP1615_cps ATTCATGAAAAAAAGAGCTCAGTATTTTTTAGAGCAGGATTTGGTTCATGTCATTGCAAG 2040

************************************************************

PMP1612_cps TGATATGCACAATCTAGACGGTAGACCTCCTCATATGGCAGAAGCATATGACCTTGTTAC 2100

PMP1615_cps TGATATGCACAATCTAGACGGTAGACCTCCTCATATGGCAGAAGCATATGACCTTGTTAC 2100

************************************************************

PMP1612_cps CCAAAAATACGGAGAAGCGAAGGCTCAGGAACTTTTTATAGACAATCCTCGAAAAATTGT 2160

PMP1615_cps CCAAAAATACGGAGAAGCGAAGGCTCAGGAACTTTTTATAGACAATCCTCGAAAAATTGT 2160

************************************************************

PMP1612_cps AATGGATCAACTAATTTAGGAGAAATGATGAAAGAACAAAACACGATAGAAATCGATGTA 2220

PMP1615_cps AATGGATCAACTAATTTAGGAGAAATGATGAAAGAACAAAACACGATAGAAATCGATGTA 2220

************************************************************

PMP1612_cps TTTCAATTAGTTAAAACCTTGTGGAAACGCAAGCTAATGATTTTAATAGTGGCACTTGTG 2280

PMP1615_cps TTTCAATTAGTTAAAACCTTGTGGAAACGCAAGCTAATGATTTTAATAGTGGCACTTGTG 2280

************************************************************

PMP1612_cps ACAGGTGCGGGGGCTTTTGCATATAGCACTTTTATTGTTAAGCCAGAATATACGAGTACC 2340

PMP1615_cps ACAGGTGCGGGGGCTTTTGCATATAGCACTTTTATTGTTAAGCCAGAATATACGAGTACC 2340

************************************************************

PMP1612_cps ACGCGAATTTACGTAGTGAATCGCAATCAAGGAGACAAGCCGGGGCTGACAAATCAGGAT 2400

PMP1615_cps ACGCGAATTTACGTAGTGAATCGCAATCAAGGAGACAAGCCGGGGCTGACAAATCAGGAT 2400

************************************************************

PMP1612_cps TTGCAGGCAGGAACTTATCTGGTAAAAGACTACCGTGAGATTATCCTTTCGCAGGATGCA 2460

PMP1615_cps TTGCAGGCAGGAACTTATCTGGTAAAAGACTACCGTGAGATTATCCTTTCGCAGGATGCA 2460

************************************************************

PMP1612_cps TTGGAAAAAGTAGCGACAAATTTGAAGTTGGATATGTCAGCAAAAACGTTAGCCAGCAAA 2520

PMP1615_cps TTGGAAAAAGTAGCGACAAATTTGAAGTTGGATATGTCAGCAAAAACGTTAGCCAGCAAA 2520

************************************************************

PMP1612_cps GTTCAAGTGGCTGTACCAGCTGACACTCGTATCGTCTCAATCTCTGTCAAGGATAAACAG 2580

PMP1615_cps GTTCAAGTGGCTGTACCAGCTGACACTCGTATCGTCTCAATCTCTGTCAAGGATAAACAG 2580

************************************************************

PMP1612_cps CCAGAGGAAGCCAGTCGTATCGCTAATTCTCTACGAGAAGTTGCTGCAGAAAAGATCGTC 2640

PMP1615_cps CCAGAGGAAGCCAGTCGTATCGCTAATTCTCTACGAGAAGTTGCTGCAGAAAAGATCGTC 2640

************************************************************

PMP1612_cps GCTGTAACGCGAGTATCTGATGTAACGACACTTGAAGAAGCGCGACCAGCTACGACTCCC 2700

PMP1615_cps GCTGTAACGCGAGTATCTGATGTAACGACACTTGAAGAAGCGCGACCAGCTACGACTCCC 2700

************************************************************

PMP1612_cps TCTTCTCCAAATGTTCGACGCAATTCCTTGTTTGGTTTTCTTGGAGGAGCAGTCGTAACA 2760

PMP1615_cps TCTTCTCCAAATGTTCGACGCAATTCCTTGTTTGGTTTTCTTGGAGGAGCAGTCGTAACA 2760

************************************************************

PMP1612_cps GTAATTGCTGTTCTTTTGATTGAGTTGCTCGACACCCGTGTGAAACGTCCTGAAGATGTT 2820

PMP1615_cps GTAATTGCTGTTCTTTTGATTGAGTTGCTCGACACCCGTGTGAAACGTCCTGAAGATGTT 2820

************************************************************

PMP1612_cps GAAGATGTACTGCAAATTCCACTTTTAGGGCTCGTTCCAGATTTGGACAAAATGAAATAG 2880

PMP1615_cps GAAGATGTACTGCAAATTCCACTTTTAGGGCTCGTTCCAGATTTGGACAAAATGAAATAG 2880

************************************************************

PMP1612_cps GAGGAAGTTATGCCAACGTTAGAAATCTCACAGGCAAAATTGGATTTTGTAAAAAAGGCA 2940

PMP1615_cps GAGGAAGTTATGCCAACGTTAGAAATCTCACAGGCAAAATTGGATTTTGTAAAAAAGGCA 2940

************************************************************

PMP1612_cps GAGGAATATTATAACGCTTTGTGCACGAACCTACAGTTAAGTGGAGATGGTTTGAAAGTA 3000

PMP1615_cps GAGGAATATTATAACGCTTTGTGCACGAACCTACAGTTAAGTGGAGATGGTTTGAAAGTA 3000

************************************************************

PMP1612_cps TTTTCTATCACTTCTGTGAAAATAGGAGAAGGAAAATCAACGACTTCTACCAATATCGCT 3060

PMP1615_cps TTTTCTATCACTTCTGTGAAAATAGGAGAAGGAAAATCAACGACTTCTACCAATATCGCT 3060

************************************************************

PMP1612_cps TGGGCTTTTGCGCGTGCAGGTTACAAAACGCTGCTGATTGATGGAGATATTCGCAATTCT 3120

PMP1615_cps TGGGCTTTTGCGCGTGCAGGTTACAAAACGCTGCTGATTGATGGAGATATTCGCAATTCT 3120

************************************************************

PMP1612_cps GTTATGTTAGGTGTCTTTAAAGCAAGGGATAAGATTACGGGCCTGACAGAATTTTTATCA 3180

PMP1615_cps GTTATGTTAGGTGTCTTTAAAGCAAGGGATAAGATTACGGGCCTGACAGAATTTTTATCA 3180

************************************************************

PMP1612_cps GGAACTACAGACCTATCACAAGGGCTTTGTGATACCAATATCGAAAATCTCTTTGTAATT 3240

PMP1615_cps GGAACTACAGACCTATCACAAGGGCTTTGTGATACCAATATCGAAAATCTCTTTGTAATT 3240

************************************************************

PMP1612_cps CAGGCTGGCTCTGTGTCACCGAATCCGACAGCTCTTCTTCAAAGTAAGAATTTCAGTACA 3300

PMP1615_cps CAGGCTGGCTCTGTGTCACCGAATCCGACAGCTCTTCTTCAAAGTAAGAATTTCAGTACA 3300

************************************************************

PMP1612_cps ATGCTTGAAACCTTGCGTAAATATTTTGACTACATCATTGTAGATACTGCTCCTGTCGGT 3360

PMP1615_cps ATGCTTGAAACCTTGCGTAAATATTTTGACTACATCATTGTAGATACTGCTCCTGTCGGT 3360

************************************************************

PMP1612_cps GTCGTGATTGATGCTGCTATTATTACGCGAAAATGCGATGCTTCTATTTTAGTGACGGAG 3420

PMP1615_cps GTCGTGATTGATGCTGCTATTATTACGCGAAAATGCGATGCTTCTATTTTAGTGACGGAG 3420

************************************************************

PMP1612_cps GCAGGTGAAATAAATCGACGGGATATTCAAAAAGCAAAAGAACAGTTAGAACACACAGGG 3480

PMP1615_cps GCAGGTGAAATAAATCGACGGGATATTCAAAAAGCAAAAGAACAGTTAGAACACACAGGG 3480

************************************************************

PMP1612_cps AAGCCGTTTTTGGGAGTTGTGTTGAATAAATTCGATACTTCAGTAGACAAATACGGTTCT 3540

PMP1615_cps AAGCCGTTTTTGGGAGTTGTGTTGAATAAATTCGATACTTCAGTAGACAAATACGGTTCT 3540

************************************************************

PMP1612_cps TATGGAAATTATGGAGATTACGGGAAAAATAAAAAATAGGTCGGGGGATAGAGATGAATG 3600

PMP1615_cps TATGGAAATTATGGAGATTACGGGAAAAATAAAAAATAGGTCGGGGGATAGAGATGAATG 3600

************************************************************

PMP1612_cps GAAAAATAGTAAAGTCTTCATTGGCTATAATCCAGAGTTTTCTTGTTATTTTATTGACTT 3660

PMP1615_cps GAAAAATAGTAAAGTCTTCATTGGCTATAATCCAGAGTTTTCTTGTTATTTTATTGACTT 3660

************************************************************

PMP1612_cps ATCTACTTAGTGCTGTGAGAGAAGTGGAGATTGTTTCAACAACAGCTATTGCACTTTATA 3720

PMP1615_cps ATCTACTTAGTGCTGTGAGAGAAGTGGAGATTGTTTCAACAACAGCTATTGCACTTTATA 3720

************************************************************

PMP1612_cps TCCTCCATTATTTTGTCTTTTATATCAGTGATTATGGACAGGATTTCTTTAAAAGGGGAT 3780

PMP1615_cps TCCTCCATTATTTTGTCTTTTATATCAGTGATTATGGACAGGATTTCTTTAAAAGGGGAT 3780

************************************************************

PMP1612_cps ATTTGATTGAACTTGTCCAGACATTGAAATATATCCTATTCTTTGCACTAGCGATTAATA 3840

PMP1615_cps ATTTGATTGAACTTGTCCAGACATTGAAATATATCCTATTCTTTGCACTAGCGATTAATA 3840

************************************************************

PMP1612_cps TTTCTAATTTTTTCTTAGAGGATCGATTTAGTATTTCCAGACGAGGCATGATTTACTTCC 3900

PMP1615_cps TTTCTAATTTTTTCTTAGAGGATCGATTTAGTATTTCCAGACGAGGCATGATTTACTTCC 3900

************************************************************

PMP1612_cps TCACATTACATGCTCTCTTAGTCTATGTGCTAAACCTATTTATCAAGTGGTATTGGAAGC 3960

PMP1615_cps TCACATTACATGCTCTCTTAGTCTATGTGCTAAACCTATTTATCAAGTGGTATTGGAAGC 3960

************************************************************

PMP1612_cps GGGCTTATCCCAACTTTAAAGGAAGTAAGAAGATTCTCCTACTTACAGCAACTTCTCGTG 4020

PMP1615_cps GGGCTTATCCCAACTTTAAAGGAAGTAAGAAGATTCTCCTACTTACAGCAACTTCTCGTG 4020

************************************************************

PMP1612_cps TCGAAAAGGTACTGGATAGATTAATAGAATCAAATGAGGTTGTTGGGGAGTTGGTAGCCG 4080

PMP1615_cps TCGAAAAGGTACTGGATAGATTAATAGAATCAAATGAGGTTGTTGGGGAGTTGGTAGCCG 4080

************************************************************

PMP1612_cps TCAGTGTCTTAGATAAACCAGATTTTCAGCATGATTATTTAAAGGTAGTAGCAGAGAGGG 4140

PMP1615_cps TCAGTGTCTTAGATAAACCAGATTTTCAGCATGATTATTTAAAGGTAGTAGCAGAGAGGG 4140

************************************************************

PMP1612_cps AGATAGTAAACTTTGCGACTTATGAGGTGGTCGATGAAGTCTTTATCAATCTTCCAAGTG 4200

PMP1615_cps AGATAGTAAACTTTGCGACTTATGAGGTGGTCGATGAAGTCTTTATCAATCTTCCAAGTG 4200

************************************************************

PMP1612_cps AAAAATACAATATTGGAGAGCTTGTTTCTCAGTTTGAAACGATGGGAATTGATGTAACAG 4260

PMP1615_cps AAAAATACAATATTGGAGAGCTTGTTTCTCAGTTTGAAACGATGGGAATTGATGTAACAG 4260

************************************************************

PMP1612_cps TTAATCTAAATGCTTTTGATCGTAGTTTGGCACGTAACAAGCAAATTCGTAAGATGGCAG 4320

PMP1615_cps TTAATCTAAATGCTTTTGATCGTAGTTTGGCACGTAACAAGCAAATTCGTAAGATGGCAG 4320

************************************************************

PMP1612_cps GATTAAACGTTGTGACTTTTTCTACAACATTTTATAAGACTAGTCATGTAATTGCTAAGC 4380

PMP1615_cps GATTAAACGTTGTGACTTTTTCTACAACATTTTATAAGACTAGTCATGTAATTGCTAAGC 4380

************************************************************

PMP1612_cps GGATTATTGATATCATGGGTGCATTGGTAGGGCTGATACTATGTGGTTTAGTCAGTATTG 4440

PMP1615_cps GGATTATTGATATCATGGGTGCATTGGTAGGGCTGATACTATGTGGTTTAGTCAGTATTG 4440

************************************************************

PMP1612_cps TATTGGTTCCTTTGATTCGAAAGGATGGGGGCTCTGCTATTTTTGCTCAGATGCGTATAG 4500

PMP1615_cps TATTGGTTCCTTTGATTCGAAAGGATGGGGGCTCTGCTATTTTTGCTCAGATGCGTATAG 4500

************************************************************

PMP1612_cps GAAAAAATGGTCGTCAGTTCACTTTTTATAAGTTTCGCTCTATGTGTGTAGATGCCGAGG 4560

PMP1615_cps GAAAAAATGGTCGTCAGTTCACTTTTTATAAGTTTCGCTCTATGTGTGTAGATGCCGAGG 4560

************************************************************

PMP1612_cps CGAAAAAAAGAGAACTCATGGAACAAAATACTATGCAGGGTGGAATGTTTAAGGTGGACG 4620

PMP1615_cps CGAAAAAAAGAGAACTCATGGAACAAAATACTATGCAGGGTGGAATGTTTAAGGTGGACG 4620

************************************************************

PMP1612_cps ATGATCCTCGTATCACGAAAATTGGTCGTTTTATACGGAAGACTAGCTTGGACGAGCTAC 4680

PMP1615_cps ATGATCCTCGTATCACGAAAATTGGTCGTTTTATACGGAAGACTAGCTTGGACGAGCTAC 4680

************************************************************

PMP1612_cps CACAGTTTTATAATGTTCTAAAGGGAGATATGAGTTTGGTTGGCACACGGCCACCAACAG 4740

PMP1615_cps CACAGTTTTATAATGTTCTAAAGGGAGATATGAGTTTGGTTGGCACACGGCCACCAACAG 4740

************************************************************

PMP1612_cps TGGACGAGTATGAACACTATACCCCAGAACAAAAACGTCGGCTAAGTTTTAAACCTGGTA 4800

PMP1615_cps TGGACGAGTATGAACACTATACCCCAGAACAAAAACGTCGGCTAAGTTTTAAACCTGGTA 4800

************************************************************

PMP1612_cps TAACAGGCTTATGGCAGATCAGTGGACGAAGTGAGATTAAGAATTTTGATGAAGTTGTCA 4860

PMP1615_cps TAACAGGCTTATGGCAGATCAGTGGACGAAGTGAGATTAAGAATTTTGATGAAGTTGTCA 4860

************************************************************

PMP1612_cps AATTAGATGTAGCCTATATAGACGATTGGACAATTTGGAAAGATATTGAGATTTTATTGA 4920

PMP1615_cps AATTAGATGTAGCCTATATAGACGATTGGACAATTTGGAAAGATATTGAGATTTTATTGA 4920

************************************************************

PMP1612_cps AGACAGTTAAAGTTGTATTGATGAAGGATGGAGCGAAGTAGATTGATAGATGTAAAAATC 4980

PMP1615_cps AGACAGTTAAAGTTGTATTGATGAAGGATGGAGCGAAGTAGATTGATAGATGTAAAAATC 4980

************************************************************

PMP1612_cps ATTGTGGCAACGCATAAAGAGGTTAAAATGCCTCAAGACAATAGTCTTTACCTTCCAATA 5040

PMP1615_cps ATTGTGGCAACGCATAAAGAGGTTAAAATGCCTCAAGACAATAGTCTTTACCTTCCAATA 5040

************************************************************

PMP1612_cps CATGTTGGGAGAGACGGTAAATCAGATATTGGTTTTATCGGTGATAATACTGGCGATAAT 5100

PMP1615_cps CATGTTGGGAGAGACGGTAAATCAGATATTGGTTTTATCGGTGATAATACTGGCGATAAT 5100

************************************************************

PMP1612_cps ATATCCTCTCTAAATCCATATTATTGTGAGTTGACGGGACTTTATTGGGCATGGAAGAAT 5160

PMP1615_cps ATATCCTCTCTAAATCCATATTATTGTGAGTTGACGGGACTTTATTGGGCATGGAAGAAT 5160

************************************************************

PMP1612_cps CTTGATTATAATTACTTAGGTTTAGTTCATTACCGTCGTTATTTTACAAATAAATCTCAA 5220

PMP1615_cps CTTGATTATAATTACTTAGGTTTAGTTCATTACCGTCGTTATTTTACAAATAAATCTCAA 5220

************************************************************

PMP1612_cps GGGTATAATGAAAATGTCAATATGGATGACGTCATTTTGTCTCGATCTAATGTTGAAATA 5280

PMP1615_cps GGGTATAATGAAAATGTCAATATGGATGACGTCATTTTGTCTCGATCTAATGTTGAAATA 5280

************************************************************

PMP1612_cps TTATTAGAGAAATCTGACATAATAGTTCCAAAGAAGCGAAAGTATTATATTGAAACTCTT 5340

PMP1615_cps TTATTAGAGAAATCTGACATAATAGTTCCAAAGAAGCGAAAGTATTATATTGAAACTCTT 5340

************************************************************

PMP1612_cps TATTCACATTATGCCCATACCCTTAACGGAGAACATCTGGATCTTGCTAGGAAAATTATT 5400

PMP1615_cps TATTCACATTATGCCCATACCCTTAACGGAGAACATCTGGATCTTGCTAGGAAAATTATT 5400

************************************************************

PMP1612_cps GAGCAAAATAGTTCAGAGTATCTTTCATCCTTTGATAAAGTGATGAAACAAAGAAGCGGT 5460

PMP1615_cps GAGCAAAATAGTTCAGAGTATCTTTCATCCTTTGATAAAGTGATGAAACAAAGAAGCGGT 5460

************************************************************

PMP1612_cps TATATGTTCAATATGTTTATCATGAAAAAAGAACTATTAGATGATTATTTACCGTGGCTT 5520

PMP1615_cps TATATGTTCAATATGTTTATCATGAAAAAAGAACTATTAGATGATTATTTACCGTGGCTT 5520

************************************************************

PMP1612_cps TTTTCTATTCTGGATACTATGTACGAACAGATGGACTTGACCGACTATACTCCATTTGAG 5580

PMP1615_cps TTTTCTATTCTGGATACTATGTACGAACAGATGGACTTGACCGACTATACTCCATTTGAG 5580

************************************************************

PMP1612_cps TCACGTTTATTCGGGCGAGTTAGTGAGTTGTTGTTTAATGTTTGGTTATGTAAACAAGGA 5640

PMP1615_cps TCACGTTTATTCGGGCGAGTTAGTGAGTTGTTGTTTAATGTTTGGTTATGTAAACAAGGA 5640

************************************************************

PMP1612_cps ATAACGCCTAAAGAGGTACCATTTATGTACATGGAGAGAGTGGATTTGTTTGAAAAAGGA 5700

PMP1615_cps ATAACGCCTAAAGAGGTACCATTTATGTACATGGAGAGAGTGGATTTGTTTGAAAAAGGA 5700

************************************************************

PMP1612_cps AAATCTTTTTTAATGGCTAAATTTTTTGGAAAGAAGTATGGACAGAGTTTTTAAATTATG 5760

PMP1615_cps AAATCTTTTTTAATGGCTAAATTTTTTGGAAAGAAGTATGGACAGAGTTTTTAAATTATG 5760

************************************************************

PMP1612_cps GTTTTGTTATTACTTACTTTTGCATTCTTTTTAGTTTTCTCTGTGCTGTCAATAAGTTTA 5820

PMP1615_cps GTTTTGTTATTACTTACTTTTGCATTCTTTTTAGTTTTCTCTGTGCTGTCAATAAGTTTA 5820

************************************************************

PMP1612_cps AGTGTTATAGGGCTAGTAAATGACAAAAAGCGTTCAAAAATCTATTTACTACTAATTAGT 5880

PMP1615_cps AGTGTTATAGGGCTAGTAAATGACAAAAAGCGTTCAAAAATCTATTTACTACTAATTAGT 5880

************************************************************

PMP1612_cps TTTGCAGTATCAATTGTTGCGCTGCGTTATATTCCTCATCATATGGCTGATGGCGCTTTT 5940

PMP1615_cps TTTGCAGTATCAATTGTTGCGCTGCGTTATATTCCTCATCATATGGCTGATGGCGCTTTT 5940

************************************************************

PMP1612_cps CATTTTCGTGCGACTACGGCCCTTATTCGCTATGATAGTATTTTCGAAATGTTTTAAGAA 6000

PMP1615_cps CATTTTCGTGCGACTACGGCCCTTATTCGCTATGATAGTATTTTCGAAATGTTTTAAGAA 6000

************************************************************

PMP1612_cps TTTTCTAGTGGATGGAATGTGGGAAGATATGATTATGGTTCTATGCCAGTTTTTACTTCA 6060

PMP1615_cps TTTTCTAGTGGATGGAATGTGGGAAGATATGATTATGGTTCTATGCCAGTTTTTACTTCA 6060

************************************************************

PMP1612_cps TTAATGTATCTTATTCGAAACACTCATCACTATAGTTTACTGAGTTTTATTTCAGCTTTT 6120

PMP1615_cps TTAATGTATCTTATTCGAAACACTCATCACTATAGTTTACTGAGTTTTATTTCAGCTTTT 6120

************************************************************

PMP1612_cps ATAACTTACTTTAGTTTTGGTTACGTAGTCGTTGATCTATTTAAAGACTTGGACAAGGTT 6180

PMP1615_cps ATAACTTACTTTAGTTTTGGTTACGTAGTCGTTGATCTATTTAAAGACTTGGACAAGGTT 6180

************************************************************

PMP1612_cps TCTAAACTATCGTATGCTACAGTATTAATTGCTGTACTATGTTTAAATAATTATAGATAT 6240

PMP1615_cps TCTAAACTATCGTATGCTACAGTATTAATTGCTGTACTATGTTTAAATAATTATAGATAT 6240

************************************************************

PMP1612_cps ACAACTGGTGGAATGAGATTTTGTATTGTAGTTGCTTTGATGATGCTTTTATTGATTTTA 6300

PMP1615_cps ACAACTGGTGGAATGAGATTTTGTATTGTAGTTGCTTTGATGATGCTTTTATTGATTTTA 6300

************************************************************

PMP1612_cps GTAAGACGGACTTACTCATAAAAAATATTTTTCAATTCTTTTCAAGTTTGCCATTTACAT 6360

PMP1615_cps GTAAGACGGACTTACTCATAAAAAATATTTTTCAATTCTTTTCAAGTTTGCCATTTACAT 6360

************************************************************

PMP1612_cps AATAAGACTATGTGCTTTTATTTTATTTTTTGGAGGATTATATGGGACTATCTACCGTGA 6420

PMP1615_cps AATAAGACTATGTGCTTTTATTTTATTTTTTGGAGGATTATATGGGACTATCTACCGTGA 6420

************************************************************

PMP1612_cps CATTGTTTAAAAATTTAAAGTTCTCAGATAGTAAATTTATTAAATTGGAAGGAGAGCTCC 6480

PMP1615_cps CATTGTTTAAAAATTTAAAGTTCTCAGATAGTAAATTTATTAAATTGGAAGGAGAGCTCC 6480

************************************************************

PMP1612_cps TTCTTAAATATCAAGAGTATTTATTAAAAATCATGGAAGATATTGTAACAGTGTGTGAGG 6540

PMP1615_cps TTCTTAAATATCAAGAGTATTTATTAAAAATCATGGAAGATATTGTAACAGTGTGTGAGG 6540

************************************************************

PMP1612_cps AAGAAGGGTTATATTATTCACTTTCTGGTGGGAGTGCATTAGGGGCTTACCGACACAAAG 6600

PMP1615_cps AAGAAGGGTTATATTATTCACTTTCTGGTGGGAGTGCATTAGGGGCTTACCGACACAAAG 6600

************************************************************

PMP1612_cps GGTTTATTCCATGGGATGATGATATGGACATATTTATGCTAGGAAGTGAGCGGGAGATTT 6660

PMP1615_cps GGTTTATTCCATGGGATGATGATATGGACATATTTATGCTAGGAAGTGAGCGGGAGATTT 6660

************************************************************

PMP1612_cps TCTTTCAAAAATTTTCTCAGAAATTTTCTGATAAATATTGGATACATAATTCACAAACAC 6720

PMP1615_cps TCTTTCAAAAATTTTCTCAGAAATTTTCTGATAAATATTGGATACATAATTCACAAACAC 6720

************************************************************

PMP1612_cps CAAACTATGGCATGCCCATTGGTCGTATCAGACAAAAAGGAACAGTTTTACGTGGTCGAG 6780

PMP1615_cps CAAACTATGGCATGCCCATTGGTCGTATCAGACAAAAAGGAACAGTTTTACGTGGTCGAG 6780

************************************************************

PMP1612_cps AGGATGTTGGAGTTGAAGAATGTGGATTTTTTATTGATATTTTTTGGCTTGAAAATGTTC 6840

PMP1615_cps AGGATGTTGGAGTTGAAGAATGTGGATTTTTTATTGATATTTTTTGGCTTGAAAATGTTC 6840

************************************************************

PMP1612_cps CTAATTCAAAAATATTGAGACAACTCCACGGTTTTCTCTGCATGGCGATTGGATTGCTAC 6900

PMP1615_cps CTAATTCAAAAATATTGAGACAACTCCACGGTTTTCTCTGCATGGCGATTGGATTGCTAC 6900

************************************************************

PMP1612_cps TATCATGTAGAAATTTTTATAAAAATCGTCAGCTGATGCTGGAGATTATGAAGGAACATA 6960

PMP1615_cps TATCATGTAGAAATTTTTATAAAAATCGTCAGCTGATGCTGGAGATTATGAAGGAACATA 6960

************************************************************

PMP1612_cps AAGAAGTAAGGCTTGTTTTTCGAATAAAACTAGTTTTAGGGTTTCTAATTAGTTTTATAT 7020

PMP1615_cps AAGAAGTAAGGCTTGTTTTTCGAATAAAACTAGTTTTAGGGTTTCTAATTAGTTTTATAT 7020

************************************************************

PMP1612_cps CTTTGAGACAATTTACAAGACTTACGGAAAGGATCTACTCATTGTGCAAGAATAATGAAT 7080

PMP1615_cps CTTTGAGACAATTTACAAGACTTACGGAAAGGATCTACTCATTGTGCAAGAATAATGAAT 7080

************************************************************

PMP1612_cps CAAGATACCTTAGTGTTCCTTCAGGAAGAAAACATTATTTTGGCGAGATGTTTATAAGAG 7140

PMP1615_cps CAAGATACCTTAGTGTTCCTTCAGGAAGAAAACATTATTTTGGCGAGATGTTTATAAGAG 7140

************************************************************

PMP1612_cps AAGATATGCAGTTAACTAGGAAACTCAATTTTGAAGGACATAAGTGGAATGTTCCAAATA 7200

PMP1615_cps AAGATATGCAGTTAACTAGGAAACTCAATTTTGAAGGACATAAGTGGAATGTTCCAAATA 7200

************************************************************

PMP1612_cps ATATTGAGCATTATTTAACTGTAATGTACGGTGATTATATGAAAATACCTGCAGTTGAAG 7260

PMP1615_cps ATATTGAGCATTATTTAACTGTAATGTACGGTGATTATATGAAAATACCTGCAGTTGAAG 7260

************************************************************

PMP1612_cps ATAGGGAATCACATATCATTTTAGAAATCTCCTTCCCTAACGAGTAAACAGTACTTTCAT 7320

PMP1615_cps ATAGGGAATCACATATCATTTTAGAAATCTCCTTCCCTAACGAGTAAACAGTACTTTCAT 7320

************************************************************

PMP1612_cps TTTTTATATGTAAGGAATTAATTTAATGAAAAAAATAGCAATTGTTAGATATAATCTAAG 7380

PMP1615_cps TTTTTATATGTAAGGAATTAATTTAATGAAAAAAATAGCAATTGTTAGATATAATCTAAG 7380

************************************************************

PMP1612_cps TAAAATTGGTGGAGCAGAAAAAGTAGCTATTAATATGGCTAATGAATTGTCACAATACTA 7440

PMP1615_cps TAAAATTGGTGGAGCAGAAAAAGTAGCTATTAATATGGCTAATGAATTGTCACAATACTA 7440

************************************************************

PMP1612_cps TGATGTAAAACTGTTATCTATTCTATTGGATGAGGATGGCTTTATAAATTATGATATTAA 7500

PMP1615_cps TGATGTAAAACTGTTATCTATTCTATTGGATGAGGATGGCTTTATAAATTATGATATTAA 7500

************************************************************

PMP1612_cps CCCTAATGTGACATTAATAAATTTTCACAAGGGTGATCTTAGAATTAGAACTGCTACATT 7560

PMP1615_cps CCCTAATGTGACATTAATAAATTTTCACAAGGGTGATCTTAGAATTAGAACTGCTACATT 7560

************************************************************

PMP1612_cps AAAATTAACAGGTAAATTAAGGAACTATATTAAAAGAGAAAAAATTGAAGTGATTTTTTC 7620

PMP1615_cps AAAATTAACAGGTAAATTAAGGAACTATATTAAAAGAGAAAAAATTGAAGTGATTTTTTC 7620

************************************************************

PMP1612_cps TATAACCCCATTAACAAATACTATGGTTAGATTAGCTACTCTAGGATTAAATGTGAAAAT 7680

PMP1615_cps TATAACCCCATTAACAAATACTATGGTTAGATTAGCTACTCTAGGATTAAATGTGAAAAT 7680

************************************************************

PMP1612_cps AGTTTTCTGTGATCATCATAGCTTAGAATTTCGTGATTTTAGAGGTAGGGAAGTGCAGAG 7740

PMP1615_cps AGTTTTCTGTGATCATCATAGCTTAGAATTTCGTGATTTTAGAGGTAGGGAAGTGCAGAG 7740

************************************************************

PMP1612_cps ATTTGTAGGAGCTAAATTTTTTGACAAGATTGTCACTTTAACAGAAGAAGATAGAATAAA 7800

PMP1615_cps ATTTGTAGGAGCTAAATTTTTTGACAAGATTGTCACTTTAACAGAAGAAGATAGAATAAA 7800

************************************************************

PMP1612_cps ATACTCCGATAAATATAATATACCTATAAATAAAGTAAATGCTATTTATAATTGGATTGA 7860

PMP1615_cps ATACTCCGATAAATATAATATACCTATAAATAAAGTAAATGCTATTTATAATTGGATTGA 7860

************************************************************

PMP1612_cps TGAAGAAGATTCTGAAAATACCCCATTTGATAATGAGACAAATAAAATAATAACGGTAGG 7920

PMP1615_cps TGAAGAAGATTCTGAAAATACCCCATTTGATAATGAGACAAATAAAATAATAACGGTAGG 7920

************************************************************

PMP1612_cps TCGATTTCACAGTCAAAAGGGATATGATTATCTTGCCGAAGTAGCTATAAAAGTATTATC 7980

PMP1615_cps TCGATTTCACAGTCAAAAGGGATATGATTATCTTGCCGAAGTAGCTATAAAAGTATTATC 7980

************************************************************

PMP1612_cps ACAACATCCAGACTGGCAGTGGGATATATATGGTTCAGGGGATAAACTTATTGAACAGGA 8040

PMP1615_cps ACAACATCCAGACTGGCAGTGGGATATATATGGTTCAGGGGATAAACTTATTGAACAGGA 8040

************************************************************

PMP1612_cps TCTAAAAAGAAAACTAGAAGAAGGTTGTGTTTCTTCACAAGTTAATTTTAAAGGGAATGT 8100

PMP1615_cps TCTAAAAAGAAAACTAGAAGAAGGTTGTGTTTCTTCACAAGTTAATTTTAAAGGGAATGT 8100

************************************************************

PMP1612_cps AAAGGGAACTGAAAATATTTATCCCAATCATAGTATCTATGTCATGACTTCTCGCCATGA 8160

PMP1615_cps AAAGGGAACTGAAAATATTTATCCCAATCATAGTATCTATGTCATGACTTCTCGCCATGA 8160

************************************************************

PMP1612_cps GGGCTTACCTTTAGTCCTATTAGAAGCACAACAATACAATCTTCCTATTGTTAGCTTCAG 8220

PMP1615_cps GGGCTTACCTTTAGTCCTATTAGAAGCACAACAATACAATCTTCCTATTGTTAGCTTCAG 8220

************************************************************

PMP1612_cps ATGTCCAACGGGACCTAGTGAGATTGTTGAAGATGGAGTCAATGGATTTTTGATTGATTG 8280

PMP1615_cps ATGTCCAACGGGACCTAGTGAGATTGTTGAAGATGGAGTCAATGGATTTTTGATTGATTG 8280

************************************************************

PMP1612_cps CTATGACGTGTATCAGATGAGTGAGAAATTGCTTGAATTGATGAAAAATGATGATTTGCG 8340

PMP1615_cps CTATGACGTGTATCAGATGAGTGAGAAATTGCTTGAATTGATGAAAAATGATGATTTGCG 8340

************************************************************

PMP1612_cps ACAATCTTTCTCAGAACATGCCAAAGACAATATAGATAAATTTGATAAAAATGAAATTCT 8400

PMP1615_cps ACAATCTTTCTCAGAACATGCCAAAGACAATATAGATAAATTTGATAAAAATGAAATTCT 8400

************************************************************

PMP1612_cps TAATCAGTGGATAGAATTGATTGATACAATTTAGGAGGTAAGATGCAAGAGTGTTTATTG 8460

PMP1615_cps TAATCAGTGGATAGAATTGATTGATACAATTTAGGAGGTAAGATGCAAGAGTGTTTATTG 8460

************************************************************

PMP1612_cps ACAATTATAATGCCTAGTTATAATATTCAGGACTATATTTCCAAAGGAATCGAGTCATTC 8520

PMP1615_cps ACAATTATAATGCCTAGTTATAATATTCAGGACTATATTTCCAAAGGAATCGAGTCATTC 8520

************************************************************

PMP1612_cps CAGCAAGTACACCTAGATTACAAACAAAAATTTGAGATCTTAATTGTGAATGATGGAAGT 8580

PMP1615_cps CAGCAAGTACACCTAGATTACAAACAAAAATTTGAGATCTTAATTGTGAATGATGGAAGT 8580

************************************************************

PMP1612_cps ACGGACGACACAGCCAAAGTAGCAGAAGAGACCCTAAGAAAAGATTTATTGCTGAATGGT 8640

PMP1615_cps ACGGACGACACAGCCAAAGTAGCAGAAGAGACCCTAAGAAAAGATTTATTGCTGAATGGT 8640

************************************************************

PMP1612_cps CGTATTATCACAAAGGAAAATGGAGGCCATGGCTCAACAATCAATCGTGGTATCCAGGAA 8700

PMP1615_cps CGTATTATCACAAAGGAAAATGGAGGCCATGGCTCAACAATCAATCGTGGTATCCAGGAA 8700

************************************************************

PMP1612_cps GCAAAGGGAAAATTCTTTAAAGTTATTGATGGGGATGACTGGGTTATTCCATCAGAATTT 8760

PMP1615_cps GCAAAGGGAAAATTCTTTAAAGTTATTGATGGGGATGACTGGGTTATTCCATCAGAATTT 8760

************************************************************

PMP1612_cps GAAAAGTTTTTAGATGACCTTACAGTTACTGAAGTGGATATGATCTTGACAGATTTTACA 8820

PMP1615_cps GAAAAGTTTTTAGATGACCTTACAGTTACTGAAGTGGATATGATCTTGACAGATTTTACA 8820

************************************************************

PMP1612_cps GAACAACATGTTTACAACAATACTACTGTTCGAAATGATTTTGTTGAAAAGTATGAGGTT 8880

PMP1615_cps GAACAACATGTTTACAACAATACTACTGTTCGAAATGATTTTGTTGAAAAGTATGAGGTT 8880

************************************************************

PMP1612_cps GGTAAGGAATATTCTGGAATTCCAGAGAAACGGATTCCAATGCACTCGGTAACTTATAGA 8940

PMP1615_cps GGTAAGGAATATTCTGGAATTCCAGAGAAACGGATTCCAATGCACTCGGTAACTTATAGA 8940

************************************************************

PMP1612_cps ACATCTATCCTAGTTGAGAATGAAATTCGTTTAAGTGAAAAGACATTTTATGTTGATATT 9000

PMP1615_cps ACATCTATCCTAGTTGAGAATGAAATTCGTTTAAGTGAAAAGACATTTTATGTTGATATT 9000

************************************************************

PMP1612_cps CAGTACACTCTTTTTCCTTTAGAGTATGTTCATAGTTTCTGTTATTGGAATTATGATGTA 9060

PMP1615_cps CAGTACACTCTTTTTCCTTTAGAGTATGTTCATAGTTTCTGTTATTGGAATTATGATGTA 9060

************************************************************

PMP1612_cps TACCAATACTATATCGGACGACCCGAGCAGAGTATGAATATTGAGAGTATGAAACGAAAT 9120

PMP1615_cps TACCAATACTATATCGGACGACCCGAGCAGAGTATGAATATTGAGAGTATGAAACGAAAT 9120

************************************************************

PMP1612_cps GTTCGTCACCATTTGACTGTAACAAATTCTGTGCTGACTTATTTCTCAAAAATTGCGGAT 9180

PMP1615_cps GTTCGTCACCATTTGACTGTAACAAATTCTGTGCTGACTTATTTCTCAAAAATTGCGGAT 9180

************************************************************

PMP1612_cps GATCCTGTCTTAAAGAAAGTGGTTGCAGATACTTTAGTTTATCTTATCAGTTTGCAGATA 9240

PMP1615_cps GATCCTGTCTTAAAGAAAGTGGTTGCAGATACTTTAGTTTATCTTATCAGTTTGCAGATA 9240

************************************************************

PMP1612_cps GATTTGTCATGGATGGTTGAGGACTCAAAGACACTATCAGAAGAGTTATACAGACAAATT 9300

PMP1615_cps GATTTGTCATGGATGGTTGAGGACTCAAAGACACTATCAGAAGAGTTATACAGACAAATT 9300

************************************************************

PMP1612_cps GAGCAGAGTTCTTATGAGTATATCCCTACGAAAAAATTTGATAGATTGTCTTATTTGAAC 9360

PMP1615_cps GAGCAGAGTTCTTATGAGTATATCCCTACGAAAAAATTTGATAGATTGTCTTATTTGAAC 9360

************************************************************

PMP1612_cps TATAAGTTTCACTATTTTCTAGACTTTGTTTTCAATCCAGTATTGAAAAAATATTCTAAA 9420

PMP1615_cps TATAAGTTTCACTATTTTCTAGACTTTGTTTTCAATCCAGTATTGAAAAAATATTCTAAA 9420

************************************************************

PMP1612_cps AAGAAAGAAAAAGAGAGAGGAATTTAGAATGTTCATTAGTGTTGTTGTTCCGGTTTATAA 9480

PMP1615_cps AAGAAAGAAAAAGAGAGAGGAATTTAGAATGTTCATTAGTGTTGTTGTTCCGGTTTATAA 9480

************************************************************

PMP1612_cps TGTTTTTGACTATTTACACTTTGCCATGGATAGTCTGATAAAGCAAACGTATCAAAATTT 9540

PMP1615_cps TGTTTTTGACTATTTACACTTTGCCATGGATAGTCTGATAAAGCAAACGTATCAAAATTT 9540

************************************************************

PMP1612_cps TGAAGTAATCCTTGTAAATGATGGTTCAACAGATAACTCTCCTCAGTTATGTGAAGAGTA 9600

PMP1615_cps TGAAGTAATCCTTGTAAATGATGGTTCAACAGATAACTCTCCTCAGTTATGTGAAGAGTA 9600

************************************************************

PMP1612_cps TGCTAAGCAATATGAAAACGTCTCTGTTTTTCATAAAGAAAATGGAGGGTTGTCTGATGC 9660

PMP1615_cps TGCTAAGCAATATGAAAACGTCTCTGTTTTTCATAAAGAAAATGGAGGGTTGTCTGATGC 9660

************************************************************

PMP1612_cps TCGTAATTTTGGAGTTTCAAAAGCATCATCAGATTGGATTTTCTTTCTAGATCCAGACGA 9720

PMP1615_cps TCGTAATTTTGGAGTTTCAAAAGCATCATCAGATTGGATTTTCTTTCTAGATCCAGACGA 9720

************************************************************

PMP1612_cps TTATTTGGAAGATTATACTCTAGAATTGATAGTGAAAATTCAACAGGAATATCAAGCAAA 9780

PMP1615_cps TTATTTGGAAGATTATACTCTAGAATTGATAGTGAAAATTCAACAGGAATATCAAGCAAA 9780

************************************************************

PMP1612_cps CTTGATTTCAACCAAAGTAAAGGCAACTTCTAAATATAATGATTATAGTCTTTATCAACT 9840

PMP1615_cps CTTGATTTCAACCAAAGTAAAGGCAACTTCTAAATATAATGATTATAGTCTTTATCAACT 9840

************************************************************

PMP1612_cps TGAGGAGTCAGATTATAAAGATTTGTTTGTCGTTACAAAAGAGAAGGCTCTTGAGCTAAT 9900

PMP1615_cps TGAGGAGTCAGATTATAAAGATTTGTTTGTCGTTACAAAAGAGAAGGCTCTTGAGCTAAT 9900

************************************************************

PMP1612_cps GCTAGACGACAAAATTGCGACAGTTTCTGCTTGTGCTAAGCTTTATCACAAGAATATCTT 9960

PMP1615_cps GCTAGACGACAAAATTGCGACAGTTTCTGCTTGTGCTAAGCTTTATCACAAGAATATCTT 9960

************************************************************

PMP1612_cps GGAAAAAGTTCCATTTTCAGTTGGAAAAATCTATGAAGATTTTTATGTGGTCGCGGATCA 10020

PMP1615_cps GGAAAAAGTTCCATTTTCAGTTGGAAAAATCTATGAAGATTTTTATGTGGTCGCGGATCA 10020

************************************************************

PMP1612_cps TCTTGCCTTAGCAGATAAAATTGTAATTAGTCCACTTGAAACGTATAACTATTACCGCAG 10080

PMP1615_cps TCTTGCCTTAGCAGATAAAATTGTAATTAGTCCACTTGAAACGTATAACTATTACCGCAG 10080

************************************************************

PMP1612_cps AGAAGGTAGTATTGTTCGTTCGACTTTTACTGAGAAAAGATATGATTTTTTTGATGCTGT 10140

PMP1615_cps AGAAGGTAGTATTGTTCGTTCGACTTTTACTGAGAAAAGATATGATTTTTTTGATGCTGT 10140

************************************************************

PMP1612_cps TTCGAAAAACGAAGAAGTTATAAATAAAGAATACACACAAAGTTTAGAATTACAACAATC 10200

PMP1615_cps TTCGAAAAACGAAGAAGTTATAAATAAAGAATACACACAAAGTTTAGAATTACAACAATC 10200

************************************************************

PMP1612_cps TCTGCAAGCGAAAAAATTACGAGGAGGTTTTGTTGTAATTGGTGCGAAAGCTGACTCTGG 10260

PMP1615_cps TCTGCAAGCGAAAAAATTACGAGGAGGTTTTGTTGTAATTGGTGCGAAAGCTGACTCTGG 10260

************************************************************

PMP1612_cps TTTAACAGATTTTTCGAAAGATAGAGACCTATTAAAAGTTGATTTTAAAAACATGTTATT 10320

PMP1615_cps TTTAACAGATTTTTCGAAAGATAGAGACCTATTAAAAGTTGATTTTAAAAACATGTTATT 10320

************************************************************

PMP1612_cps AAATAAAAAAATATCATGGAAACTAAAATTAAAATATACAATATTTATGCTATCATCTAA 10380

PMP1615_cps AAATAAAAAAATATCATGGAAACTAAAATTAAAATATACAATATTTATGCTATCATCTAA 10380

************************************************************

PMP1612_cps AATGTATTTAAGATTAAGGTGACTGTTTCAAGAATCAAGCATCTCTATTAGTTTTTAAAA 10440

PMP1615_cps AATGTATTTAAGATTAAGGTGACTGTTTCAAGAATCAAGCATCTCTATTAGTTTTTAAAA 10440

************************************************************

PMP1612_cps TTATAAATAGCTGTTCGGATGTAAGTGTTGTAATAGTGTTTAGTATTTCTAGTTCATTAT 10500

PMP1615_cps TTATAAATAGCTGTTCGGATGTAAGTGTTGTAATAGTGTTTAGTATTTCTAGTTCATTAT 10500

************************************************************

PMP1612_cps TATAATCGGGAAAGAATTTAATACAGCTACTTATTTGAAAGGGAAATTCATGAGACAAGC 10560

PMP1615_cps TATAATCGGGAAAGAATTTAATACAGCTACTTATTTGAAAGGGAAATTCATGAGACAAGC 10560

************************************************************

PMP1612_cps ATATCTAATAATAGCACATAATAAGTTTGAATAGTTGAAATTTTTAATTTCTCTGTTGGA 10620

PMP1615_cps ATATCTAATAATAGCACATAATAAGTTTGAATAGTTGAAATTTTTAATTTCTCTGTTGGA 10620

************************************************************

PMP1612_cps TTATAAGGAACATAATATTTTTATTATTGTGGACAGCAAAGTTAATGCTGAGGAGTCTAC 10680

PMP1615_cps TTATAAGGAACATAATATTTTTATTATTGTGGACAGCAAAGTTAATGCTGAGGAGTCTAC 10680

************************************************************

PMP1612_cps GATTAATCACTATACTTCCCATCATTATAACTTCCTATGTGACATGGATATTTTAACTCT 10740

PMP1615_cps GATTAATCACTATACTTCCCATCATTATAACTTCCTATGTGACATGGATATTTTAACTCT 10740

************************************************************

PMP1612_cps ATTGTTACCTATTTTGTTAGTCACTTTAGGAGTTGCTAGTTATGGCATTAAGGAAATTTA 10800

PMP1615_cps ATTGTTACCTATTTTGTTAGTCACTTTAGGAGTTGCTAGTTATGGCATTAAGGAAATTTA 10800

************************************************************

PMP1612_cps AGCTAATCGAAAAAAGAGCCACAGAATTTTTGGGGTATCTGTAAAACTTAAAATGAAGTA 10860

PMP1615_cps AGCTAATCGAAAAAAGAGCCACAGAATTTTTGGGGTATCTGTAAAACTTAAAATGAAGTA 10860

************************************************************

PMP1612_cps TAGTTTATTTTGAGTTTAATAAGCGATTAAAAGGTAGGGGGAATATTATTGAAGATTAAC 10920

PMP1615_cps TAGTTTATTTTGAGTTTAATAAGCGATTAAAAGGTAGGGGGAATATTATTGAAGATTAAC 10920

************************************************************

PMP1612_cps TTAATGAATATTGTTGATACGTTACTGCTTTTGCTAATAATTATAAAAACAAACTCTATT 10980

PMP1615_cps TTAATGAATATTGTTGATACGTTACTGCTTTTGCTAATAATTATAAAAACAAACTCTATT 10980

************************************************************

PMP1612_cps TATTTGTATATAGGTGTAGAGAACAAATTAAATTTTTATCTGATTGTTGTGATTACAATT 11040

PMP1615_cps TATTTGTATATAGGTGTAGAGAACAAATTAAATTTTTATCTGATTGTTGTGATTACAATT 11040

************************************************************

PMP1612_cps GCAGTTTTATTGGAAGTAATTAGCGGTAGATTATCTCTCCAATATTTAAAAAAAATGCTA 11100

PMP1615_cps GCAGTTTTATTGGAAGTAATTAGCGGTAGATTATCTCTCCAATATTTAAAAAAAATGCTA 11100

************************************************************

PMP1612_cps TTAGTTATTGTTCTTTATTTTTTTGCAATTATTATTAATATTTTAGTTTCGGCTTCTGTC 11160

PMP1615_cps TTAGTTATTGTTCTTTATTTTTTTGCAATTATTATTAATATTTTAGTTTCGGCTTCTGTC 11160

************************************************************

PMP1612_cps ATATCGTCAAATATACTAACATACTATTTTATTGTTGTGCCATTAATGCTAATACTAGTA 11220

PMP1615_cps ATATCGTCAAATATACTAACATACTATTTTATTGTTGTGCCATTAATGCTAATACTAGTA 11220

************************************************************

PMP1612_cps ATGTATAAATATTATAAAAATACATTAACAAATTTTTTAGCTATCTTTGTGAGGATTGTT 11280

PMP1615_cps ATGTATAAATATTATAAAAATACATTAACAAATTTTTTAGCTATCTTTGTGAGGATTGTT 11280

************************************************************

PMP1612_cps TTAATTTTAGCTGTTATTTCTCTGCTCTTTTGGTGTTTTGGTAGTGTTTTAAATATAATA 11340

PMP1615_cps TTAATTTTAGCTGTTATTTCTCTGCTCTTTTGGTGTTTTGGTAGTGTTTTAAATATAATA 11340

************************************************************

PMP1612_cps AAACCGACAAACTATGTGGTGAGTAGCTGGAGTGGTGGTCAGGTAACTACTAGTTATTAT 11400

PMP1615_cps AAACCGACAAACTATGTGGTGAGTAGCTGGAGTGGTGGTCAGGTAACTACTAGTTATTAT 11400

************************************************************

PMP1612_cps AATCTGTATTTTGAAACGCAAAACGCTTTATTTTTGGGTTATAAAATGATTAGGAATAGC 11460

PMP1615_cps AATCTGTATTTTGAAACGCAAAACGCTTTATTTTTGGGTTATAAAATGATTAGGAATAGC 11460

************************************************************

PMP1612_cps GGTATTTTTGCAGAAGCGCCAATGTGGAGTTTATTGCTAAGTGTTGCTTTAATATTTCAA 11520

PMP1615_cps GGTATTTTTGCAGAAGCGCCAATGTGGAGTTTATTGCTAAGTGTTGCTTTAATATTTCAA 11520

************************************************************

PMP1612_cps GAGTTATTGCTTAAACATAGCACTAGAATATTTGTTTTATTAATGTTGACAATTTTTACA 11580

PMP1615_cps GAGTTATTGCTTAAACATAGCACTAGAATATTTGTTTTATTAATGTTGACAATTTTTACA 11580

************************************************************

PMP1612_cps ACAGCATCAACAACAGGTTTTTTTATAGTTGGTTCATTACTTATTTATAAAGTGATAAAT 11640

PMP1615_cps ACAGCATCAACAACAGGTTTTTTTATAGTTGGTTCATTACTTATTTATAAAGTGATAAAT 11640

************************************************************

PMP1612_cps CAAAAAAGAAGTTGGTTTAAATATATCAACTTGACATCTATCCCTGTATTAATCTTTACT 11700

PMP1615_cps CAAAAAAGAAGTTGGTTTAAATATATCAACTTGACATCTATCCCTGTATTAATCTTTACT 11700

************************************************************

PMP1612_cps TTGGTTAAAGTGTGGGGGGAAAAATCCGATTCCGCTTCAGCAAGTATTCGATATGATGAT 11760

PMP1615_cps TTGGTTAAAGTGTGGGGGGAAAAATCCGATTCCGCTTCAGCAAGTATTCGATATGATGAT 11760

************************************************************

PMP1612_cps TATGTAGCAGGTTTTTTGGCATGGAAAAATCACTTTATTTTTGGTTCAGGTTTATCATCT 11820

PMP1615_cps TATGTAGCAGGTTTTTTGGCATGGAAAAATCACTTTATTTTTGGTTCAGGTTTATCATCT 11820

************************************************************

PMP1612_cps GGGATTAGAGCGATTGAGTCCTATATGGATACCACTATACGAAGTAATTTAGGATACAGT 11880

PMP1615_cps GGGATTAGAGCGATTGAGTCCTATATGGATACCACTATACGAAGTAATTTAGGATACAGT 11880

************************************************************

PMP1612_cps AATAGTTTTTTTGTCATCTTAGCTCAAGGGGGAATAATTTTGGGCGTGCTACACTTTTAT 11940

PMP1615_cps AATAGTTTTTTTGTCATCTTAGCTCAAGGGGGAATAATTTTGGGCGTGCTACACTTTTAT 11940

************************************************************

PMP1612_cps CCTGTTGTTAGTGTACTATTGAAGAGATTTTCATCAAACTCTAAGATGTTAGCTTTATTG 12000

PMP1615_cps CCTGTTGTTAGTGTACTATTGAAGAGATTTTCATCAAACTCTAAGATGTTAGCTTTATTG 12000

************************************************************

PMP1612_cps TTTATAATTCTAATATTTACAGCAATATTTACAGATACACCGTTATTTATCCTGTTTGTT 12060

PMP1615_cps TTTATAATTCTAATATTTACAGCAATATTTACAGATACACCGTTATTTATCCTGTTTGTT 12060

************************************************************

PMP1612_cps GGGATATTTTATGCCTTGATTCTGAATAGAGAGAATACATGAAGAAGGAATATGATATTT 12120

PMP1615_cps GGGATATTTTATGCCTTGATTCTGAATAGAGAGAATACATGAAGAAGGAATATGATATTT 12120

************************************************************

PMP1612_cps TAAAAGTAATTGCCATTTTAATGGTTGTATTAAGCCACAGTACATACTATGTGATTTCGA 12180

PMP1615_cps TAAAAGTAATTGCCATTTTAATGGTTGTATTAAGCCACAGTACATACTATGTGATTTCGA 12180

************************************************************

PMP1612_cps CTAAGTACGGGGGGATTATCAACAATATATAAATCAAAATTTATCGTTGGTATTGTATAA 12240

PMP1615_cps CTAAGTACGGGGGGATTATCAACAATATATAAATCAAAATTTATCGTTGGTATTGTATAA 12240

************************************************************

PMP1612_cps AGTTTTTGATAAAGTAAGAGAAGTATTATATTACTTCCATATGCCACTTTTTATGGCATT 12300

PMP1615_cps AGTTTTTGATAAAGTAAGAGAAGTATTATATTACTTCCATATGCCACTTTTTATGGCATT 12300

************************************************************

PMP1612_cps ATCAGGAGCTTTCTACTATCTTCAGGTTCAAAGAGATAAATGGTTTACTTTAAAATTAAT 12360

PMP1615_cps ATCAGGAGCTTTCTACTATCTTCAGGTTCAAAGAGATAAATGGTTTACTTTAAAATTAAT 12360

************************************************************

PMP1612_cps TGTGCAAAATAAAATAAGGTAAAGAGATTGCTTTTTCCTTTCATTATATTTACTGTCCTT 12420

PMP1615_cps TGTGCAAAATAAAATAAGGTAAAGAGATTGCTTTTTCCTTTCATTATATTTACTGTCCTT 12420

************************************************************

PMP1612_cps TATTCAATACCAATAAAATATATTTCAAATTATTTTGATTTTACAGCTCCTTTTAAAGCA 12480

PMP1615_cps TATTCAATACCAATAAAATATATTTCAAATTATTTTGATTTTACAGCTCCTTTTAAAGCA 12480

************************************************************

PMP1612_cps TTTGTAGGAGAATTTTTCTTAATTGGAAATAGTCATTTATGGTATTTATATGCTTTATTT 12540

PMP1615_cps TTTGTAGGAGAATTTTTCTTAATTGGAAATAGTCATTTATGGTATTTATATGCTTTATTT 12540

************************************************************

PMP1612_cps ATTATTTTTATATTTGCATTCTATACGCTAAAAAAGAAACAAATCTTGCTACTTATGTTG 12600

PMP1615_cps ATTATTTTTATATTTGCATTCTATACGCTAAAAAAGAAACAAATCTTGCTACTTATGTTG 12600

************************************************************

PMP1612_cps TTTTTTATGTTCTGTATATTTTGAGTTACAAAATAGAACTCACACTATTTAAAGTACCTC 12660

PMP1615_cps TTTTTTATGTTCTGTATATTTTGAGTTACAAAATAGAACTCACACTATTTAAAGTACCTC 12660

************************************************************

PMP1612_cps TTCAATTTTTATTTTACTTTAGTTTAGGCTTCTTGTTTGAATCAAATAGAGAAAAATATA 12720

PMP1615_cps TTCAATTTTTATTTTACTTTAGTTTAGGCTTCTTGTTTGAATCAAATAGAGAAAAATATA 12720

************************************************************

PMP1612_cps ATCAATTTATTAATAGGAAAAAAATTATATTTTGTTATTATCTACGGTATTTGTCTTAAT 12780

PMP1615_cps ATCAATTTATTAATAGGAAAAAAATTATATTTTGTTATTATCTACGGTATTTGTCTTAAT 12780

************************************************************

PMP1612_cps GGTTTTGTTAAATTTACTCAGGTATTTTTTAGTAAAGTATTAGTTGAGCTATTGGCTGTC 12840

PMP1615_cps GGTTTTGTTAAATTTACTCAGGTATTTTTTAGTAAAGTATTAGTTGAGCTATTGGCTGTC 12840

************************************************************

PMP1612_cps TTGGGTTCATTATTAACGTATAGCATCGCATATCAATTATCTCAAAAGAAAAGTGTTGGT 12900

PMP1615_cps TTGGGTTCATTATTAACGTATAGCATCGCATATCAATTATCTCAAAAGAAAAGTGTTGGT 12900

************************************************************

PMP1612_cps GATGCTAGTATTTTTAAGATGATTCTAATTAATGGATTAGGTATATATATTTTTTCTGAT 12960

PMP1615_cps GATGCTAGTATTTTTAAGATGATTCTAATTAATGGATTAGGTATATATATTTTTTCTGAT 12960

************************************************************

PMP1612_cps CCATTAAATTATTTAATCTTGAAGTTAAGTTATTCCCTAAATTCGTATTTTATGTTTACA 13020

PMP1615_cps CCATTAAATTATTTAATCTTGAAGTTAAGTTATTCCCTAAATTCGTATTTTATGTTTACA 13020

************************************************************

PMP1612_cps CCAGTAGGAATAATTGTTTTAGTAGTATTACGATTTTTTCTCACGTTATTCATATCATTG 13080

PMP1615_cps CCAGTAGGAATAATTGTTTTAGTAGTATTACGATTTTTTCTCACGTTATTCATATCATTG 13080

************************************************************

PMP1612_cps ATAGGAACAATCATATTTAAAAAAATACAAATGGCTAGTCAACTAGGTAGAGACAATAGC 13140

PMP1615_cps ATAGGAACAATCATATTTAAAAAAATACAAATGGCTAGTCAACTAGGTAGAGACAATAGC 13140

************************************************************

PMP1612_cps TATTTAAAAAGTTAAATCTATATATAATGGAGAAAAAGTAATGAAAGTACTTAAAAACTA 13200

PMP1615_cps TATTTAAAAAGTTAAATCTATATATAATGGAGAAAAAGTAATGAAAGTACTTAAAAACTA 13200

************************************************************

PMP1612_cps CGCCTACAATCTTTCTTATCAATTGTTGGTGATTTTACTCCCGATTATTACGACTCCCTA 13260

PMP1615_cps CGCCTACAATCTTTCTTATCAATTGTTGGTGATTTTACTCCCGATTATTACGACTCCCTA 13260

************************************************************

PMP1612_cps TGTAACACGGGTCTTTTCTTCGGATGATTTAGGGACGTATGGTTATTTTAATTCCATCGT 13320

PMP1615_cps TGTAACACGGGTCTTTTCTTCGGATGATTTAGGGACGTATGGTTATTTTAATTCCATCGT 13320

************************************************************

PMP1612_cps TACTTATTTTATCCTCTTAGCGACGCTAGGAGTTGCTAACTATGGGACCAAGGTCATTTC 13380

PMP1615_cps TACTTATTTTATCCTCTTAGCGACGCTAGGAGTTGCTAACTATGGGACCAAGGTCATTTC 13380

************************************************************

PMP1612_cps AGGGCATCGAAAGCAAATTCAAAAAAACTTTTTGGGAATCTATTCTCTGCAATTAGGTGC 13440

PMP1615_cps AGGGCATCGAAAGCAAATTCAAAAAAACTTTTTGGGAATCTATTCTCTGCAATTAGGTGC 13440

************************************************************

PMP1612_cps AACAGTTCTTTCTCTGTCCTTGTATGCTCTTCTTTGTCTAACTCTTCCCTTTATGCAAAA 13500

PMP1615_cps AACAGTTCTTTCTCTGTCCTTGTATGCTCTTCTTTGTCTAACTCTTCCCTTTATGCAAAA 13500

************************************************************

PMP1612_cps TCCGGTAGTCTATATTCTAGGCTTGAGTTTAGTTTCTAAAGGTTTAGACATCTCCTGGCT 13560

PMP1615_cps TCCGGTAGTCTATATTCTAGGCTTGAGTTTAGTTTCTAAAGGTTTAGACATCTCCTGGCT 13560

************************************************************

PMP1612_cps CTTTCAAGGGTTGGAGGATTTTCGAAAGATTACTGTCCGAAATATAACAGTTAAACTAGT 13620

PMP1615_cps CTTTCAAGGGTTGGAGGATTTTCGAAAGATTACTGTCCGAAATATAACAGTTAAACTAGT 13620

************************************************************

PMP1612_cps TGGGGTCATTTCCATCTTCCTCTTTGTTAAATCATCAAATGACCTTTACCTCTATGTCTT 13680

PMP1615_cps TGGGGTCATTTCCATCTTCCTCTTTGTTAAATCATCAAATGACCTTTACCTCTATGTCTT 13680

************************************************************

PMP1612_cps TTTGCTAACCATTTTTGAACTCTTGGGTCAATTCAGTATGTGGATACCAGCTCGAGAGTT 13740

PMP1615_cps TTTGCTAACCATTTTTGAACTCTTGGGTCAATTCAGTATGTGGATACCAGCTCGAGAGTT 13740

************************************************************

PMP1612_cps TATTGGTAGACCTCATTTTGACATAGAATATGCTAGACATCATTTGAAACCAGTCATATT 13800

PMP1615_cps TATTGGTAGACCTCATTTTGACATAGAATATGCTAGACATCATTTGAAACCAGTCATATT 13800

************************************************************

PMP1612_cps ATTGTTCCTTCCGCAAGTGGCGATTTCCTTGTATGTTACGCTAAATCGTACTATGCTTGG 13860

PMP1615_cps ATTGTTCCTTCCGCAAGTGGCGATTTCCTTGTATGTTACGCTAAATCGTACTATGCTTGG 13860

************************************************************

PMP1612_cps AGCGTTAGCTTCTACAAAAGATGTAGGGATTTATGACCAGGCTTTAAAGTTGGTAACTAT 13920

PMP1615_cps AGCGTTAGCTTCTACAAAAGATGTAGGGATTTATGACCAGGCTTTAAAGTTGGTAACTAT 13920

************************************************************

PMP1612_cps CCTTCTGACCTTGGTAACTTCCTTGGGAAGCGTTATGTTACCTCGAGTCGCTCATTTGTT 13980

PMP1615_cps CCTTCTGACCTTGGTAACTTCCTTGGGAAGCGTTATGTTACCTCGAGTCGCTCATTTGTT 13980

************************************************************

PMP1612_cps AGCGACAGATGATCATAAGGCAGTCAATAGGATGCATGAAATGTCTTTCTTAATTTATAA 14040

PMP1615_cps AGCGACAGATGATCATAAGGCAGTCAATAGGATGCATGAAATGTCTTTCTTAATTTATAA 14040

************************************************************

PMP1612_cps TTTAGTAATTTTTCCAATGATGGCAGGAATCTTGATTGTCAATGATGATTTTGTTCAGTT 14100

PMP1615_cps TTTAGTAATTTTTCCAATGATGGCAGGAATCTTGATTGTCAATGATGATTTTGTTCAGTT 14100

************************************************************

PMP1612_cps TTTCCTTGGTCAAGATTTTCAGGATGCGCGTTATGCAATCGCCATTATGATCTTCCGTAT 14160

PMP1615_cps TTTCCTTGGTCAAGATTTTCAGGATGCGCGTTATGCAATCGCCATTATGATCTTCCGTAT 14160

************************************************************

PMP1612_cps GTTCTTTATCGGTTGGACCAATATCATGGGAATTCAGATGCTGATACCTCATAATCAAAA 14220

PMP1615_cps GTTCTTTATCGGTTGGACCAATATCATGGGAATTCAGATGCTGATACCTCATAATCAAAA 14220

************************************************************

PMP1612_cps TAAAGAATTCATGATTTCAACAACAGCTCCCGCAATTATCAGTGTAGGTTTGAACTTACT 14280

PMP1615_cps TAAAGAATTCATGATTTCAACAACAGCTCCCGCAATTATCAGTGTAGGTTTGAACTTACT 14280

************************************************************

PMP1612_cps ATTCCTTCCTAAACTGGGATATATCGGAGCAGCCATTGTCTCTGTTTTAACAGAGGCACT 14340

PMP1615_cps ATTCCTTCCTAAACTGGGATATATCGGAGCAGCCATTGTCTCTGTTTTAACAGAGGCACT 14340

************************************************************

PMP1612_cps TGTATGGGCAATCCAATTATTCTATACTCGCAGATATTTAAAAGAAGTTCCTATAATCGG 14400

PMP1615_cps TGTATGGGCAATCCAATTATTCTATACTCGCAGATATTTAAAAGAAGTTCCTATAATCGG 14400

************************************************************

PMP1612_cps ATCAATGTCAAAAATTATACTAGCATCTGCCATTATGTATGGCCTTTTACTAAGTTCAAA 14460

PMP1615_cps ATCAATGTCAAAAATTATACTAGCATCTGCCATTATGTATGGCCTTTTACTAAGTTCAAA 14460

************************************************************

PMP1612_cps AACAGTTATACATTTTTCACCGACCTTAAATGTTCTAGCATTTGCAGCGCTTGGTGGAAT 14520

PMP1615_cps AACAGTTATACATTTTTCACCGACCTTAAATGTTCTAGCATTTGCAGCGCTTGGTGGAAT 14520

************************************************************

PMP1612_cps CATTTATCTTTTTGCAATTCTATCTCTGAAAGTGGTAGATGTGAAAGAATTAAAACAAAT 14580

PMP1615_cps CATTTATCTTTTTGCAATTCTATCTCTGAAAGTGGTAGATGTGAAAGAATTAAAACAAAT 14580

************************************************************

PMP1612_cps TATTAGGAAAAACTAAAATGAGAAAAATTCGAAATATCAACCTAGATTTACTAAAAGTGC 14640

PMP1615_cps TATTAGGAAAAACTAAAATGAGAAAAATTCGAAATATCAACCTAGATTTACTAAAAGTGC 14640

************************************************************

PMP1612_cps TTGCATGTGTTGGAGTTGTTTTACTTCATACAACAATGGGCGGATTTAAAGAGACAGGCT 14700

PMP1615_cps TTGCATGTGTTGGAGTTGTTTTACTTCATACAACAATGGGCGGATTTAAAGAGACAGGCT 14700

************************************************************

PMP1612_cps CATATAATCTTTTGGCATATTTATATTATTTAGGTACTTACTCTATTCCCCTGTTTTTTA 14760

PMP1615_cps CATATAATCTTTTGGCATATTTATATTATTTAGGTACTTACTCTATTCCCCTGTTTTTTA 14760

************************************************************

PMP1612_cps TGATCAATGGTTATTTATTGTTAGGCAAGAGGGAAATAACTTATCTTTACATACTCCAGA 14820

PMP1615_cps TGATCAATGGTTATTTATTGTTAGGCAAGAGGGAAATAACTTATCTTTACATACTCCAGA 14820

************************************************************

PMP1612_cps AAGTAAAATGGATTTTAATAACAGTGTCATCATGGACATTTATCGTATGGCTTTTTTATC 14880

PMP1615_cps AAGTAAAATGGATTTTAATAACAGTGTCATCATGGACATTTATCGTATGGCTTTTTTATC 14880

************************************************************

PMP1612_cps GTGATTTTACAACTAATCCTATTAAAAAAATTGTAGGTTCTTTGATACAAAGAGGTTATT 14940

PMP1615_cps GTGATTTTACAACTAATCCTATT-AAAAAATTGTAGGTTCTTTGATACAAAGAGGTTATT 14939

*********************** ************************************

PMP1612_cps TCTCTCAGTTTTGGTTTTTCGGTGCACTAATACTTATCTATTTATGTTTGCCAATTGTGA 15000

PMP1615_cps TCTCTCAGTTTTGGTTTTTCGGTGCACTAATACTTATCTATTTATGTTTGCCAATTGTGA 14999

************************************************************

PMP1612_cps GACAATTTCTAAATTCAAAAAGAAGCTATTTATACAGTTTATCTTTATTGATGACTATTG 15060

PMP1615_cps GACAATTTCTAAATTCAAAAAGAAGCTATTTATACAGTTTATCTTTATTGATGACTATTG 15059

************************************************************

PMP1612_cps GTTTGATTTTTGAGTTATTAAATATCCTACTTCAGATGCCAATACAAACATATGTAATAC 15120

PMP1615_cps GTTTGATTTTTGAGTTATTAAATATCCTACTTCAGATGCCAATACAAACATATGTAATAC 15119

************************************************************

PMP1612_cps AGACTTTTAGATTATGGACGTGGTTTTTTTACTATCTTTTAGGTGGTTATATAGCGCAAT 15180

PMP1615_cps AGACTTTTAGATTATGGACGTGGTTTTTTTACTATCTTTTAGGTGGTTATATAGCGCAAT 15179

************************************************************

PMP1612_cps TCACTAAAGAAGAAATCGAATCAAGATTTAAGAATTGGATGAAAATAGCTAGCATACTTT 15240

PMP1615_cps TCACTAAAGAAGAAATCGAATCAAGATTTAAGAATTGGATGAAAATAGCTAGCATACTTT 15239

************************************************************

PMP1612_cps TGTTATTGATTTCACCAATAATATTATTTTTCATAGCAAAGACCACTTACCATAATTTTT 15300

PMP1615_cps TGTTATTGATTTCACCAATAATATTATTTTTCATAGCAAAGACCACTTACCATAATTTTT 15299

************************************************************

PMP1612_cps TTGCTGAATATTTTTATGATATTTTATTAGTAAAAGTGGTAAGCGTAGGGATTTTTCTAA 15360

PMP1615_cps TTGCTGAATATTTTTATGATATTTTATTAGTAAAAGTGGTAAGCGTAGGGATTTTTCTAA 15359

************************************************************

PMP1612_cps CTATTTTCTCACTTGTATTGAATGAAAATAGCAACAAATGGATTATTTTTCTTTCTAATC 15420

PMP1615_cps CTATTTTCTCACTTGTATTGAATGAAAATAGCAACAAATGGATTATTTTTCTTTCTAATC 15419

************************************************************

PMP1612_cps AAACTATGGGTATCTTTATAATACACACTTATATTATGAAGGTATGGGAAAAACTATTTG 15480

PMP1615_cps AAACTATGGGTATCTTTATAATACACACTTATATTATGAAGGTATGGGAAAAACTATTTG 15479

************************************************************

PMP1612_cps GTTTTAGTTTTATAGGTTCATATTTACTTTTTGCTATATTTACTTTAAGTGTTAGTTTTA 15540

PMP1615_cps GTTTTAGTTTTATAGGTTCATATTTACTTTTTGCTATATTTACTTTAAGTGTTAGTTTTA 15539

************************************************************

PMP1612_cps TCATTGTTGGAATGTTAATGAAAATTCCGTATTTTAATCGAATCGTCAAATTATAAAAAG 15600

PMP1615_cps TCATTGTTGGAATGTTAATGAAAATTCCGTATTTTAATCGAATCGTCAAATTATAAAAAG 15599

************************************************************

PMP1612_cps GAGAAAAAATGTACGATTATCTTGTTGTTGGTGCTGGTCTCTTTGGTGCAGTCTTTGCCC 15660

PMP1615_cps GAGAAAAAATGTACGATTATCTTGTTGTTGGTGCTGGTCTCTTTGGTGCAGTCTTTGCCC 15659

************************************************************

PMP1612_cps ATGAAGCAGCCTTAAAAGGAAAAAAAGTAAAAGTTATTGAAAAACGAAATCATATCGCGG 15720

PMP1615_cps ATGAAGCAGCCTTAAAAGGAAAAAAAGTAAAAGTTATTGAAAAACGAAATCATATCGCGG 15719

************************************************************

PMP1612_cps GTAATATCTATACTCGTGAAGAGGAAGGAATTCAAGTTCATCAATATGGTGCTCATATCT 15780

PMP1615_cps GTAATATCTATACTCGTGAAGAGGAAGGAATTCAAGTTCATCAATATGGTGCTCATATCT 15779

************************************************************

PMP1612_cps TCCATACTTCTGATAAGGAGATCTGGGATTATGTAAATCAGTTTGCAGAGTTTAACCGTT 15840

PMP1615_cps TCCATACTTCTGATAAGGAGATCTGGGATTATGTAAATCAGTTTGCAGAGTTTAACCGTT 15839

************************************************************

PMP1612_cps ATACCAATTCTCCTGTTGCAAACTATAAGGGTGAGATTTATAACCTTCCTTTTAATATGA 15900

PMP1615_cps ATACCAATTCTCCTGTTGCAAACTATAAGGGTGAGATTTATAACCTTCCTTTTAATATGA 15899

************************************************************

PMP1612_cps ATACTTTCAATAAACTCTGGGGAGTTGTAACGCCAGCAGAAGCACAAGCTAAGATTGAGG 15960

PMP1615_cps ATACTTTCAATAAACTCTGGGGAGTTGTAACGCCAGCAGAAGCACAAGCTAAGATTGAGG 15959

************************************************************

PMP1612_cps AACAACGTGCTATTTTAAATGGTAAAACTCCTGAAAATTTGGAAGAACAGGCGATTTCTC 16020

PMP1615_cps AACAACGTGCTATTTTAAATGGTAAAACTCCTGAAAATTTGGAAGAACAGGCGATTTCTC 16019

************************************************************

PMP1612_cps TTGTAGGTACAGACATCTACGAAAAATTAATCAAAGACTATACAGAGAAACAGTGGGGCA 16080

PMP1615_cps TTGTAGGTACAGACATCTACGAAAAATTAATCAAAGACTATACAGAGAAACAGTGGGGCA 16079

************************************************************

PMP1612_cps AACCAACTACTGAACTTCCATCCTTTATTATTCGCCGTTTGCCAGTACGCCTGACCTATG 16140

PMP1615_cps AACCAACTACTGAACTTCCATCCTTTATTATTCGCCGTTTGCCAGTACGCCTGACCTATG 16139

************************************************************

PMP1612_cps ATAACAACTATTTTAACGATACCTATCAAGGGATTCCAATTGGTGGATACACTCAAATAG 16200

PMP1615_cps ATAACAACTATTTTAACGATACCTATCAAGGGATTCCAATTGGTGGATACACTCAAATAG 16199

************************************************************

PMP1612_cps TTGAAAAAATGTTGGATCATGAAAATATTGATGTAGAAACAAATGTTGATTTCTTTGTGA 16260

PMP1615_cps TTGAAAAAATGTTGGATCATGAAAATATTGATGTAGAAACAAATGTTGATTTCTTTGTGA 16259

************************************************************

PMP1612_cps ATAAAGAGCAATATCTGAAAGATTTTCCTAAGATTGTCTTTACTGGTATGATTGATGAAT 16320

PMP1615_cps ATAAAGAGCAATATCTGAAAGATTTTCCTAAGATTGTCTTTACTGGTATGATTGATGAAT 16319

************************************************************

PMP1612_cps TCTTTGACTATAAGTTGGGCGAACTAGAGTACCGTAGTCTTCGTTTTGAAAATGAGACCT 16380

PMP1615_cps TCTTTGACTATAAGTTGGGCGAACTAGAGTACCGTAGTCTTCGTTTTGAAAATGAGACCT 16379

************************************************************

PMP1612_cps TGGATATGGAAAATTACCAAGGAAATGCAGTTGTGAACTATACGGATGCAGAAACTCCAT 16440

PMP1615_cps TGGATATGGAAAATTACCAAGGAAATGCAGTTGTGAACTATACGGATGCAGAAACTCCAT 16439

************************************************************

PMP1612_cps ATACTCGCATTATTGAACACAAACATTTTGAGTTTGGGAGTCAAGCAAAGACTATCATTA 16500

PMP1615_cps ATACTCGCATTATTGAACACAAACATTTTGAGTTTGGGAGTCAAGCAAAGACTATCATTA 16499

************************************************************

PMP1612_cps CTAAAGAACATTCTAAAACATGGGAAAAAGGTGATGAGCCTTATTATCCAGTTAATAATG 16560

PMP1615_cps CTAAAGAACATTCTAAAACATGGGAAAAAGGTGATGAGCCTTATTATCCAGTTAATAATG 16559

************************************************************

PMP1612_cps ATCGTAATAATCATTTGTATAAATCATATAAAAAACTTGCTGATGAGCAAGGGAATGTTA 16620

PMP1615_cps ATCGTAATAATCATTTGTATAAATCATATAAAAAACTTGCTGATGAGCAAGGGAATGTTA 16619

************************************************************

PMP1612_cps TCTTTGGTGGCCGCTTAGGACACTATCGTTATTACGATATGCACCAAGTAATTGGAGTAG 16680

PMP1615_cps TCTTTGGTGGCCGCTTAGGACACTATCGTTATTACGATATGCACCAAGTAATTGGAGTAG 16679

************************************************************

PMP1612_cps CTTTGCAGTGCGTGAGAAATGAGTTAAATTAA 16712

PMP1615_cps CTTTGCAGTGCGTGAGAAATGAGTTAAATTAA 16711

********************************

Supplementary Figure 1: DNA sequence alignment of *cps* loci from serotype 33G (PMP1612, Genbank accession OR509582) and 33G-like (PMP1615, Genbank accession PQ281427) isolates of GPSC687. Highlighted positions denote differences.

PMP1486_cps ATGAGTAGACGTTTTAAAAAATCAGGTTCACAGAAAGTGAAGCGAAGTGTTAATATCGTT 60

PMP1623_cps ATGAGTAGACGTTTTAAAAAATCAGGTTCACAGAAAGTGAAGCGAAGTGTTAATATCGTT 60

************************************************************

PMP1486_cps TTACTGACTATTTATTTATTGTTAGTTGGTTTTTTATTGTTCTTAATCTTTAAGTACAAT 120

PMP1623_cps TTACTGACTATTTATTTATTGTTAGTTGGTTTTTTATTGTTCTTAATCTTTAAGTACAAT 120

************************************************************

PMP1486_cps ATCCTTGCTTTTAGATATCTTAACCTAGTGGTAACTGCGTTAGTCCTACTAGTTGCCTTG 180

PMP1623_cps ATCCTTGCTTTTAGATATCTTAACCTAGTGGTAACTGCGTTAGTCCTACTAGTTGCCTTG 180

************************************************************

PMP1486_cps GTAGGGCTACTCTTGGTTATCTATAAAAAAGCTGAAAAATTTACTATTTTTCTGTTGCTG 240

PMP1623_cps GTAGGGCTACTCTTGGTTATCTATAAAAAAGCTGAAAAATTTACTATTTTTCTGTTGCTG 240

************************************************************

PMP1486_cps TTCTCTATCCTTGTCAGCTCTGTGTCGCTCTTTGCAGTACAGCAGTTTGTTGGACTGACC 300

PMP1623_cps TTCTCTATCCTTGTCAGCTCTGTGTCGCTCTTTGCAGTACAGCAGTTTGTTGGACTGACC 300

************************************************************

PMP1486_cps AATCGTTTAAATGCGACTTCTAATTACTCAGAATATTCAATCAGTGTCGCTGTTTTAGCA 360

PMP1623_cps AATCGTTTAAATGCGACTTCTAATTACTCAGAATATTCAATCAGTGTCGCTGTTTTAGCA 360

************************************************************

PMP1486_cps GATAGTGATATCGAAAATGTTACGCAACTGACGAGTGTGACAGCACCGACTGGGACTGAT 420

PMP1623_cps GATAGTGATATCGAAAATGTTACGCAACTGACGAGTGTGACAGCACCGACTGGGACTGAT 420

************************************************************

PMP1486_cps AATGAAAATATTCAAAAACTACTAGCTGATATCAAGTCAAGTCAGAATACCGATTTGACG 480

PMP1623_cps AATGAAAATATTCAAAAACTACTAGCTGATATCAAGTCAAGTCAGAATACCGATTTGACG 480

************************************************************

PMP1486_cps GTCAACCAGAGTTCGTCTTACTTGGCAGCTTACAAGAGTTTGATTGCAGGGGAGACTAAG 540

PMP1623_cps GTCAACCAGAGTTCGTCTTACTTGGCAGCTTACAAGAGTTTGATTGCAGGGGAGACTAAG 540

************************************************************

PMP1486_cps GCCATTGTCCTAAATAGTGTCTTTGAAAATATCATCGAGTCAGAGTATCCAGACTACGCA 600

PMP1623_cps GCCATTGTCCTAAATAGTGTCTTTGAAAATATCATCGAGTCAGAGTATCCAGACTACGCA 600

************************************************************

PMP1486_cps TCGAAGATAAAAAAGATTTATACCAAGGGATTCACTAAAAAAGTAGAAGCTCCTAAGACG 660

PMP1623_cps TCGAAGATAAAAAAGATTTATACCAAGGGATTCACTAAAAAAGTAGAAGCTCCTAAGACG 660

************************************************************

PMP1486_cps TCTAAGAATCAGTCTTTCAATATCTATGTTAGTGGAATTGACACCTATGGCCCTATTAGT 720

PMP1623_cps TCTAAGAATCAGTCTTTCAATATCTATGTTAGTGGAATTGACACCTATGGCCCTATTAGT 720

************************************************************

PMP1486_cps TCGGTGTCGCGATCAGATGTCAATATCCTGATGACTGTCAATCGAGATACCAAGAAAATC 780

PMP1623_cps TCGGTGTCGCGATCAGATGTCAATATCCTGATGACTGTCAATCGAGATACCAAGAAAATC 780

************************************************************

PMP1486_cps CTCTTGACCACAACGCCACGTGATGCCTATGTACCAATCGCAGATGGTGGAAATAATCAA 840

PMP1623_cps CTCTTGACCACAACGCCACGTGATGCCTATGTACCAATCGCAGATGGTGGAAATAATCAA 840

************************************************************

PMP1486_cps AAAGATAAATTAACCCATGCGGGCATTTATGGAGTTGATTCGTCCATTCACACCTTAGAA 900

PMP1623_cps AAAGATAAATTAACCCATGCGGGCATTTATGGAGTTGATTCGTCCATTCACACCTTAGAA 900

************************************************************

PMP1486_cps AATCTCTATGGAGTGGATATCAATTACTATGTGCGATTGAACTTCACTTCGTTTTTGAAA 960

PMP1623_cps AATCTCTATGGAGTGGATATCAATTACTATGTGCGATTGAACTTCACTTCGTTTTTGAAA 960

************************************************************

PMP1486_cps TTGATTGATTTGTTGGGTGGAATTGATGTTTATAATGATCAAGAATTTACTGCCCATACG 1020

PMP1623_cps TTGATTGATTTGTTGGGTGGAATTGATGTTTATAATGATCAAGAATTTACTGCCCATACG 1020

************************************************************

PMP1486_cps AATGGAAAGTATTACCCTGCAGGCAATGTTCATCTTGATTCAGAACAGGCTCTCGGTTTT 1080

PMP1623_cps AATGGAAAGTATTACCCTGCAGGCAATGTTCATCTTGATTCAGAACAGGCTCTCGGTTTT 1080

************************************************************

PMP1486_cps GTTCGTGAGCGCTACTCCCTAGCAGATGGCGATCGTGACCGCGGGCGCAATCAACAAAAG 1140

PMP1623_cps GTTCGTGAGCGCTACTCCCTAGCAGATGGCGATCGTGACCGCGGGCGCAATCAACAAAAG 1140

************************************************************

PMP1486_cps GTGATTGTGGCTATCCTTCAAAAATTAACGTCAACCGAAGCACTGAAAAATTATAGTACG 1200

PMP1623_cps GTGATTGTGGCTATCCTTCAAAAATTAACGTCAACCGAAGCACTGAAAAATTATAGTACG 1200

************************************************************

PMP1486_cps ATCATTGATAGCTTGCAAGATTCTATCCAAACAAATATGCCACTTGAGACAATGATAAAT 1260

PMP1623_cps ATCATTGATAGCTTGCAAGATTCTATCCAAACAAATATGCCACTTGAGACAATGATAAAT 1260

************************************************************

PMP1486_cps TTGGTCAATGCTCAGTTGGAAAGTGGAGGGAATTATAAAGTAAATTCTCAAGATTTAAAA 1320

PMP1623_cps TTGGTCAATGCTCAGTTGGAAAGTGGAGGGAATTATAAAGTAAATTCTCAAGATTTAAAA 1320

************************************************************

PMP1486_cps GGGACAGGTCGGATGGATCTTCCTTCTTATGCAATGCCAGACAGTAACCTCTATGTGATG 1380

PMP1623_cps GGGACAGGTCGGATGGATCTTCCTTCTTATGCAATGCCAGACAGTAACCTCTATGTGATG 1380

************************************************************

PMP1486_cps GAAATAGATGATAGTAGTTTAGCTGTAGTTAAAGCAGCTATACAGGATGTGATGGAGGGT 1440

PMP1623_cps GAAATAGATGATAGTAGTTTAGCTGTAGTTAAAGCAGCTATACAGGATGTGATGGAGGGT 1440

************************************************************

PMP1486_cps AGATGAAATGATAGACATCCATTCGCACATCGTCTTTGATGTAGATGATGGTCCCAAGTC 1500

PMP1623_cps AGATGAAATGATAGACATCCATTCGCACATCGTCTTTGATGTAGATGATGGTCCCAAGTC 1500

************************************************************

PMP1486_cps AAGGGAGGAAAGCAAGACTCTCTTGGCAGAAGCCTATAGACAGGGGGTGCGAACCATTGT 1560

PMP1623_cps AAGGGAGGAAAGCAAGACTCTCTTGGCAGAAGCCTATAGACAGGGGGTGCGAACCATTGT 1560

************************************************************

PMP1486_cps CTCTACCTCTCACCGTCGCAAGGGCATGTTTGAAACTCCGGAAGAGAAGATAGCAGAAAA 1620

PMP1623_cps CTCTACCTCTCACCGTCGCAAGGGCATGTTTGAAACTCCGGAAGAGAAGATAGCAGAAAA 1620

************************************************************

PMP1486_cps CTTTCTTCAGGTTCGGGAAATAGCTAAGGAAGTGGCGAGTGACTTGGTCATTGCTTACGG 1680

PMP1623_cps CTTTCTTCAGGTTCGGGAAATAGCTAAGGAAGTGGCGAGTGACTTGGTCATTGCTTACGG 1680

************************************************************

PMP1486_cps GGCTGAAATTTACTACACACCAGATATTCTGGATAAGCTGGAAAAAAAGCGGATTCCGAC 1740

PMP1623_cps GGCTGAAATTTACTACACACCAGATATTCTGGATAAGCTGGAAAAAAAGCGGATTCCGAC 1740

************************************************************

PMP1486_cps CCTCAATGATAGTCGTTATGCCTTGATAGAGTTTAGTGTGAACACTCCTTATCGCGATAT 1800

PMP1623_cps CCTCAATGATAGTCGTTATGCCTTGATAGAGTTTAGTGTGAACACTCCTTATCGCGATAT 1800

************************************************************

PMP1486_cps TCATAGCGCCTTGAGCAAGATCTTGATGTTGGGAATTACTCCAGTCATTGCCCACATTGA 1860

PMP1623_cps TCATAGCGCCTTGAGCAAGATCTTGATGTTGGGAATTACTCCAGTCATTGCCCACATTGA 1860

************************************************************

PMP1486_cps GCGCTATGATGCTCTTGAAAATAATGAAAAACGCGTTCGAGAACTGATCGATATGGGCTG 1920

PMP1623_cps GCGCTATGATGCTCTTGAAAATAATGAAAAACGCGTTCGAGAACTGATCGATATGGGCTG 1920

************************************************************

PMP1486_cps TTACACGCAAGTAAATAGTTCACATGTCCTCAAACCCAAACTTTTTGGCGAACGTTATAA 1980

PMP1623_cps TTACACGCAAGTAAATAGTTCACATGTCCTCAAACCCAAACTTTTTGGCGAACGTTATAA 1980

************************************************************

PMP1486_cps ATTCATGAAAAAAAGAGCTCAGTATTTTTTAGAGCAGGATTTGGTTCATGTCATTGCAAG 2040

PMP1623_cps ATTCATGAAAAAAAGAGCTCAGTATTTTTTAGAGCAGGATTTGGTTCATGTCATTGCAAG 2040

************************************************************

PMP1486_cps TGATATGCACAATCTAGACGGTAGACCTCCTCATATGGCAGAAGCATATGACCTTGTTAC 2100

PMP1623_cps TGATATGCACAATCTAGACGGTAGACCTCCTCATATGGCAGAAGCATATGACCTTGTTAC 2100

************************************************************

PMP1486_cps CCAAAAATACGGAGAAGCGAAGGCTCAGGAACTTTTTATAGACAATCCTCGAAAAATTGT 2160

PMP1623_cps CCAAAAATACGGAGAAGCGAAGGCTCAGGAACTTTTTATAGACAATCCTCGAAAAATTGT 2160

************************************************************

PMP1486_cps AATGGATCAACTAATTTAGGAGAAATGATGAAAGAACAAAACACGATAGAAATCGATGTA 2220

PMP1623_cps AATGGATCAACTAATTTAGGAGAAATGATGAAAGAACAAAACACGATAGAAATCGATGTA 2220

************************************************************

PMP1486_cps TTTCAATTAGTTAAAACCTTGTGGAAACGCAAGCTAATGATTTTAATAGTGGCACTTGTG 2280

PMP1623_cps TTTCAATTAGTTAAAACCTTGTGGAAACGCAAGCTAATGATTTTAATAGTGGCACTTGTG 2280

************************************************************

PMP1486_cps ACAGGTGCGGGGGCTTTTGCATATAGCACTTTTATTGTTAAGCCAGAATATACGAGTACC 2340

PMP1623_cps ACAGGTGCGGGGGCTTTTGCATATAGCACTTTTATTGTTAAGCCAGAATATACGAGTACC 2340

************************************************************

PMP1486_cps ACGCGAATTTACGTAGTGAATCGCAATCAAGGAGACAAGCCGGGGCTGACAAATCAGGAT 2400

PMP1623_cps ACGCGAATTTACGTAGTGAATCGCAATCAAGGAGACAAGCCGGGGCTGACAAATCAGGAT 2400

************************************************************

PMP1486_cps TTGCAGGCAGGAACTTATCTGGTAAAAGACTACCGTGAGATTATCCTTTCGCAGGATGCA 2460

PMP1623_cps TTGCAGGCAGGAACTTATCTGGTAAAAGACTACCGTGAGATTATCCTTTCGCAGGATGCA 2460

************************************************************

PMP1486_cps TTGGAAAAAGTAGCGACAAATTTGAAGTTGGATATGTCAGCAAAAACGTTAGCCAGCAAA 2520

PMP1623_cps TTGGAAAAAGTAGCGACAAATTTGAAGTTGGATATGTCAGCAAAAACGTTAGCCAGCAAA 2520

************************************************************

PMP1486_cps GTTCAAGTGGCTGTACCAGCTGACACTCGTATCGTCTCAATCTCTGTCAAGGATAAACAG 2580

PMP1623_cps GTTCAAGTGGCTGTACCAGCTGACACTCGTATCGTCTCAATCTCTGTCAAGGATAAACAG 2580

************************************************************

PMP1486_cps CCAGAGGAAGCCAGTCGTATCGCTAATTCTCTACGAGAAGTTGCTGCAGAAAAGATCGTC 2640

PMP1623_cps CCAGAGGAAGCCAGTCGTATCGCTAATTCTCTACGAGAAGTTGCTGCAGAAAAGATCGTC 2640

************************************************************

PMP1486_cps GCTGTAACGCGAGTATCTGATGTAACGACACTTGAAGAAGCGCGACCAGCTACGACTCCC 2700

PMP1623_cps GCTGTAACGCGAGTATCTGATGTAACGACACTTGAAGAAGCGCGACCAGCTACGACTCCC 2700

************************************************************

PMP1486_cps TCTTCTCCAAATGTTCGACGCAATTCCTTGTTTGGTTTTCTTGGAGGAGCAGTCGTAACA 2760

PMP1623_cps TCTTCTCCAAATGTTCGACGCAATTCCTTGTTTGGTTTTCTTGGAGGAGCAGTCGTAACA 2760

************************************************************

PMP1486_cps GTAATTGCTGTTCTTTTGATTGAGTTGCTCGACACCCGTGTGAAACGTCCTGAAGATGTT 2820

PMP1623_cps GTAATTGCTGTTCTTTTGATTGAGTTGCTCGACACCCGTGTGAAACGTCCTGAAGATGTT 2820

************************************************************

PMP1486_cps GAAGATGTACTGCAAATTCCACTTTTAGGGCTCGTTCCAGATTTGGACAAAATGAAATAG 2880

PMP1623_cps GAAGATGTACTGCAAATTCCACTTTTAGGGCTCGTTCCAGATTTGGACAAAATGAAATAG 2880

************************************************************

PMP1486_cps GAGGAAGTTATGCCAACGTTAGAAATCTCACAGGCAAAATTGGATTTTGTAAAAAAGGCA 2940

PMP1623_cps GAGGAAGTTATGCCAACGTTAGAAATCTCACAGGCAAAATTGGATTTTGTAAAAAAGGCA 2940

************************************************************

PMP1486_cps GAGGAATATTATAACGCTTTGTGCACGAACCTACAGTTAAGTGGAGATGGTTTGAAAGTA 3000

PMP1623_cps GAGGAATATTATAACGCTTTGTGCACGAACCTACAGTTAAGTGGAGATGGTTTGAAAGTA 3000

************************************************************

PMP1486_cps TTTTCTATCACTTCTGTGAAAATAGGAGAAGGAAAATCAACGACTTCTACCAATATCGCT 3060

PMP1623_cps TTTTCTATCACTTCTGTGAAAATAGGAGAAGGAAAATCAACGACTTCTACCAATATCGCT 3060

************************************************************

PMP1486_cps TGGGCTTTTGCGCGTGCAGGTTACAAAACGCTGCTGATTGATGGAGATATTCGCAATTCT 3120

PMP1623_cps TGGGCTTTTGCGCGTGCAGGTTACAAAACGCTGCTGATTGATGGAGATATTCGCAATTCT 3120

************************************************************

PMP1486_cps GTTATGTTAGGTGTCTTTAAAGCAAGGGATAAGATTACGGGCCTGACAGAATTTTTATCA 3180

PMP1623_cps GTTATGTTAGGTGTCTTTAAAGCAAGGGATAAGATTACGGGCCTGACAGAATTTTTATCA 3180

************************************************************

PMP1486_cps GGAACTACAGACCTATCACAAGGGCTTTGTGATACCAATATCGAAAATCTCTTTGTAATT 3240

PMP1623_cps GGAACTACAGACCTATCACAAGGGCTTTGTGATACCAATATCGAAAATCTCTTTGTAATT 3240

************************************************************

PMP1486_cps CAGGCTGGCTCTGTGTCACCGAATCCGACAGCTCTTCTTCAAAGTAAGAATTTCAGTACA 3300

PMP1623_cps CAGGCTGGCTCTGTGTCACCGAATCCGACAGCTCTTCTTCAAAGTAAGAATTTCAGTACA 3300

************************************************************

PMP1486_cps ATGCTTGAAACCTTGCGTAAATATTTTGACTACATCATTGTAGATACTGCTCCTGTCGGT 3360

PMP1623_cps ATGCTTGAAACCTTGCGTAAATATTTTGACTACATCATTGTAGATACTGCTCCTGTCGGT 3360

************************************************************

PMP1486_cps GTCGTGATTGATGCTGCTATTATTACGCGAAAATGCGATGCTTCTATTTTAGTGACGGAG 3420

PMP1623_cps GTCGTGATTGATGCTGCTATTATTACGCGAAAATGCGATGCTTCTATTTTAGTGACGGAG 3420

************************************************************

PMP1486_cps GCAGGTGAAATAAATCGACGGGATATTCAAAAAGCAAAAGAACAGTTAGAACACACAGGG 3480

PMP1623_cps GCAGGTGAAATAAATCGACGGGATATTCAAAAAGCAAAAGAACAGTTAGAACACACAGGG 3480

************************************************************

PMP1486_cps AAGCCGTTTTTGGGAGTTGTGTTGAATAAATTCGATACTTCAGTAGACAAATACGGTTCT 3540

PMP1623_cps AAGCCGTTTTTGGGAGTTGTGTTGAATAAATTCGATACTTCAGTAGACAAATACGGTTCT 3540

************************************************************

PMP1486_cps TATGGAAATTATGGAGATTACGGGAAAAATAAAAAATAGGTCGGGGGATAGAGATGAATG 3600

PMP1623_cps TATGGAAATTATGGAGATTACGGGAAAAATAAAAAATAGGTCGGGGGATAGAGATGAATG 3600

************************************************************

PMP1486_cps GAAAAATAGTAAAGTCTTCATTGGCTATAATCCAGAGTTTTCTTGTTATTTTATTGACTT 3660

PMP1623_cps GAAAAATAGTAAAGTCTTCATTGGCTATAATCCAGAGTTTTCTTGTTATTTTATTGACTT 3660

************************************************************

PMP1486_cps ATCTACTTAGTGCTGTGAGAGAAGTGGAGATTGTTTCAACAACAGCTATTGCACTTTATA 3720

PMP1623_cps ATCTACTTAGTGCTGTGAGAGAAGTGGAGATTGTTTCAACAACAGCTATTGCACTTTATA 3720

************************************************************

PMP1486_cps TCCTCCATTATTTTGTCTTTTATATCAGTGATTATGGACAGGATTTCTTTAAAAGGGGAT 3780

PMP1623_cps TCCTCCATTATTTTGTCTTTTATATCAGTGATTATGGACAGGATTTCTTTAAAAGGGGAT 3780

************************************************************

PMP1486_cps ATTTGATTGAACTTGTCCAGACATTGAAATATATCCTATTCTTTGCACTAGCGATTAATA 3840

PMP1623_cps ATTTGATTGAACTTGTCCAGACATTGAAATATATCCTATTCTTTGCACTAGCGATTAATA 3840

************************************************************

PMP1486_cps TTTCTAATTTTTTCTTAGAGGATCGATTTAGTATTTCCAGACGAGGCATGATTTACTTCC 3900

PMP1623_cps TTTCTAATTTTTTCTTAGAGGATCGATTTAGTATTTCCAGACGAGGCATGATTTACTTCC 3900

************************************************************

PMP1486_cps TCACATTACATGCTCTCTTAGTCTATGTGCTAAACCTATTTATCAAGTGGTATTGGAAGC 3960

PMP1623_cps TCACATTACATGCTCTCTTAGTCTATGTGCTAAACCTATTTATCAAGTGGTATTGGAAGC 3960

************************************************************

PMP1486_cps GGGCTTATCCCAACTTTAAAGGAAGTAAGAAGATTCTCCTACTTACAGCAACTTCTCGTG 4020

PMP1623_cps GGGCTTATCCCAACTTTAAAGGAAGTAAGAAGATTCTCCTACTTACAGCAACTTCTCGTG 4020

************************************************************

PMP1486_cps TCGAAAAGGTACTGGATAGATTAATAGAATCAAATGAGGTTGTTGGGGAGTTGGTAGCCG 4080

PMP1623_cps TCGAAAAGGTACTGGATAGATTAATAGAATCAAATGAGGTTGTTGGGGAGTTGGTAGCCG 4080

************************************************************

PMP1486_cps TCAGTGTCTTAGATAAACCAGATTTTCAGCATGATTATTTAAAGGTAGTAGCAGAGAGGG 4140

PMP1623_cps TCAGTGTCTTAGATAAACCAGATTTTCAGCATGATTATTTAAAGGTAGTAGCAGAGAGGG 4140

************************************************************

PMP1486_cps AGATAGTAAACTTTGCGACTTATGAGGTGGTCGATGAAGTCTTTATCAATCTTCCAAGTG 4200

PMP1623_cps AGATAGTAAACTTTGCGACTTATGAGGTGGTCGATGAAGTCTTTATCAATCTTCCAAGTG 4200

************************************************************

PMP1486_cps AAAAATACAATATTGGAGAGCTTGTTTCTCAGTTTGAAACGATGGGAATTGATGTAACAG 4260

PMP1623_cps AAAAATACAATATTGGAGAGCTTGTTTCTCAGTTTGAAACGATGGGAATTGATGTAACAG 4260

************************************************************

PMP1486_cps TTAATCTAAATGCTTTTGATCGTAGTTTGGCACGTAACAAGCAAATTCGTAAGATGGCAG 4320

PMP1623_cps TTAATCTAAATGCTTTTGATCGTAGTTTGGCACGTAACAAGCAAATTCGTAAGATGGCAG 4320

************************************************************

PMP1486_cps GATTAAACGTTGTGACTTTTTCTACAACATTTTATAAGACTAGTCATGTAATTGCTAAGC 4380

PMP1623_cps GATTAAACGTTGTGACTTTTTCTACAACATTTTATAAGACTAGTCATGTAATTGCTAAGC 4380

************************************************************

PMP1486_cps GGATTATTGATATCATGGGTGCATTGGTAGGGCTGATACTATGTGGTTTAGTCAGTATTG 4440

PMP1623_cps GGATTATTGATATCATGGGTGCATTGGTAGGGCTGATACTATGTGGTTTAGTCAGTATTG 4440

************************************************************

PMP1486_cps TACTGGTTCCTTTGATTCGAAAGGATGGGGGCTCTGCTATTTTTGCTCAGATGCGTATAG 4500

PMP1623_cps TACTGGTTCCTTTGATTCGAAAGGATGGGGGCTCTGCTATTTTTGCTCAGATGCGTATAG 4500

************************************************************

PMP1486_cps GAAAAAATGGTCGTCAGTTCACTTTTTATAAGTTTCGCTCTATGTGTGTAGATGCCGAGG 4560

PMP1623_cps GAAAAAATGGTCGTCAGTTCACTTTTTATAAGTTTCGCTCTATGTGTGTAGATGCCGAGG 4560

************************************************************

PMP1486_cps CGAAAAAAAGAGAACTCATGGAACAAAATACTATGCAGGGTGGAATGTTTAAGGTGGACG 4620

PMP1623_cps CGAAAAAAAGAGAACTCATGGAACAAAATACTATGCAGGGTGGAATGTTTAAGGTGGACG 4620

************************************************************

PMP1486_cps ATGATCCTCGTATCACGAAAATTGGTCGTTTTATACGGAAGACTAGCTTGGACGAGCTAC 4680

PMP1623_cps ATGATCCTCGTATCACGAAAATTGGTCGTTTTATACGGAAGACTAGCTTGGACGAGCTAC 4680

************************************************************

PMP1486_cps CACAGTTTTATAATGTTCTAAAGGGAGATATGAGTTTGGTTGGCACACGGCCACCAACAG 4740

PMP1623_cps CACAGTTTTATAATGTTCTAAAGGGAGATATGAGTTTGGTTGGCACACGGCCACCAACAG 4740

************************************************************

PMP1486_cps TGGACGAGTATGAACACTATACCCCAGAACAAAAACGTCGGCTAAGTTTTAAACCTGGTA 4800

PMP1623_cps TGGACGAGTATGAACACTATACCCCAGAACAAAAACGTCGGCTAAGTTTTAAACCTGGTA 4800

************************************************************

PMP1486_cps TAACAGGCTTATGGCAGATCAGTGGACGAAGTGAGATTAAGAATTTTGATGAAGTTGTCA 4860

PMP1623_cps TAACAGGCTTATGGCAGATCAGTGGACGAAGTGAGATTAAGAATTTTGATGAAGTTGTCA 4860

************************************************************

PMP1486_cps AATTAGATGTAGCCTATATAGACGATTGGACAATTTGGAAAGATATTGAGATTTTATTGA 4920

PMP1623_cps AATTAGATGTAGCCTATATAGACGATTGGACAATTTGGAAAGATATTGAGATTTTATTGA 4920

************************************************************

PMP1486_cps AGACAGTTAAAGTTGTATTGATGAAGGATGGAGCGAAGTAGATTGATAGATGTAAAAATC 4980

PMP1623_cps AGACAGTTAAAGTTGTATTGATGAAGGATGGAGCGAAGTAGATTGATAGATGTAAAAATC 4980

************************************************************

PMP1486_cps ATTGTGGCAACGCATAAAGAGGTTAAAATGCCTCAAGACAATAGTCTTTACCTTCCAATA 5040

PMP1623_cps ATTGTGGCAACGCATAAAGAGGTTAAAATGCCTCAAGACAATAGTCTTTACCTTCCAATA 5040

************************************************************

PMP1486_cps CATGTTGGGAGAGACGGTAAATCAGATATTGGTTTTATCGGTGATAATACTGGCGATAAT 5100

PMP1623_cps CATGTTGGGAGAGACGGTAAATCAGATATTGGTTTTATCGGTGATAATACTGGCGATAAT 5100

************************************************************

PMP1486_cps ATATCCTCTCTAAATCCATATTATTGTGAGTTGACGGGACTTTATTGGGCATGGAAGAAT 5160

PMP1623_cps ATATCCTCTCTAAATCCATATTATTGTGAGTTGACGGGACTTTATTGGGCATGGAAGAAT 5160

************************************************************

PMP1486_cps CTTGATTATAATTACTTAGGTTTAGTTCATTACCGTCGTTATTTTACAAATAAATCTCAA 5220

PMP1623_cps CTTGATTATAATTACTTAGGTTTAGTTCATTACCGTCGTTATTTTACAAATAAATCTCAA 5220

************************************************************

PMP1486_cps GGGTATAATGAAAATGTCAATATGGATGACGTCATTTTGTCTCGATCTAATGTTGAAATA 5280

PMP1623_cps GGGTATAATGAAAATGTCAATATGGATGACGTCATTTTGTCTCGATCTAATGTTGAAATA 5280

************************************************************

PMP1486_cps TTATTAGAGAAATCTGACATAATAGTTCCAAAGAAGCGAAAGTATTATATTGAAACTCTT 5340

PMP1623_cps TTATTAGAGAAATCTGACATAATAGTTCCAAAGAAGCGAAAGTATTATATTGAAACTCTT 5340

************************************************************

PMP1486_cps TATTCACATTATGCCCATACCCTTAACGGAGAACATCTGGATCTTGCTAGGAAAATTATT 5400

PMP1623_cps TATTCACATTATGCCCATACCCTTAACGGAGAACATCTGGATCTTGCTAGGAAAATTATT 5400

************************************************************

PMP1486_cps GAGCAAAATAGTTCAGAGTATCTTTCATCCTTTGATAAAGTGATGAAACAAAGAAGCGGT 5460

PMP1623_cps GAGCAAAATAGTTCAGAGTATCTTTCATCCTTTGATAAAGTGATGAAACAAAGAAGCGGT 5460

************************************************************

PMP1486_cps TATATGTTCAATATGTTTATCATGAAAAAAGAACTATTAGATGATTATTTACCGTGGCTT 5520

PMP1623_cps TATATGTTCAATATGTTTATCATGAAAAAAGAACTATTAGATGATTATTTACCGTGGCTT 5520

************************************************************

PMP1486_cps TTTTCTATTCTGGATACTATGTACGAACAGATGGACTTGACCGACTATACTCCATTTGAG 5580

PMP1623_cps TTTTCTATTCTGGATACTATGTACGAACAGATGGACTTGACCGACTATACTCCATTTGAG 5580

************************************************************

PMP1486_cps TCACGTTTATTCGGGCGAGTTAGTGAGTTGTTGTTTAATGTTTGGTTATGTAAACAAGGA 5640

PMP1623_cps TCACGTTTATTCGGGCGAGTTAGTGAGTTGTTGTTTAATGTTTGGTTATGTAAACAAGGA 5640

************************************************************

PMP1486_cps ATAACGCCTAAAGAGGTACCATTTATGTACATGGAGAGAGTGGATTTGTTTGAAAAAGGA 5700

PMP1623_cps ATAACGCCTAAAGAGGTACCATTTATGTACATGGAGAGAGTGGATTTGTTTGAAAAAGGA 5700

************************************************************

PMP1486_cps AAATCTTTTTTAATGGCTAAATTTTTTGGAAAGAAGTATGGACAGAGTTTTTAAATTATG 5760

PMP1623_cps AAATCTTTTTTAATGGCTAAATTTTTTGGAAAGAAGTATGGACAGAGTTTTTAAATTATG 5760

************************************************************

PMP1486_cps GTTTTGTTATTACTTACTTTTGCATTCTTTTTAGTTTTCTCTGTGCTGTCAATAAGTTTA 5820

PMP1623_cps GTTTTGTTATTACTTACTTTTGCATTCTTTTTAGTTTTCTCTGTGCTGTCAATAAGTTTA 5820

************************************************************

PMP1486_cps AGTGTTATAGGGCTAGTAAATGACAAAAAGCGTTCAAAAATCTATTTACTACTAATTAGT 5880

PMP1623_cps AGTGTTATAGGGCTAGTAAATGACAAAAAGCGTTCAAAAATCTATTTACTACTAATTAGT 5880

************************************************************

PMP1486_cps TTTGCAGTATCAATTGTTGCGCTGCGTTATATTCCTCATCATATGGCTGATGGCGCTTTT 5940

PMP1623_cps TTTGCAGTATCAATTGTTGCGCTGCGTTATATTCCTCATCATATGGCTGATGGCGCTTTT 5940

************************************************************

PMP1486_cps CATTTTCGTGCGACTACGGCCCTTATTCGCTATGATAGTATTTTCGAAATGTTTTAAGAA 6000

PMP1623_cps CATTTTCGTGCGACTACGGCCCTTATTCGCTATGATAGTATTTTCGAAATGTTTTAAGAA 6000

************************************************************

PMP1486_cps TTTTCTAGTGGATGGAATGTGGGAAGATATGATTATGGTTCTATGCCAGTTTTTACTTCA 6060

PMP1623_cps TTTTCTAGTGGATGGAATGTGGGAAGATATGATTATGGTTCTATGCCAGTTTTTACTTCA 6060

************************************************************

PMP1486_cps TTAATGTATCTTATTCGAAACACTCATCACTATAGTTTACTGAGTTTTATTTCAGCTTTT 6120

PMP1623_cps TTAATGTATCTTATTCGAAACACTCATCACTATAGTTTACTGAGTTTTATTTCAGCTTTT 6120

************************************************************

PMP1486_cps ATAACTTACTTTAGTTTTGGTTACGTAGTCGTTGATCTATTTAAAGACTTGGACAAGGTT 6180

PMP1623_cps ATAACTTACTTTAGTTTTGGTTACGTAGTCGTTGATCTATTTAAAGACTTGGACAAGGTT 6180

************************************************************

PMP1486_cps TCTAAACTATCGTATGCTACAGTATTAATTGCTGTACTATGTTTAAATAATTATAGATAT 6240

PMP1623_cps TCTAAACTATCGTATGCTACAGTATTAATTGCTGTACTATGTTTAAATAATTATAGATAT 6240

************************************************************

PMP1486_cps ACAACTGGTGGAATGAGATTTTGTATTGTAGTTGCCTTGATGATGCTTTTATTGATTTTA 6300

PMP1623_cps ACAACTGGTGGAATGAGATTTTGTATTGTAGTTGCCTTGATGATGCTTTTATTGATTTTA 6300

************************************************************

PMP1486_cps GTAAGACGGACTTACTCATAAAAAATATTTTTCAATTCTTTTCAAGTTTGCCATTTACAT 6360

PMP1623_cps GTAAGACGGACTTACTCATAAAAAATATTTTTCAATTCTTTTCAAGTTTGCCATTTACAT 6360

************************************************************

PMP1486_cps AATAAGACTATGTGCTTTTATTTTATTTTTTGGAGGATTATATGGGACTATCTACCGTGA 6420

PMP1623_cps AATAAGACTATGTGCTTTTATTTTATTTTTTGGAGGATTATATGGGACTATCTACCGTGA 6420

************************************************************

PMP1486_cps CATTGTTTAAAAATTTAAAGTTCTCAGATAGTAAATTTATTAAATTGGAAGGAGAGCTCC 6480

PMP1623_cps CATTGTTTAAAAATTTAAAGTTCTCAGATAGTAAATTTATTAAATTGGAAGGAGAGCTCC 6480

************************************************************

PMP1486_cps TTCTTAAATATCAAGAGTATTTATTAAAAATCATGGAAGATATTGTAACAGTGTGTGAGG 6540

PMP1623_cps TTCTTAAATATCAAGAGTATTTATTAAAAATCATGGAAGATATTGTAACAGTGTGTGAGG 6540

************************************************************

PMP1486_cps AAGAAGGGTTATATTATTCACTTTCTGGTGGGAGTGCATTAGGGGCTTACCGACACAAAG 6600

PMP1623_cps AAGAAGGGTTATATTATTCACTTTCTGGTGGGAGTGCATTAGGGGCTTACCGACACAAAG 6600

************************************************************

PMP1486_cps GGTTTATTCCATGGGATGATGATATGGACATATTTATGCTAGGAAGTGAGCGGGAGATTT 6660

PMP1623_cps GGTTTATTCCATGGGATGATGATATGGACATATTTATGCTAGGAAGTGAGCGGGAGATTT 6660

************************************************************

PMP1486_cps TCTTTCAAAAATTTTCTCAGAAATTTTCTGATAAATATTGGATACATAATTCACAAACAC 6720

PMP1623_cps TCTTTCAAAAATTTTCTCAGAAATTTTCTGATAAATATTGGATACATAATTCACAAACAC 6720

************************************************************

PMP1486_cps CAAACTATGGCATGCCCATTGGTCGTATCAGACAAAAAGGAACAGTTTTACGTGGTCGAG 6780

PMP1623_cps CAAACTATGGCATGCCCATTGGTCGTATCAGACAAAAAGGAACAGTTTTACGTGGTCGAG 6780

************************************************************

PMP1486_cps AGGATGTTGGAGTTGAAGAATGTGGATTTTTTATTGATATTTTTTGGCTTGAAAATGTTC 6840

PMP1623_cps AGGATGTTGGAGTTGAAGAATGTGGATTTTTTATTGATATTTTTTGGCTTGAAAATGTTC 6840

************************************************************

PMP1486_cps CTAATTCAAAAATATTGAGACAACTCCACGGTTTTCTCTGCATGGCGATTGGATTGCTAC 6900

PMP1623_cps CTAATTCAAAAATATTGAGACAACTCCACGGTTTTCTCTGCATGGCGATTGGATTGCTAC 6900

************************************************************

PMP1486_cps TATCATGTAGAAATTTTTATAAAAATCGTCAGCTGATGCTGGAGATTATGAAGGAACATA 6960

PMP1623_cps TATCATGTAGAAATTTTTATAAAAATCGTCAGCTGATGCTGGAGATTATGAAGGAACATA 6960

************************************************************

PMP1486_cps AAGAAGTAAGGCTTGTTTTTCGAATAAAACTAGTTTTAGGGTTTCTAATTAGTTTTATAT 7020

PMP1623_cps AAGAAGTAAGGCTTGTTTTTCGAATAAAACTAGTTTTAGGGTTTCTAATTAGTTTTATAT 7020

************************************************************

PMP1486_cps CTTTGAGACAATTTACAAGACTTACGGAAAGGATCTACTCATTGTGCAAGAATAATGAAT 7080

PMP1623_cps CTTTGAGACAATTTACAAGACTTACGGAAAGGATCTACTCATTGTGCAAGAATAATGAAT 7080

************************************************************

PMP1486_cps CAAGATACCTTAGTGTTCCTTCAGGAAGAAAACATTATTTTGGCGAGATGTTTATAAGAG 7140

PMP1623_cps CAAGATACCTTAGTGTTCCTTCAGGAAGAAAACATTATTTTGGCGAGATGTTTATAAGAG 7140

************************************************************

PMP1486_cps AAGATATGCAGTTAACTAGGAAACTCAATTTTGAAGGACATAAGTGGAATGTTCCAAATA 7200

PMP1623_cps AAGATATGCAGTTAACTAGGAAACTCAATTTTGAAGGACATAAGTGGAATGTTCCAAATA 7200

************************************************************

PMP1486_cps ATATTGAGCATTATTTAACTGTAATGTACGGTGATTATATGAAAATACCTGCAGTTGAAG 7260

PMP1623_cps ATATTGAGCATTATTTAACTGTAATGTACGGTGATTATATGAAAATACCTGCAGTTGAAG 7260

************************************************************

PMP1486_cps ATAGGGAATCACATATCATTTTAGAAATCTCCTTCCCTAACGAGTAAACAGTACTTTCAT 7320

PMP1623_cps ATAGGGAATCACATATCATTTTAGAAATCTCCTTCCCTAACGAGTAAACAGTACTTTCAT 7320

************************************************************

PMP1486_cps TTTTTATATGTAAGGAATTAATTTAATGAAAAAAATAGCAATTGTTAGATATAATCTAAG 7380

PMP1623_cps TTTTTATATGTAAGGAATTAATTTAATGAAAAAAATAGCAATTGTTAGATATAATCTAAG 7380

************************************************************

PMP1486_cps TAAAATTGGTGGAGCAGAAAAAGTAGCTATTAATATGGCTAATGAATTGTCACAATACTA 7440

PMP1623_cps TAAAATTGGTGGAGCAGAAAAAGTAGCTATTAATATGGCTAATGAATTGTCACAATACTA 7440

************************************************************

PMP1486_cps TGATGTAAAACTGTTATCTATTCTATTGGATGAGGATGGCTTTATAAATTATGATATTAA 7500

PMP1623_cps TGATGTAAAACTGTTATCTATTCTATTGGATGAGGATGGCTTTATAAATTATGATATTAA 7500

************************************************************

PMP1486_cps CCCTAATGTGACATTAATAAATTTTCACAAGGGTGATCTTAGAATTAGAACTGCTACATT 7560

PMP1623_cps CCCTAATGTGACATTAATAAATTTTCACAAGGGTGATCTTAGAATTAGAACTGCTACATT 7560

************************************************************

PMP1486_cps AAAATTAACAGGTAAATTAAGGAACTATATTAAAAGAGAAAAAATTGAAGTGATTTTTTC 7620

PMP1623_cps AAAATTAACAGGTAAATTAAGGAACTATATTAAAAGAGAAAAAATTGAAGTGATTTTTTC 7620

************************************************************

PMP1486_cps TATAACCCCATTAACAAATACTATGGTTAGATTAGCTACTCTAGGATTAAATGTGAAAAT 7680

PMP1623_cps TATAACCCCATTAACAAATACTATGGTTAGATTAGCTACTCTAGGATTAAATGTGAAAAT 7680

************************************************************

PMP1486_cps AGTTTTCTGTGATCATCATAGCTTAGAATTTCGTGATTTTAGAGGTAGGGAAGTGCAGAG 7740

PMP1623_cps AGTTTTCTGTGATCATCATAGCTTAGAATTTCGTGATTTTAGAGGTAGGGAAGTGCAGAG 7740

************************************************************

PMP1486_cps ATTTGTAGGAGCTAAATTTTTTGACAAGATTGTCACTTTAACAGAAGAAGATAGAATAAA 7800

PMP1623_cps ATTTGTAGGAGCTAAATTTTTTGACAAGATTGTCACTTTAACAGAAGAAGATAGAATAAA 7800

************************************************************

PMP1486_cps ATACTCCGATAAATATAATATACCTATAAATAAAGTAAATGCTATTTATAATTGGATTGA 7860

PMP1623_cps ATACTCCGATAAATATAATATACCTATAAATAAAGTAAATGCTATTTATAATTGGATTGA 7860

************************************************************

PMP1486_cps TGAAGAAGATTCTGAAAATACCCCATTTGATAATGAGACAAATAAAATAATAACGGTAGG 7920

PMP1623_cps TGAAGAAGATTCTGAAAATACCCCATTTGATAATGAGACAAATAAAATAATAACGGTAGG 7920

************************************************************

PMP1486_cps TCGATTTCACAGTCAAAAGGGATATGATTATCTTGCCGAAGTAGCTATAAAAGTATTATC 7980

PMP1623_cps TCGATTTCACAGTCAAAAGGGATATGATTATCTTGCCGAAGTAGCTATAAAAGTATTATC 7980

************************************************************

PMP1486_cps ACAACATCCAGACTGGCAGTGGGATATATATGGTTCAGGGGATAAACTTATTGAACAGGA 8040

PMP1623_cps ACAACATCCAGACTGGCAGTGGGATATATATGGTTCAGGGGATAAACTTATTGAACAGGA 8040

************************************************************

PMP1486_cps TCTAAAAAGAAAACTAGAAGAAGGTTGTGTTTCTTCACAAGTTAATTTTAAAGGGAATGT 8100

PMP1623_cps TCTAAAAAGAAAACTAGAAGAAGGTTGTGTTTCTTCACAAGTTAATTTTAAAGGGAATGT 8100

************************************************************

PMP1486_cps AAAGGGAACTGAAAATATTTATCCCAATCATAGTATCTATGTCATGACTTCTCGCCATGA 8160

PMP1623_cps AAAGGGAACTGAAAATATTTATCCCAATCATAGTATCTATGTCATGACTTCTCGCCATGA 8160

************************************************************

PMP1486_cps GGGCTTACCTTTAGTCCTATTAGAAGCACAACAATACAATCTTCCTATTGTTAGCTTCAG 8220

PMP1623_cps GGGCTTACCTTTAGTCCTATTAGAAGCACAACAATACAATCTTCCTATTGTTAGCTTCAG 8220

************************************************************

PMP1486_cps ATGTCCAACGGGACCTAGTGAGATTGTTGAAGATGGAGTCAATGGATTTTTGATTGATTG 8280

PMP1623_cps ATGTCCAACGGGACCTAGTGAGATTGTTGAAGATGGAGTCAATGGATTTTTGATTGATTG 8280

************************************************************

PMP1486_cps CTATGACGTGTATCAGATGAGTGAGAAATTGCTTGAATTGATGAAAAATGATGATTTGCG 8340

PMP1623_cps CTATGACGTGTATCAGATGAGTGAGAAATTGCTTGAATTGATGAAAAATGATGATTTGCG 8340

************************************************************

PMP1486_cps ACAATCTTTCTCAGAACATGCCAAAGACAATATAGATAAATTTGATAAAAATGAAATTCT 8400

PMP1623_cps ACAATCTTTCTCAGAACATGCCAAAGACAATATAGATAAATTTGATAAAAATGAAATTCT 8400

************************************************************

PMP1486_cps TAATCAGTGGATAGAATTGATTGATACAATTTAGGAGGTAAGATGCAAGAGTGTTTATTG 8460

PMP1623_cps TAATCAGTGGATAGAATTGATTGATACAATTTAGGAGGTAAGATGCAAGAGTGTTTATTG 8460

************************************************************

PMP1486_cps ACAATTATAATGCCTAGTTATAATATTCAGGACTATATTTCCAAAGGAATCGAGTCATTC 8520

PMP1623_cps ACAATTATAATGCCTAGTTATAATATTCAGGACTATATTTCCAAAGGAATCGAGTCATTC 8520

************************************************************

PMP1486_cps CAGCAAGTACACCTAGATTACAAACAAAAATTTGAGATCTTAATTGTGAATGATGGAAGT 8580

PMP1623_cps CAGCAAGTACACCTAGATTACAAACAAAAATTTGAGATCTTAATTGTGAATGATGGAAGT 8580

************************************************************

PMP1486_cps ACGGACGACACAGCCAAAGTAGCAGAAGAGACCCTAAGAAAAGATTTATTGCTGAATGGT 8640

PMP1623_cps ACGGACGACACAGCCAAAGTAGCAGAAGAGACCCTAAGAAAAGATTTATTGCTGAATGGT 8640

************************************************************

PMP1486_cps CGTATTATCACAAAGGAAAATGGAGGCCATGGCTCAACAATCAATCGTGGTATCCAGGAA 8700

PMP1623_cps CGTATTATCACAAAGGAAAATGGAGGCCATGGCTCAACAATCAATCGTGGTATCCAGGAA 8700

************************************************************

PMP1486_cps GCAAAGGGAAAATTCTTTAAAGTTATTGATGGGGATGACTGGGTTATTCCATCAGAATTT 8760

PMP1623_cps GCAAAGGGAAAATTCTTTAAAGTTATTGATGGGGATGACTGGGTTATTCCATCAGAATTT 8760

************************************************************

PMP1486_cps GAAAAGTTTTTAGATGACCTTACAGTTACTGAAGTGGATATGATCTTGACAGATTTTACA 8820

PMP1623_cps GAAAAGTTTTTAGATGACCTTACAGTTACTGAAGTGGATATGATCTTGACAGATTTTACA 8820

************************************************************

PMP1486_cps GAACAACATGTTTACAACAATACTACTGTTCGAAATGATTTTGTTGAAAAGTATGAGGTT 8880

PMP1623_cps GAACAACATGTTTACAACAATACTACTGTTCGAAATGATTTTGTTGAAAAGTATGAGGTT 8880

************************************************************

PMP1486_cps GGTAAGGAATATTCTGGAATTCCAGAGAAACGGATTCCAATGCACTCGGTAACTTATAGA 8940

PMP1623_cps GGTAAGGAATATTCTGGAATTCCAGAGAAACGGATTCCAATGCACTCGGTAACTTATAGA 8940

************************************************************

PMP1486_cps ACATCTATCCTAGTTGAGAATGAAATTCGTTTAAGTGAAAAGACATTTTATGTTGATATT 9000

PMP1623_cps ACATCTATCCTAGTTGAGAATGAAATTCGTTTAAGTGAAAAGACATTTTATGTTGATATT 9000

************************************************************

PMP1486_cps CAGTACACTCTTTTTCCTTTAGAGTATGTTCATAGTTTCTGTTATTGGAATTATGATGTA 9060

PMP1623_cps CAGTACACTCTTTTTCCTTTAGAGTATGTTCATAGTTTCTGTTATTGGAATTATGATGTA 9060

************************************************************

PMP1486_cps TACCAATACTATATCGGACGACCCGAGCAGAGTATGAATATTGAGAGTATGAAACGAAAT 9120

PMP1623_cps TACCAATACTATATCGGACGACCCGAGCAGAGTATGAATATTGAGAGTATGAAACGAAAT 9120

************************************************************

PMP1486_cps GTTCGTCACCATTTGACTGTAACAAATTCTGTGCTGACTTATTTCTCAAAAATTGCGGAT 9180

PMP1623_cps GTTCGTCACCATTTGACTGTAACAAATTCTGTGCTGACTTATTTCTCAAAAATTGCGGAT 9180

************************************************************

PMP1486_cps GATCCTGTCTTAAAGAAAGTGGTTGCAGATACTTTAGTTTATCTTATCAGTTTGCAGATA 9240

PMP1623_cps GATCCTGTCTTAAAGAAAGTGGTTGCAGATACTTTAGTTTATCTTATCAGTTTGCAGATA 9240

************************************************************

PMP1486_cps GATTTGTCATGGATGGTTGAGGACTCAAAGACACTATCAGAAGAGTTATACAGACAAATT 9300

PMP1623_cps GATTTGTCATGGATGGTTGAGGACTCAAAGACACTATCAGAAGAGTTATACAGACAAATT 9300

************************************************************

PMP1486_cps GAGCAGAGTTCTTATGAGTATATCCCTACGAAAAAATTTGATAGATTGTCTTATTTGAAC 9360

PMP1623_cps GAGCAGAGTTCTTATGAGTATATCCCTACGAAAAAATTTGATAGATTGTCTTATTTGAAC 9360

************************************************************

PMP1486_cps TATAAGTTTCACTATTTTCTAGACTTTGTTTTCAATCCAGTATTGAAAAAATATTCTAAA 9420

PMP1623_cps TATAAGTTTCACTATTTTCTAGACTTTGTTTTCAATCCAGTATTGAAAAAATATTCTAAA 9420

************************************************************

PMP1486_cps AAGAAAGAAAAAGAGAGAGGAATTTAGAATGTTCATTAGTGTTGTTGTTCCGGTTTATAA 9480

PMP1623_cps AAGAAAGAAAAAGAGAGAGGAATTTAGAATGTTCATTAGTGTTGTTGTTCCGGTTTATAA 9480

************************************************************

PMP1486_cps TGTTTTTGACTATTTACACTTTGCCATGGATAGTCTGATAAAGCAAACGTATCAAAATTT 9540

PMP1623_cps TGTTTTTGACTATTTACACTTTGCCATGGATAGTCTGATAAAGCAAACGTATCAAAATTT 9540

************************************************************

PMP1486_cps TGAAGTAATCCTTGTAAATGATGGTTCAACAGATAACTCTCCTCAGTTATGTGAAGAGTA 9600

PMP1623_cps TGAAGTAATCCTTGTAAATGATGGTTCAACAGATAACTCTCCTCAGTTATGTGAAGAGTA 9600

************************************************************

PMP1486_cps TGCTAAGCAATATGAAAACGTCTCTGTTTTTCATAAAGAAAATGGAGGGTTGTCTGATGC 9660

PMP1623_cps TGCTAAGCAATATGAAAACGTCTCTGTTTTTCATAAAGAAAATGGAGGGTTGTCTGATGC 9660

************************************************************

PMP1486_cps TCGTAATTTTGGAGTTTCAAAAGCATCATCAGATTGGATTTTCTTTCTAGATCCAGACGA 9720

PMP1623_cps TCGTAATTTTGGAGTTTCAAAAGCATCATCAGATTGGATTTTCTTTCTAGATCCAGACGA 9720

************************************************************

PMP1486_cps TTATTTGGAAGATTATACTCTAGAATTGATAGTGAAAATTCAACAGGAATATCAAGCAAA 9780

PMP1623_cps TTATTTGGAAGATTATACTCTAGAATTGATAGTGAAAATTCAACAGGAATATCAAGCAAA 9780

************************************************************

PMP1486_cps CTTGATTTCAACCAAAGTAAAGGCAACTTCTAAATATAATGATTATAGTCTTTATCAACT 9840

PMP1623_cps CTTGATTTCAACCAAAGTAAAGGCAACTTCTAAATATAATGATTATAGTCTTTATCAACT 9840

************************************************************

PMP1486_cps TGAGGAGTCAGATTATAAAGATTTGTTTGTCGTTACAAAAGAGAAGGCTCTTGAGCTAAT 9900

PMP1623_cps TGAGGAGTCAGATTATAAAGATTTGTTTGTCGTTACAAAAGAGAAGGCTCTTGAGCTAAT 9900

************************************************************

PMP1486_cps GCTAGACGACAAAATTGCGACAGTTTCTGCTTGTGCTAAGCTTTATCACAAGAATATCTT 9960

PMP1623_cps GCTAGACGACAAAATTGCGACAGTTTCTGCTTGTGCTAAGCTTTATCACAAGAATATCTT 9960

************************************************************

PMP1486_cps GGAAAAAGTTCCATTTTCAGTTGGAAAAATCTATGAAGATTTTTATGTGGTCGCGGATCA 10020

PMP1623_cps GGAAAAAGTTCCATTTTCAGTTGGAAAAATCTATGAAGATTTTTATGTGGTCGCGGATCA 10020

************************************************************

PMP1486_cps TCTTGCCTTAGCAGATAAAATTGTAATTAGTCCACTTGAAACGTATAACTATTACCGCAG 10080

PMP1623_cps TCTTGCCTTAGCAGATAAAATTGTAATTAGTCCACTTGAAACGTATAACTATTACCGCAG 10080

************************************************************

PMP1486_cps AGAAGGTAGTATTGTTCGTTCGACTTTTACTGAGAAAAGATATGATTTTTTTGATGCTGT 10140

PMP1623_cps AGAAGGTAGTATTGTTCGTTCGACTTTTACTGAGAAAAGATATGATTTTTTTGATGCTGT 10140

************************************************************

PMP1486_cps TTCGAAAAACGAAGAAGTTATAAATAAAGAATACACACAAAGTTTAGAATTACAACAATC 10200

PMP1623_cps TTCGAAAAACGAAGAAGTTATAAATAAAGAATACACACAAAGTTTAGAATTACAACAATC 10200

************************************************************

PMP1486_cps TCTGCAAGCGAAAAAATTACGAGGAGGTTTTGTTGTAATTGGTGCGAAAGCTGACTCTGG 10260

PMP1623_cps TCTGCAAGCGAAAAAATTACGAGGAGGTTTTGTTGTAATTGGTGCGAAAGCTGACTCTGG 10260

************************************************************

PMP1486_cps TTTAACAGATTTTTCGAAAGATAGAGACCTATTAAAAGTTGATTTTAAAAACATGTTATT 10320

PMP1623_cps TTTAACAGATTTTTCGAAAGATAGAGACCTATTAAAAGTTGATTTTAAAAACATGTTATT 10320

************************************************************

PMP1486_cps AAATAAAAAAATATCATGGAAACTAAAATTAAAATATACAATATTTATGCTATCATCTAA 10380

PMP1623_cps AAATAAAAAAATATCATGGAAACTAAAATTAAAATATACAATATTTATGCTATCATCTAA 10380

************************************************************

PMP1486_cps AATGTATTTAAGATTAAGGTGACTGTTTCAAGAATCAAGCATCTCTATTAGTTTTTAAAA 10440

PMP1623_cps AATGTATTTAAGATTAAGGTGACTGTTTCAAGAATCAAGCATCTCTATTAGTTTTTAAAA 10440

************************************************************

PMP1486_cps TTATAAATAGCTGTTCGGATGTAAGTGTTGTAATAGTGTTTAGTATTTCTAGTTCATTAT 10500

PMP1623_cps TTATAAATAGCTGTTCGGATGTAAGTGTTGTAATAGTGTTTAGTATTTCTAGTTCATTAT 10500

************************************************************

PMP1486_cps TATAATCGGGAAAGAATTTAATACAGCTACTTATTTGAAAGGGAAATTCATGAGACAAGC 10560

PMP1623_cps TATAATCGGGAAAGAATTTAATACAGCTACTTATTTGAAAGGGAAATTCATGAGACAAGC 10560

************************************************************

PMP1486_cps ATATCTAATAATAGCACATAATAAGTTTGAATAGTTGAAATTTTTAATTTCTCTGTTGGA 10620

PMP1623_cps ATATCTAATAATAGCACATAATAAGTTTGAATAGTTGAAATTTTTAATTTCTCTGTTGGA 10620

************************************************************

PMP1486_cps TTATAAGGAACATAATATTTTTATTATTGTGGACAGCAAAGTTAATGCTGAGGAGTCTAC 10680

PMP1623_cps TTATAAGGAACATAATATTTTTATTATTGTGGACAGCAAAGTTAATGCTGAGGAGTCTAC 10680

************************************************************

PMP1486_cps GATTAATCACTATACTTCCCATCATTATAACTTCCTATGTGACATGGATATTTTAACTCT 10740

PMP1623_cps GATTAATCACTATACTTCCCATCATTATAACTTCCTATGTGACATGGATATTTTAACTCT 10740

************************************************************

PMP1486_cps ATTGTTACCTATTTTGTTAGTCACTTTAGGAGTTGCTAGTTATGGCATTAAGGAAATTTA 10800

PMP1623_cps ATTGTTACCTATTTTGTTAGTCACTTTAGGAGTTGCTAGTTATGGCATTAAGGAAATTTA 10800

************************************************************

PMP1486_cps AGCTAATCGAAAAAAGAGCCACAGAATTTTTGGGGTATCTGTAAAACTTAAAATGAAGTA 10860

PMP1623_cps AGCTAATCGAAAAAAGAGCCACAGAATTTTTGGGGTATCTGTAAAACTTAAAATGAAGTA 10860

************************************************************

PMP1486_cps TAGTTTATTTTGAGTTTAATAAGCGATTAAAAGGTAGGGGGAATATTATTGAAGATTAAC 10920

PMP1623_cps TAGTTTATTTTGAGTTTAATAAGCGATTAAAAGGTAGGGGGAATATTATTGAAGATTAAC 10920

************************************************************

PMP1486_cps TTAATGAATATTGTTGATACGTTACTGCTTTTGCTAATAATTATAAAAACAAACTCTATT 10980

PMP1623_cps TTAATGAATATTGTTGATACGTTACTGCTTTTGCTAATAATTATAAAAACAAACTCTATT 10980

************************************************************

PMP1486_cps TATTTGTATATAGGTGTAGAGAACAAATTAAATTTTTATCTGATTGTTGTGATTACAATT 11040

PMP1623_cps TATTTGTATATAGGTGTAGAGAACAAATTAAATTTTTATCTGATTGTTGTGATTACAATT 11040

************************************************************

PMP1486_cps GCAGTTTTATTGGAAGTAATTAGCGGTAGATTATCTCTCCAATATTTAAAAAAAATGCTA 11100

PMP1623_cps GCAGTTTTATTGGAAGTAATTAGCGGTAGATTATCTCTCCAATATTTAAAAAAAATGCTA 11100

************************************************************

PMP1486_cps TTAGTTATTGTTCTTTATTTTTTTGCAATTATTATTAATATTTTAGTTTCGGCTTCTGTC 11160

PMP1623_cps TTAGTTATTGTTCTTTATTTTTTTGCAATTATTATTAATATTTTAGTTTCGGCTTCTGTC 11160

************************************************************

PMP1486_cps ATATCGTCAAATATACTAACATACTATTTTATTGTTGTGCCATTAATGCTAATACTAGTA 11220

PMP1623_cps ATATCGTCAAATATACTAACATACTATTTTATTGTTGTGCCATTAATGCTAATACTAGTA 11220

************************************************************

PMP1486_cps ATGTATAAATATTATAAAAATACATTAACAAATTTTTTAGTTATCTTTGTGAGGATTGTT 11280

PMP1623_cps ATGTATAAATATTATAAAAATACATTAACAAATTTTTTAGTTATCTTTGTGAGGATTGTT 11280

************************************************************

PMP1486_cps TTAATTTTAGCTGTTATTTCTCTGCTCTTTTGGTGTTTTGGTAGTGTTTTAAATATAATA 11340

PMP1623_cps TTAATTTTAGCTGTTATTTCTCTGCTCTTTTGGTGTTTTGGTAGTGTTTTAAATATAATA 11340

************************************************************

PMP1486_cps AAACCGACAAACTATGTGGTGAGTAGCTGGAGTGGTGGTCAGGTAACTACTAGTTATTAT 11400

PMP1623_cps AAACCGACAAACTATGTGGTGAGTAGCTGGAGTGGTGGTCAGGTAACTACTAGTTATTAT 11400

************************************************************

PMP1486_cps AATCTGTATTTTGAAACGCAAAACGCTTTATTTTTGGGTTATAAAATGATTAGGAATAGC 11460

PMP1623_cps AATCTGTATTTTGAAACGCAAAACGCTTTATTTTTGGGTTATAAAATGATTAGGAATAGC 11460

************************************************************

PMP1486_cps GGTATTTTTGCAGAAGCGCCAATGTGGAGTTTATTGCTAAGTGTTGCTTTAATATTTCAA 11520

PMP1623_cps GGTATTTTTGCAGAAGCGCCAATGTGGAGTTTATTGCTAAGTGTTGCTTTAATATTTCAA 11520

************************************************************

PMP1486_cps GAGTTATTGCTTAAACATAGCACTAGAATATTTGTTTTATTAATGTTGACAATTTTTACA 11580

PMP1623_cps GAGTTATTGCTTAAACATAGCACTAGAATATTTGTTTTATTAATGTTGACAATTTTTACA 11580

************************************************************

PMP1486_cps ACAGCATCAACAACAGGTTTTTTTATAGTTGGTTCATTACTTATTTATAAAGTGATAAAT 11640

PMP1623_cps ACAGCATCAACAACAGGTTTTTTTATAGTTGGTTCATTACTTATTTATAAAGTGATAAAT 11640

************************************************************

PMP1486_cps CAAAAAAGAAGTTGGTTTAAATATATCAACTTGACATCTATCCCTGTATTAATCTTTACT 11700

PMP1623_cps CAAAAAAGAAGTTGGTTTAAATATATCAACTTGACATCTATCCCTGTATTAATCTTTACT 11700

************************************************************

PMP1486_cps TTGGTTAAAGTGTGGGGGGAAAAATCCGATTCCGCTTCAGCAAGTATTCGATATGATGAT 11760

PMP1623_cps TTGGTTAAAGTGTGGGGGGAAAAATCCGATTCCGCTTCAGCAAGTATTCGATATGATGAT 11760

************************************************************

PMP1486_cps TATGTAGCAGGTTTTTTGGCATGGAAAAATCACTTTATTTTTGGTTCAGGTTTATCATCT 11820

PMP1623_cps TATGTAGCAGGTTTTTTGGCATGGAAAAATCACTTTATTTTTGGTTCAGGTTTATCATCT 11820

************************************************************

PMP1486_cps GGGATTAGAGCGATTGAGTCCTATATGGATACCACTATACGAAGTAATTTAGGATACAGT 11880

PMP1623_cps GGGATTAGAGCGATTGAGTCCTATATGGATACCACTATACGAAGTAATTTAGGATACAGT 11880

************************************************************

PMP1486_cps AATAGTTTTTTTGTCATCTTAGCTCAAGGGGGAATAATTTTGGGCGTGCTACACTTTTAT 11940

PMP1623_cps AATAGTTTTTTTGTCATCTTAGCTCAAGGGGGAATAATTTTGGGCGTGCTACACTTTTAT 11940

************************************************************

PMP1486_cps CCTGTTGTTAGTGTACTATTGAAGAGATTTTCATCAAACTCTAAGATGTTAGCTTTATTG 12000

PMP1623_cps CCTGTTGTTAGTGTACTATTGAAGAGATTTTCATCAAACTCTAAGATGTTAGCTTTATTG 12000

************************************************************

PMP1486_cps TTTATAATTCTAATATTTACAGCAATATTTACAGATACACCGTTATTTATCCTGTTTGTT 12060

PMP1623_cps TTTATAATTCTAATATTTACAGCAATATTTACAGATACACCGTTATTTATCCTGTTTGTT 12060

************************************************************

PMP1486_cps GGGATATTTTATGCCTTGATTCTGAATAGAGAGAATACATGAAGAAGGAATATGATATTT 12120

PMP1623_cps GGGATATTTTATGCCTTGATTCTGAATAGAGAGAATACATGAAGAAGGAATATGATATTT 12120

************************************************************

PMP1486_cps TAAAAGTAATTGCCATTTTAATGGTTGTATTAAGCCACAGTACATACTATGTGATTTCGA 12180

PMP1623_cps TAAAAGTAATTGCCATTTTAATGGTTGTATTAAGCCACAGTACATACTATGTGATTTCGA 12180

************************************************************

PMP1486_cps CTAAGTACGGGGGGATTATCAACAATATATAAATCAAAATTTATCGTTGGTATTGTATAA 12240

PMP1623_cps CTAAGTACGGGGGGATTATCAACAATATATAAATCAAAATTTATCGTTGGTATTGTATAA 12240

************************************************************

PMP1486_cps AGTTTTTGATAAAGTAAGAGAAGTATTATATTACTTCCATATGCCACTTTTTATGGCATT 12300

PMP1623_cps AGTTTTTGATAAAGTAAGAGAAGTATTATATTACTTCCATATGCCACTTTTTATGGCATT 12300

************************************************************

PMP1486_cps ATCAGGAGCTTTCTACTATCTTCAGGTTCAAAGAGATAAATGGTTTACTTTAAAATTAAT 12360

PMP1623_cps ATCAGGAGCTTTCTACTATCTTCAGGTTCAAAGAGATAAATGGTTTACTTTAAAATTAAT 12360

************************************************************

PMP1486_cps TGTGCAAAATAAAATAAGGTAAAGAGATTGCTTTTTCCTTTCATTATATTTACTGTCCTT 12420

PMP1623_cps TGTGCAAAATAAAATAAGGTAAAGAGATTGCTTTTTCCTTTCATTATATTTACTGTCCTT 12420

************************************************************

PMP1486_cps TATTCAATACCAATAAAATATATTTCAAATTATTTTGATTTTACAGCTCCTTTTAAAGCA 12480

PMP1623_cps TATTCAATACCAATAAAATATATTTCAAATTATTTTGATTTTACAGCTCCTTTTAAAGCA 12480

************************************************************

PMP1486_cps TTTGTAGGAGAATTTTTCTTAATTGGAAATAGTCATTTATGGTATTTATATGCTTTATTT 12540

PMP1623_cps TTTGTAGGAGAATTTTTCTTAATTGGAAATAGTCATTTATGGTATTTATATGCTTTATTT 12540

************************************************************

PMP1486_cps ATTATTTTTATATTTGCATTCTATACGCTAAAAAAGAAACAAATCTTGCTACTTATGTTG 12600

PMP1623_cps ATTATTTTTATATTTGCATTCTATACGCTAAAAAAGAAACAAATCTTGCTACTTATGTTG 12600

************************************************************

PMP1486_cps TTTTTTATGTTCTGTATATTTTGAGTTACAAAATAGAACTCACACTATTTAAAGTACCTC 12660

PMP1623_cps TTTTTTATGTTCTGTATATTTTGAGTTACAAAATAGAACTCACACTATTTAAAGTACCTC 12660

************************************************************

PMP1486_cps TTCAATTTTTATTTTACTTTAGTTTAGGCTTCTTGTTTGAATCAAATAGAGAAAAATATA 12720

PMP1623_cps TTCAATTTTTATTTTACTTTAGTTTAGGCTTCTTGTTTGAATCAAATAGAGAAAAATATA 12720

************************************************************

PMP1486_cps ATCAATTTATTAATAGGAAAAAAATTATATTTTGTTATTATCTACGGTATTTGTCTTAAT 12780

PMP1623_cps ATCAATTTATTAATAGGAAAAAAATTATATTTTGTTATTATCTACGGTATTTGTCTTAAT 12780

************************************************************

PMP1486_cps GGTTTTGTTAAATTTACTCAGGTATTTTTTAGTAAAGTATTAGTTGAGCTATTGGCTGTC 12840

PMP1623_cps GGTTTTGTTAAATTTACTCAGGTATTTTTTAGTAAAGTATTAGTTGAGCTATTGGCTGTC 12840

************************************************************

PMP1486_cps TTGGGTTCATTATTAACGTATAGCATCGCATATCAATTATCTCAAAAGAAAAGTGTTGGT 12900

PMP1623_cps TTGGGTTCATTATTAACGTATAGCATCGCATATCAATTATCTCAAAAGAAAAGTGTTGGT 12900

************************************************************

PMP1486_cps GATGCTAGTATTTTTAAGATGATTCTAATTAATGGATTAGGTATATATATTTTTTCTGAT 12960

PMP1623_cps GATGCTAGTATTTTTAAGATGATTCTAATTAATGGATTAGGTATATATATTTTTTCTGAT 12960

************************************************************

PMP1486_cps CCATTAAATTATTTAATCTTGAAGTTAAGTTATTCCCTAAATTCGTATTTTATGTTTACA 13020

PMP1623_cps CCATTAAATTATTTAATCTTGAAGTTAAGTTATTCCCTAAATTCGTATTTTATGTTTACA 13020

************************************************************

PMP1486_cps CCAGTAGGAATAATTGTTTTAGTAGTATTACGATTTTTTCTCACGTTATTCATATCATTG 13080

PMP1623_cps CCAGTAGGAATAATTGTTTTAGTAGTATTACGATTTTTTCTCACGTTATTCATATCATTG 13080

************************************************************

PMP1486_cps ATAGGAACAATCATATTTAAAAAAATACAAATGGCTAGTCAACTAGGTAGAGACAATAGC 13140

PMP1623_cps ATAGGAACAATCATATTTAAAAAAATACAAATGGCTAGTCAACTAGGTAGAGACAATAGC 13140

************************************************************

PMP1486_cps TATTTAAAAAGTTAAATCTATATATAATGGAGAAAAAGTAATGAAAGTACTTAAAAACTA 13200

PMP1623_cps TATTTAAAAAGTTAAATCTATATATAATGGAGAAAAAGTAATGAAAGTACTTAAAAACTA 13200

************************************************************

PMP1486_cps CGCCTACAATCTTTCTTATCAATTGTTGGTGATTTTACTCCCGATTATTACGACTCCCTA 13260

PMP1623_cps CGCCTACAATCTTTCTTATCAATTGTTGGTGATTTTACTCCCGATTATTACGACTCCCTA 13260

************************************************************

PMP1486_cps TGTAACACGGGTCTTTTCTTCGGATGATTTAGGGACGTATGGTTATTTTAATTCCATCGT 13320

PMP1623_cps TGTAACACGGGTCTTTTCTTCGGATGATTTAGGGACGTATGGTTATTTTAATTCCATCGT 13320

************************************************************

PMP1486_cps TACTTATTTTATCCTCTTAGCGACGCTAGGAGTTGCTAACTATGGGACCAAGGTCATTTC 13380

PMP1623_cps TACTTATTTTATCCTCTTAGCGACGCTAGGAGTTGCTAACTATGGGACCAAGGTCATTTC 13380

************************************************************

PMP1486_cps AGGGCATCGAAAGCAAATTCAAAAAAACTTTTTGGGAATCTATTCTCTGCAATTAGGTGC 13440

PMP1623_cps AGGGCATCGAAAGCAAATTCAAAAAAACTTTTTGGGAATCTATTCTCTGCAATTAGGTGC 13440

************************************************************

PMP1486_cps AACAGTTCTTTCTCTGTCCTTGTATGCTCTTCTTTGTCTAACTCTTCCCTTTATGCAAAA 13500

PMP1623_cps AACAGTTCTTTCTCTGTCCTTGTATGCTCTTCTTTGTCTAACTCTTCCCTTTATGCAAAA 13500

************************************************************

PMP1486_cps TCCGGTAGTCTATATTCTAGGCTTGAGTTTAGTTTCTAAAGGTTTAGACATCTCCTGGCT 13560

PMP1623_cps TCCGGTAGTCTATATTCTAGGCTTGAGTTTAGTTTCTAAAGGTTTAGACATCTCCTGGCT 13560

************************************************************

PMP1486_cps CTTTCAAGGGTTGGAGGATTTTCGAAAGATTACTGTCCGAAATATAACAGTTAAACTAGT 13620

PMP1623_cps CTTTCAAGGGTTGGAGGATTTTCGAAAGATTACTGTCCGAAATATAACAGTTAAACTAGT 13620

************************************************************

PMP1486_cps TGGGGTCATTTCCATCTTCCTCTTTGTTAAATCATCAAATGACCTTTACCTCTATGTCTT 13680

PMP1623_cps TGGGGTCATTTCCATCTTCCTCTTTGTTAAATCATCAAATGACCTTTACCTCTATGTCTT 13680

************************************************************

PMP1486_cps TTTGCTAACCATTTTTGAACTCTTGGGTCAATTCAGTATGTGGATACCAGCTCGAGAGTT 13740

PMP1623_cps TTTGCTAACCATTTTTGAACTCTTGGGTCAATTCAGTATGTGGATACCAGCTCGAGAGTT 13740

************************************************************

PMP1486_cps TATTGGTAGACCTCATTTTGACATAGAATATGCTAGACATCATTTGAAACCAGTCATATT 13800

PMP1623_cps TATTGGTAGACCTCATTTTGACATAGAATATGCTAGACATCATTTGAAACCAGTCATATT 13800

************************************************************

PMP1486_cps ATTGTTCCTTCCGCAAGTGGCGATTTCCTTGTATGTTACGCTAAATCGTACTATGCTTGG 13860

PMP1623_cps ATTGTTCCTTCCGCAAGTGGCGATTTCCTTGTATGTTACGCTAAATCGTACTATGCTTGG 13860

************************************************************

PMP1486_cps AGCGTTAGCTTCTACAAAAGATGTAGGGATTTATGACCAGGCTTTAAAGTTGGTAACTAT 13920

PMP1623_cps AGCGTTAGCTTCTACAAAAGATGTAGGGATTTATGACCAGGCTTTAAAGTTGGTAACTAT 13920

************************************************************

PMP1486_cps CCTTCTGACCTTGGTAACTTCCTTGGGAAGCGTTATGTTACCTCGAGTCGCTCATTTGTT 13980

PMP1623_cps CCTTCTGACCTTGGTAACTTCCTTGGGAAGCGTTATGTTACCTCGAGTCGCTCATTTGTT 13980

************************************************************

PMP1486_cps AGCGACAGATGATCATAAGGCAGTCAATAGGATGCATGAAATGTCTTTCTTAATTTATAA 14040

PMP1623_cps AGCGACAGATGATCATAAGGCAGTCAATAGGATGCATGAAATGTCTTTCTTAATTTATAA 14040

************************************************************

PMP1486_cps TTTAGTAATTTTTCCAATGATGGCAGGAATCTTGATTGTCAATGATGATTTTGTTCAGTT 14100

PMP1623_cps TTTAGTAATTTTTCCAATGATGGCAGGAATCTTGATTGTCAATGATGATTTTGTTCAGTT 14100

************************************************************

PMP1486_cps TTTCCTTGGTCAAGATTTTCAGGATGCGCGTTATGCAATCGCCATTATGATCTTCCGTAT 14160

PMP1623_cps TTTCCTTGGTCAAGATTTTCAGGATGCGCGTTATGCAATCGCCATTATGATCTTCCGTAT 14160

************************************************************

PMP1486_cps GTTCTTTATCGGTTGGACCAATATCATGGGAATTCAGATGCTGATACCTCATAATCAAAA 14220

PMP1623_cps GTTCTTTATCGGTTGGACCAATATCATGGGAATTCAGATGCTGATACCTCATAATCAAAA 14220

************************************************************

PMP1486_cps TAAAGAATTCATGATTTCAACAACAGCTCCCGCAATTATCAGTGTAGGTTTGAACTTACT 14280

PMP1623_cps TAAAGAATTCATGATTTCAACAACAGCTCCCGCAATTATCAGTGTAGGTTTGAACTTACT 14280

************************************************************

PMP1486_cps ATTCCTTCCTAAACTGGGATATATCGGAGCAGCCATTGTCTCTGTTTTAACAGAGGCACT 14340

PMP1623_cps ATTCCTTCCTAAACTGGGATATATCGGAGCAGCCATTGTCTCTGTTTTAACAGAGGCACT 14340

************************************************************

PMP1486_cps TGTATGGGCAATCCAATTATTCTATACTCGCAGATATTTAAAAGAAGTTCCTATAATCGG 14400

PMP1623_cps TGTATGGGCAATCCAATTATTCTATACTCGCAGATATTTAAAAGAAGTTCCTATAATCGG 14400

************************************************************

PMP1486_cps ATCAATGTCAAAAATTATACTAGCATCTGCCATTATGTATGGCCTTTTACTAAGTTCAAA 14460

PMP1623_cps ATCAATGTCAAAAATTATACTAGCATCTGCCATTATGTATGGCCTTTTACTAAGTTCAAA 14460

************************************************************

PMP1486_cps AACAGTTATACATTTTTCACCGACCTTAAATGTTCTAGCATTTGCAGCGCTTGGTGGAAT 14520

PMP1623_cps AACAGTTATACATTTTTCACCGACCTTAAATGTTCTAGCATTTGCAGCGCTTGGTGGAAT 14520

************************************************************

PMP1486_cps CATTTATCTTTTTGCAATTCTATCTCTGAAAGTGGTAGATGTGAAAGAATTAAAACAAAT 14580

PMP1623_cps CATTTATCTTTTTGCAATTCTATCTCTGAAAGTGGTAGATGTGAAAGAATTAAAACAAAT 14580

************************************************************

PMP1486_cps TATTAGGAAAAACTAAAATGAGAAAAATTCGAAATATCAACCTAGATTTACTAAAAGTGC 14640

PMP1623_cps TATTAGGAAAAACTAAAATGAGAAAAATTCGAAATATCAACCTAGATTTACTAAAAGTGC 14640

************************************************************

PMP1486_cps TTGCATGTGTTGGAGTTGTTTTACTTCATACAACAATGGGCGGATTTAAAGAGACAGGCT 14700

PMP1623_cps TTGCATGTGTTGGAGTTGTTTTACTTCATACAACAATGGGCGGATTTAAAGAGACAGGCT 14700

************************************************************

PMP1486_cps CATATAATCTTTTGGCATATTTATATTATTTAGGTACTTACTCTATTCCCCTG-TTTTTT 14759

PMP1623_cps CATATAATCTTTTGGCATATTTATATTATTTAGGTACTTACTCTATTCCCCTGTTTTTTT 14760

***************************************************** ******

PMP1486_cps ATGATCAATGGTTATTTATTGTTAGGCAAGAGGGAAATAACTTATCTTTACATACTCCAG 14819

PMP1623_cps ATGATCAATGGTTATTTATTGTTAGGCAAGAGGGAAATAACTTATCTTTACATACTCCAG 14820

************************************************************

PMP1486_cps AAAGTAAAATGGATTTTAATAACAGTGTCATCATGGACATTTATCGTATGGCTTTTTTAT 14879

PMP1623_cps AAAGTAAAATGGATTTTAATAACAGTGTCATCATGGACATTTATCGTATGGCTTTTTTAT 14880

************************************************************

PMP1486_cps CGTGATTTTACAACTAATCCTATTAAAAAAATTGTAGGTTCTTTGATACAAAGAGGTTAT 14939

PMP1623_cps CGTGATTTTACAACTAATCCTATTAAAAAAATTGTAGGTTCTTTGATACAAAGAGGTTAT 14940

************************************************************

PMP1486_cps TTCTCTCAGTTTTGGTTTTTCGGTGCACTAATACTTATCTATTTATGTTTGCCAATTGTG 14999

PMP1623_cps TTCTCTCAGTTTTGGTTTTTCGGTGCACTAATACTTATCTATTTATGTTTGCCAATTGTG 15000

************************************************************

PMP1486_cps AGACAATTTCTAAATTCAAAAAGAAGCTATTTATACAGTTTATCTTTATTGATGACTATT 15059

PMP1623_cps AGACAATTTCTAAATTCAAAAAGAAGCTATTTATACAGTTTATCTTTATTGATGACTATT 15060

************************************************************

PMP1486_cps GGTTTGATTTTTGAGTTATTAAATATCCTACTTCAGATGCCAATACAAACATATGTAATA 15119

PMP1623_cps GGTTTGATTTTTGAGTTATTAAATATCCTACTTCAGATGCCAATACAAACATATGTAATA 15120

************************************************************

PMP1486_cps CAGACTTTTAGATTATGGACGTGGTTTTTTTACTATCTTTTAGGTGGTTATATAGCGCAA 15179

PMP1623_cps CAGACTTTTAGATTATGGACGTGGTTTTTTTACTATCTTTTAGGTGGTTATATAGCGCAA 15180

************************************************************

PMP1486_cps TTCACTAAAGAAGAAATCGAATCAAGATTTAAGAATTGGATGAAAATAGCTAGCATACTT 15239

PMP1623_cps TTCACTAAAGAAGAAATCGAATCAAGATTTAAGAATTGGATGAAAATAGCTAGCATACTT 15240

************************************************************

PMP1486_cps TTGTTATTGATTTCACCAATAATATTATTTTTCATAGCAAAGACCACTTACCATAATTTT 15299

PMP1623_cps TTGTTATTGATTTCACCAATAATATTATTTTTCATAGCAAAGACCACTTACCATAATTTT 15300

************************************************************

PMP1486_cps TTTGCTGAATATTTTTATGATATTTTATTAGTAAAAGTGGTAAGCGTAGGGATTTTTCTA 15359

PMP1623_cps TTTGCTGAATATTTTTATGATATTTTATTAGTAAAAGTGGTAAGCGTAGGGATTTTTCTA 15360

************************************************************

PMP1486_cps ACTATTTTCTCACTTGTATTGAATGAAAATAGCAACAAATGGATTATTTTTCTTTCTAAT 15419

PMP1623_cps -----------ACTTGTATTGAATGAAAATAGCAACAAATGGATTATTTTTCTTTCTAAT 15409

*************************************************

PMP1486_cps CAAACTATGGGTATCTTTATAATACACACTTATATTATGAAGGTATGGGAAAAACTATTT 15479

PMP1623_cps CAAACTATGGGTATCTTTATAATACACACTTATATTATGAAGGTATGGGAAAAACTATTT 15469

************************************************************

PMP1486_cps GGTTTTAGTTTTATAGGTTCATATTTACTTTTTGCTATATTTACTTTAAGTGTTAGTTTT 15539

PMP1623_cps GGTTTTAGTTTTATAGGTTCATATTTACTTTTTGCTATATTTACTTTAAGTGTTAGTTTT 15529

************************************************************

PMP1486_cps ATCATTGTTGGAATGTTAATGAAAATTCCGTATTTTAATCGAATCGTCAAATTATAAAAA 15599

PMP1623_cps ATCATTGTTGGAATGTTAATGAAAATTCCGTATTTTAATCGAATCGTCAAATTATAAAAA 15589

************************************************************

PMP1486_cps GGAGAAAAAATGTACGATTATCTTGTTGTTGGTGCTGGTCTCTTTGGTGCAGTCTTTGCC 15659

PMP1623_cps GGAGAAAAAATGTACGATTATCTTGTTGTTGGTGCTGGTCTCTTTGGTGCAGTCTTTGCC 15649

************************************************************

PMP1486_cps CATGAAGCAGCCTTAAAAGGAAAAAAAGTAAAAGTTATTGAAAAACGAAATCATATCGCG 15719

PMP1623_cps CATGAAGCAGCCTTAAAAGGAAAAAAAGTAAAAGTTATTGAAAAACGAAATCATATCGCG 15709

************************************************************

PMP1486_cps GGTAATATCTATACTCGTGAAGAGGAAGGAATTCAAGTTCATCAATATGGTGCTCATATC 15779

PMP1623_cps GGTAATATCTATACTCGTGAAGAGGAAGGAATTCAAGTTCATCAATATGGTGCTCATATC 15769

************************************************************

PMP1486_cps TTCCATACTTCTGATAAGGAGATCTGGGATTATGTAAATCAGTTTGCAGAGTTTAACCGT 15839

PMP1623_cps TTCCATACTTCTGATAAGGAGATCTGGGATTATGTAAATCAGTTTGCAGAGTTTAACCGT 15829

************************************************************

PMP1486_cps TATACCAATTCTCCTGTTGCAAACTATAAGGGTGAGATTTATAACCTTCCTTTTAATATG 15899

PMP1623_cps TATACCAATTCTCCTGTTGCAAACTATAAGGGTGAGATTTATAACCTTCCTTTTAATATG 15889

************************************************************

PMP1486_cps AATACTTTCAATAAACTCTGGGGAGTTGTAACGCCAGCAGAAGCACAAGCTAAGATTGAG 15959

PMP1623_cps AATACTTTCAATAAACTCTGGGGAGTTGTAACGCCAGCAGAAGCACAAGCTAAGATTGAG 15949

************************************************************

PMP1486_cps GAACAACGTGCTATTTTAAATGGTAAAACTCCTGAAAATTTGGAAGAACAGGCGATTTCT 16019

PMP1623_cps GAACAACGTGCTATTTTAAATGGTAAAACTCCTGAAAATTTGGAAGAACAGGCGATTTCT 16009

************************************************************

PMP1486_cps CTTGTAGGTACAGACATCTACGAAAAATTAATCAAAGACTATACAGAGAAACAGTGGGGC 16079

PMP1623_cps CTTGTAGGTACAGACATCTACGAAAAATTAATCAAAGACTATACAGAGAAACAGTGGGGC 16069

************************************************************

PMP1486_cps AAACCAACTACTGAACTTCCATCCTTTATTATTCGCCGTTTGCCAGTACGCCTGACCTAT 16139

PMP1623_cps AAACCAACTACTGAACTTCCATCCTTTATTATTCGCCGTTTGCCAGTACGCCTGACCTAT 16129

************************************************************

PMP1486_cps GATAACAACTATTTTAACGATACCTATCAAGGGATTCCAATTGGTGGATACACTCAAATA 16199

PMP1623_cps GATAACAACTATTTTAACGATACCTATCAAGGGATTCCAATTGGTGGATACACTCAAATA 16189

************************************************************

PMP1486_cps GTTGAAAAAATGTTGGATCATGAAAATATTGATGTAGAAACAAATGTTGATTTCTTTGTG 16259

PMP1623_cps GTTGAAAAAATGTTGGATCATGAAAATATTGATGTAGAAACAAATGTTGATTTCTTTGTG 16249

************************************************************

PMP1486_cps AATAAAGAGCAATATCTGAAAGATTTTCCTAAGATTGTCTTTACTGGTATGATTGATGAA 16319

PMP1623_cps AATAAAGAGCAATATCTGAAAGATTTTCCTAAGATTGTCTTTACTGGTATGATTGATGAA 16309

************************************************************

PMP1486_cps TTCTTTGACTATAAGTTGGGTGAACTAGAGTACCGTAGTCTTCGTTTTGAAAATGAGACC 16379

PMP1623_cps TTCTTTGACTATAAGTTGGGTGAACTAGAGTACCGTAGTCTTCGTTTTGAAAATGAGACC 16369

************************************************************

PMP1486_cps TTGGATATGGAAAATTACCAAGGAAATGCAGTTGTGAACTATACGGATGCAGAAACTCCA 16439

PMP1623_cps TTGGATATGGAAAATTACCAAGGAAATGCAGTTGTGAACTATACGGATGCAGAAACTCCA 16429

************************************************************

PMP1486_cps TATACTCGCATTATTGAACACAAACATTTTGAGTTTGGGAGTCAAGCAAAGACTATCATT 16499

PMP1623_cps TATACTCGCATTATTGAACACAAACATTTTGAGTTTGGGAGTCAAGCAAAGACTATCATT 16489

************************************************************

PMP1486_cps ACTAAAGAACATTCTAAAACATGGGAAAAAGGTGATGAGCCTTATTATCCAGTTAATAAT 16559

PMP1623_cps ACTAAAGAACATTCTAAAACATGGGAAAAAGGTGATGAGCCTTATTATCCAGTTAATAAT 16549

************************************************************

PMP1486_cps GATCGTAATAATCATTTGTATAAATCATATAAAAAACTTGCTGATGAGCAAGGGAATGTT 16619

PMP1623_cps GATCGTAATAATCATTTGTATAAATCATATAAAAAACTTGCTGATGAGCAAGGGAATGTT 16609

************************************************************

PMP1486_cps ATCTTTGGTGGCCGCTTAGGACACTATCGTTATTACGATATGCACCAAGTAATTGGAGTA 16679

PMP1623_cps ATCTTTGGTGGCCGCTTAGGACACTATCGTTATTACGATATGCACCAAGTAATTGGAGTA 16669

************************************************************

PMP1486_cps GCTTTGCAGTGCGTGAGAAATGAGTTAAATTAA 16712

PMP1623_cps GCTTTGCAGTGCGTGAGAAATGAGTTAAATTAA 16702

*********************************

Supplementary Figure 2: DNA sequence alignment of *cps* loci from serotype 33G (PMP1486, Genbank accession OR509570) and 33G-like (PMP1623, Genbank accession PQ281428) isolates of GPSC230. Highlighted positions denote differences.

M R K I R N I N L D L L K V L A C V G V

PMP1612 1 ATGAGAAAAATTCGAAATATCAACCTAGATTTACTAAAAGTGCTTGCATGTGTTGGAGTT 60

||||||||||||||||||||||||||||||||||||||||||||||||||||||||||||

PMP1615 1 ATGAGAAAAATTCGAAATATCAACCTAGATTTACTAAAAGTGCTTGCATGTGTTGGAGTT 60

M R K I R N I N L D L L K V L A C V G V

V L L H T T M G G F K E T G S Y N L L A

PMP1612 61 GTTTTACTTCATACAACAATGGGCGGATTTAAAGAGACAGGCTCATATAATCTTTTGGCA 120

||||||||||||||||||||||||||||||||||||||||||||||||||||||||||||

PMP1615 61 GTTTTACTTCATACAACAATGGGCGGATTTAAAGAGACAGGCTCATATAATCTTTTGGCA 120

V L L H T T M G G F K E T G S Y N L L A

Y L Y Y L G T Y S I P L F F M I N G Y L

PMP1612 121 TATTTATATTATTTAGGTACTTACTCTATTCCCCTGTTTTTTATGATCAATGGTTATTTA 180

||||||||||||||||||||||||||||||||||||||||||||||||||||||||||||

PMP1615 121 TATTTATATTATTTAGGTACTTACTCTATTCCCCTGTTTTTTATGATCAATGGTTATTTA 180

Y L Y Y L G T Y S I P L F F M I N G Y L

L L G K R E I T Y L Y I L Q K V K W I L

PMP1612 181 TTGTTAGGCAAGAGGGAAATAACTTATCTTTACATACTCCAGAAAGTAAAATGGATTTTA 240

||||||||||||||||||||||||||||||||||||||||||||||||||||||||||||

PMP1615 181 TTGTTAGGCAAGAGGGAAATAACTTATCTTTACATACTCCAGAAAGTAAAATGGATTTTA 240

L L G K R E I T Y L Y I L Q K V K W I L

I T V S S W T F I V W L F Y R D F T T N

PMP1612 241 ATAACAGTGTCATCATGGACATTTATCGTATGGCTTTTTTATCGTGATTTTACAACTAAT 300

||||||||||||||||||||||||||||||||||||||||||||||||||||||||||||

PMP1615 241 ATAACAGTGTCATCATGGACATTTATCGTATGGCTTTTTTATCGTGATTTTACAACTAAT 300

I T V S S W T F I V W L F Y R D F T T N

P I K K I V G S L I Q R G Y F S Q F W F

PMP1612 301 CCTATTAAAAAAATTGTAGGTTCTTTGATACAAAGAGGTTATTTCTCTCAGTTTTGGTTT 360

|||||||||||| |||||||||||||||||||||||||||||||||||||||||||||||

PMP1615 301 CCTATTAAAAAA-TTGTAGGTTCTTTGATACAAAGAGGTTATTTCTCTCAGTTTTGGTTT 359

P I K K L *

F G A L I L I Y L C L P I V R Q F L N S

PMP1612 361 TTCGGTGCACTAATACTTATCTATTTATGTTTGCCAATTGTGAGACAATTTCTAAATTCA 420

||||||||||||||||||||||||||||||||||||||||||||||||||||||||||||

PMP1615 360 TTCGGTGCACTAATACTTATCTATTTATGTTTGCCAATTGTGAGACAATTTCTAAATTCA 419

K R S Y L Y S L S L L M T I G L I F E L

PMP1612 421 AAAAGAAGCTATTTATACAGTTTATCTTTATTGATGACTATTGGTTTGATTTTTGAGTTA 480

||||||||||||||||||||||||||||||||||||||||||||||||||||||||||||

PMP1615 420 AAAAGAAGCTATTTATACAGTTTATCTTTATTGATGACTATTGGTTTGATTTTTGAGTTA 479

L N I L L Q M P I Q T Y V I Q T F R L W

PMP1612 481 TTAAATATCCTACTTCAGATGCCAATACAAACATATGTAATACAGACTTTTAGATTATGG 540

||||||||||||||||||||||||||||||||||||||||||||||||||||||||||||

PMP1615 480 TTAAATATCCTACTTCAGATGCCAATACAAACATATGTAATACAGACTTTTAGATTATGG 539

T W F F Y Y L L G G Y I A Q F T K E E I

PMP1612 541 ACGTGGTTTTTTTACTATCTTTTAGGTGGTTATATAGCGCAATTCACTAAAGAAGAAATC 600

||||||||||||||||||||||||||||||||||||||||||||||||||||||||||||

PMP1615 540 ACGTGGTTTTTTTACTATCTTTTAGGTGGTTATATAGCGCAATTCACTAAAGAAGAAATC 599

E S R F K N W M K I A S I L L L L I S P

PMP1612 601 GAATCAAGATTTAAGAATTGGATGAAAATAGCTAGCATACTTTTGTTATTGATTTCACCA 660

||||||||||||||||||||||||||||||||||||||||||||||||||||||||||||

PMP1615 600 GAATCAAGATTTAAGAATTGGATGAAAATAGCTAGCATACTTTTGTTATTGATTTCACCA 659

I I L F F I A K T T Y H N F F A E Y F Y

PMP1612 661 ATAATATTATTTTTCATAGCAAAGACCACTTACCATAATTTTTTTGCTGAATATTTTTAT 720

||||||||||||||||||||||||||||||||||||||||||||||||||||||||||||

PMP1615 660 ATAATATTATTTTTCATAGCAAAGACCACTTACCATAATTTTTTTGCTGAATATTTTTAT 719

D I L L V K V V S V G I F L T I F S L V

PMP1612 721 GATATTTTATTAGTAAAAGTGGTAAGCGTAGGGATTTTTCTAACTATTTTCTCACTTGTA 780

||||||||||||||||||||||||||||||||||||||||||||||||||||||||||||

PMP1615 720 GATATTTTATTAGTAAAAGTGGTAAGCGTAGGGATTTTTCTAACTATTTTCTCACTTGTA 779

L N E N S N K W I I F L S N Q T M G I F

PMP1612 781 TTGAATGAAAATAGCAACAAATGGATTATTTTTCTTTCTAATCAAACTATGGGTATCTTT 840

||||||||||||||||||||||||||||||||||||||||||||||||||||||||||||

PMP1615 780 TTGAATGAAAATAGCAACAAATGGATTATTTTTCTTTCTAATCAAACTATGGGTATCTTT 839

I I H T Y I M K V W E K L F G F S F I G

PMP1612 841 ATAATACACACTTATATTATGAAGGTATGGGAAAAACTATTTGGTTTTAGTTTTATAGGT 900

||||||||||||||||||||||||||||||||||||||||||||||||||||||||||||

PMP1615 840 ATAATACACACTTATATTATGAAGGTATGGGAAAAACTATTTGGTTTTAGTTTTATAGGT 899

S Y L L F A I F T L S V S F I I V G M L

PMP1612 901 TCATATTTACTTTTTGCTATATTTACTTTAAGTGTTAGTTTTATCATTGTTGGAATGTTA 960

||||||||||||||||||||||||||||||||||||||||||||||||||||||||||||

PMP1615 900 TCATATTTACTTTTTGCTATATTTACTTTAAGTGTTAGTTTTATCATTGTTGGAATGTTA 959

M K I P Y F N R I V K L *

PMP1612 961 ATGAAAATTCCGTATTTTAATCGAATCGTCAAATTATAA 999

|||||||||||||||||||||||||||||||||||||||

PMP1615 960 ATGAAAATTCCGTATTTTAATCGAATCGTCAAATTATAA 998

Supplementary Figure 3: Open reading frames of *wciG* in PMP1612 (33G) and PMP1615 (33G-like). Differences and codon changes are highlighted. Alignment was generated using SerialCloner 2.6.1.

M R K I R N I N L D L L K V L A C V G V

PMP1486 1 ATGAGAAAAATTCGAAATATCAACCTAGATTTACTAAAAGTGCTTGCATGTGTTGGAGTT 60

||||||||||||||||||||||||||||||||||||||||||||||||||||||||||||

PMP1623 1 ATGAGAAAAATTCGAAATATCAACCTAGATTTACTAAAAGTGCTTGCATGTGTTGGAGTT 60

M R K I R N I N L D L L K V L A C V G V

V L L H T T M G G F K E T G S Y N L L A

PMP1486 61 GTTTTACTTCATACAACAATGGGCGGATTTAAAGAGACAGGCTCATATAATCTTTTGGCA 120

||||||||||||||||||||||||||||||||||||||||||||||||||||||||||||

PMP1623 61 GTTTTACTTCATACAACAATGGGCGGATTTAAAGAGACAGGCTCATATAATCTTTTGGCA 120

V L L H T T M G G F K E T G S Y N L L A

Y L Y Y L G T Y S I P L F F M I N G Y L

PMP1486 121 TATTTATATTATTTAGGTACTTACTCTATTCCCCTGTTTTTT-ATGATCAATGGTTATTT 179

|||||||||||||||||||||||||||||||||||||||||| |||||||||||||||||

PMP1623 121 TATTTATATTATTTAGGTACTTACTCTATTCCCCTGTTTTTTTATGATCAATGGTTATTT 180

Y L Y Y L G T Y S I P L F F Y D Q W L F

L L G K R E I T Y L Y I L Q K V K W I L

PMP1486 180 ATTGTTAGGCAAGAGGGAAATAACTTATCTTTACATACTCCAGAAAGTAAAATGGATTTT 239

||||||||||||||||||||||||||||||||||||||||||||||||||||||||||||

PMP1623 181 ATTGTTAGGCAAGAGGGAAATAACTTATCTTTACATACTCCAGAAAGTAAAATGGATTTT 240

I V R Q E G N N L S L H T P E S K M D F

I T V S S W T F I V W L F Y R D F T T N

PMP1486 240 AATAACAGTGTCATCATGGACATTTATCGTATGGCTTTTTTATCGTGATTTTACAACTAA 299

||||||||||||||||||||||||||||||||||||||||||||||||||||||||||||

PMP1623 241 AATAACAGTGTCATCATGGACATTTATCGTATGGCTTTTTTATCGTGATTTTACAACTAA 300

N N S V I M D I Y R M A F L S *

P I K K I V G S L I Q R G Y F S Q F W F

PMP1486 300 TCCTATTAAAAAAATTGTAGGTTCTTTGATACAAAGAGGTTATTTCTCTCAGTTTTGGTT 359

||||||||||||||||||||||||||||||||||||||||||||||||||||||||||||

PMP1623 301 TCCTATTAAAAAAATTGTAGGTTCTTTGATACAAAGAGGTTATTTCTCTCAGTTTTGGTT 360

F G A L I L I Y L C L P I V R Q F L N S

PMP1486 360 TTTCGGTGCACTAATACTTATCTATTTATGTTTGCCAATTGTGAGACAATTTCTAAATTC 419

||||||||||||||||||||||||||||||||||||||||||||||||||||||||||||

PMP1623 361 TTTCGGTGCACTAATACTTATCTATTTATGTTTGCCAATTGTGAGACAATTTCTAAATTC 420

K R S Y L Y S L S L L M T I G L I F E L

PMP1486 420 AAAAAGAAGCTATTTATACAGTTTATCTTTATTGATGACTATTGGTTTGATTTTTGAGTT 479

||||||||||||||||||||||||||||||||||||||||||||||||||||||||||||

PMP1623 421 AAAAAGAAGCTATTTATACAGTTTATCTTTATTGATGACTATTGGTTTGATTTTTGAGTT 480

L N I L L Q M P I Q T Y V I Q T F R L W

PMP1486 480 ATTAAATATCCTACTTCAGATGCCAATACAAACATATGTAATACAGACTTTTAGATTATG 539

||||||||||||||||||||||||||||||||||||||||||||||||||||||||||||

PMP1623 481 ATTAAATATCCTACTTCAGATGCCAATACAAACATATGTAATACAGACTTTTAGATTATG 540

T W F F Y Y L L G G Y I A Q F T K E E I

PMP1486 540 GACGTGGTTTTTTTACTATCTTTTAGGTGGTTATATAGCGCAATTCACTAAAGAAGAAAT 599

||||||||||||||||||||||||||||||||||||||||||||||||||||||||||||

PMP1623 541 GACGTGGTTTTTTTACTATCTTTTAGGTGGTTATATAGCGCAATTCACTAAAGAAGAAAT 600

E S R F K N W M K I A S I L L L L I S P

PMP1486 600 CGAATCAAGATTTAAGAATTGGATGAAAATAGCTAGCATACTTTTGTTATTGATTTCACC 659

||||||||||||||||||||||||||||||||||||||||||||||||||||||||||||

PMP1623 601 CGAATCAAGATTTAAGAATTGGATGAAAATAGCTAGCATACTTTTGTTATTGATTTCACC 660

I I L F F I A K T T Y H N F F A E Y F Y

PMP1486 660 AATAATATTATTTTTCATAGCAAAGACCACTTACCATAATTTTTTTGCTGAATATTTTTA 719

||||||||||||||||||||||||||||||||||||||||||||||||||||||||||||

PMP1623 661 AATAATATTATTTTTCATAGCAAAGACCACTTACCATAATTTTTTTGCTGAATATTTTTA 720

D I L L V K V V S V G I F L T I F S L V

PMP1486 720 TGATATTTTATTAGTAAAAGTGGTAAGCGTAGGGATTTTTCTAACTATTTTCTCACTTGT 779

|||||||||||||||||||||||||||||||||||||||||||||| |||

PMP1623 721 TGATATTTTATTAGTAAAAGTGGTAAGCGTAGGGATTTTTCTAACT-----------TGT 769

L N E N S N K W I I F L S N Q T M G I F

PMP1486 780 ATTGAATGAAAATAGCAACAAATGGATTATTTTTCTTTCTAATCAAACTATGGGTATCTT 839

||||||||||||||||||||||||||||||||||||||||||||||||||||||||||||

PMP1623 770 ATTGAATGAAAATAGCAACAAATGGATTATTTTTCTTTCTAATCAAACTATGGGTATCTT 829

I I H T Y I M K V W E K L F G F S F I G

PMP1486 840 TATAATACACACTTATATTATGAAGGTATGGGAAAAACTATTTGGTTTTAGTTTTATAGG 899

||||||||||||||||||||||||||||||||||||||||||||||||||||||||||||

PMP1623 830 TATAATACACACTTATATTATGAAGGTATGGGAAAAACTATTTGGTTTTAGTTTTATAGG 889

S Y L L F A I F T L S V S F I I V G M L

PMP1486 900 TTCATATTTACTTTTTGCTATATTTACTTTAAGTGTTAGTTTTATCATTGTTGGAATGTT 959

||||||||||||||||||||||||||||||||||||||||||||||||||||||||||||

PMP1623 890 TTCATATTTACTTTTTGCTATATTTACTTTAAGTGTTAGTTTTATCATTGTTGGAATGTT 949

M K I P Y F N R I V K L *

PMP1486 960 AATGAAAATTCCGTATTTTAATCGAATCGTCAAATTATAA 999

||||||||||||||||||||||||||||||||||||||||

PMP1623 950 AATGAAAATTCCGTATTTTAATCGAATCGTCAAATTATAA 989

Supplementary Figure 4: Open reading frames of *wciG* in PMP1486 (33G) and PMP1623 (33G-like). Differences and codon changes are highlighted. Alignment was generated using SerialCloner 2.6.1.

PMP1623_wciG TTGCAGCGCTTGGTGGAATCATTTATCTTTTTGCAATTCTATCTCTGAAAGTGGTAGATG 60

33G_wciG ------------------------------------------------------------ 0

PMP1615_wciG -TTCAGCGCTTGGTGGAATCATTTATCTTTTTGCAATTCTATCTCTGAAAGTGGTAGATG 59

PMP1623_wciG TGAAAGAATTAAAACAAATTATTAGGAAAAACTAAAATGAGAAAAATTCGAAATATCAAC 120

33G_wciG ------------------------------------ATGAGAAAAATTCGAAATATCAAC 24

PMP1615_wciG TGAAAGAATTAAAACAAATTATTAGGAAAAACTAAAATGAGAAAAATTCGAAATATCAAC 119

************************

PMP1623_wciG CTAGATTTACTAAAAGTGCTTGCATGTGTTGGAGTTGTTTTACTTCATACAACAATGGGC 180

33G_wciG CTAGATTTACTAAAAGTGCTTGCATGTGTTGGAGTTGTTTTACTTCATACAACAATGGGC 84

PMP1615_wciG CTAGATTTACTAAAAGTGCTTGCATGTGTTGGAGTTGTTTTACTTCATACAACAATGGGC 179

************************************************************

PMP1623_wciG GGATTTAAAGAGACAGGCTCATATAATCTTTTGGCATATTTATATTATTTAGGTACTTAC 240

33G_wciG GGATTTAAAGAGACAGGCTCATATAATCTTTTGGCATATTTATATTATTTAGGTACTTAC 144

PMP1615_wciG GGATTTAAAGAGACAGGCTCATATAATCTTTTGGCATATTTATATTATTTAGGTACTTAC 239

************************************************************

PMP1623_wciG TCTATTCCCCTGTTTTTTTATGATCAATGGTTATTTATTGTTAGGCAAGAGGGAAATAAC 300

33G_wciG TCTATTCCCCTGTTT-TTTATGATCAATGGTTATTTATTGTTAGGCAAGAGGGAAATAAC 203

PMP1615_wciG TCTATTCCCCTGTTT-TTTATGATCAATGGTTATTTATTGTTAGGCAAGAGGGAAATAAC 298

*************** ********************************************

PMP1623_wciG TTATCTTTACATACTCCAGAAAGTAAAATGGATTTTAATAACAGTGTCATCATGGACATT 360

33G_wciG TTATCTTTACATACTCCAGAAAGTAAAATGGATTTTAATAACAGTGTCATCATGGACATT 263

PMP1615_wciG TTATCTTTACATACTCCAGAAAGTAAAATGGATTTTAATAACAGTGTCATCATGGACATT 358

************************************************************

PMP1623_wciG TATCGTATGGCTTTTTTATCGTGATTTTACAACTAATCCTATTAAAAAAATTGTAGGTTC 420

33G_wciG TATCGTATGGCTTTTTTATCGTGATTTTACAACTAATCCTATTAAAAAAATTGTAGGTTC 323

PMP1615_wciG TATCGTATGGCTTTTTTATCGTGATTTTACAACTAATCCTATTAAA-AAATTGTAGGTTC 417

********************************************** *************

PMP1623_wciG TTTGATACAAAGAGGTTATTTCTCTCAGTTTTGGTTTTTCGGTGCACTAATACTTATCTA 480

33G_wciG TTTGATACAAAGAGGTTATTTCTCTCAGTTTTGGTTTTTCGGTGCACTAATACTTATCTA 383

PMP1615_wciG TTTGATACAAAGAGGTTATTTCTCTCAGTTTTGGTTTTTCGGTGCACTAATACTTATCTA 477

************************************************************

PMP1623_wciG TTTATGTTTGCCAATTGTGAGACAATTTCTAAATTCAAAAAGAAGCTATTTATACAGTTT 540

33G_wciG TTTATGTTTGCCAATTGTGAGACAATTTCTAAATTCAAAAAGAAGCTATTTATACAGTTT 443

PMP1615_wciG TTTATGTTTGCCAATTGTGAGACAATTTCTAAATTCAAAAAGAAGCTATTTATACAGTTT 537

************************************************************

PMP1623_wciG ATCTTTATTGATGACTATTGGTTTGATTTTTGAGTTATTAAATATCCTACTTCAGATGCC 600

33G_wciG ATCTTTATTGATGACTATTGGTTTGATTTTTGAGTTATTAAATATCCTACTTCAGATGCC 503

PMP1615_wciG ATCTTTATTGATGACTATTGGTTTGATTTTTGAGTTATTAAATATCCTACTTCAGATGCC 597

************************************************************

PMP1623_wciG AATACAAACATATGTAATACAGACTTTTAGATTATGGACGTGGTTTTTTTACTATCTTTT 660

33G_wciG AATACAAACATATGTAATACAGACTTTTAGATTATGGACGTGGTTTTTTTACTATCTTTT 563

PMP1615_wciG AATACAAACATATGTAATACAGACTTTTAGATTATGGACGTGGTTTTTTTACTATCTTTT 657

************************************************************

PMP1623_wciG AGGTGGTTATATAGCGCAATTCACTAAAGAAGAAATCGAATCAAGATTTAAGAATTGGAT 720

33G_wciG AGGTGGTTATATAGCGCAATTCACTAAAGAAGAAATCGAATCAAGATTTAAGAATTGGAT 623

PMP1615_wciG AGGTGGTTATATAGCGCAATTCACTAAAGAAGAAATCGAATCAAGATTTAAGAATTGGAT 717

************************************************************

PMP1623_wciG GAAAATAGCTAGCATACTTTTGTTATTGATTTCACCAATAATATTATTTTTCATAGCAAA 780

33G_wciG GAAAATAGCTAGCATACTTTTGTTATTGATTTCACCAATAATATTATTTTTCATAGCAAA 683

PMP1615_wciG GAAAATAGCTAGCATACTTTTGTTATTGATTTCACCAATAATATTATTTTTCATAGCAAA 777

************************************************************

PMP1623_wciG GACCACTTACCATAATTTTTTTGCTGAATATTTTTATGATATTTTATTAGTAAAAGTGGT 840

33G_wciG GACCACTTACCATAATTTTTTTGCTGAATATTTTTATGATATTTTATTAGTAAAAGTGGT 743

PMP1615_wciG GACCACTTACCATAATTTTTTTGCTGAATATTTTTATGATATTTTATTAGTAAAAGTGGT 837

************************************************************

PMP1623_wciG AAGCGTAGGGATTTTTCTA-----------ACTTGTATTGAATGAAAATAGCAACAAATG 889

33G_wciG AAGCGTAGGGATTTTTCTAACTATTTTCTCACTTGTATTGAATGAAAATAGCAACAAATG 803

PMP1615_wciG AAGCGTAGGGATTTTTCTAACTATTTTCTCACTTGTATTGAATGAAAATAGCAACAAATG 897

******************* ******************************

PMP1623_wciG GATTATTTTTCTTTCTAATCAAACTATGGGTATCTTTATAATACACACTTATATTATGAA 949

33G_wciG GATTATTTTTCTTTCTAATCAAACTATGGGTATCTTTATAATACACACTTATATTATGAA 863

PMP1615_wciG GATTATTTTTCTTTCTAATCAAACTATGGGTATCTTTATAATACACACTTATATTATGAA 957

************************************************************

PMP1623_wciG GGTATGGGAAAAACTATTTGGTTTTAGTTTTATAGGTTCATATTTACTTTTTGCTATATT 1009

33G_wciG GGTATGGGAAAAACTATTTGGTTTTAGTTTTATAGGTTCATATTTACTTTTTGCTATATT 923

PMP1615_wciG GGTATGGGAAAAACTATTTGGTTTTAGTTTTATAGGTTCATATTTACTTTTTGCTATATT 1017

************************************************************

PMP1623_wciG TACTTTAAGTGTTAGTTTTATCATTGTTGGAATGTTAATGAAAATTCCGTATTTTAATCG 1069

33G_wciG TACTTTAAGTGTTAGTTTTATCATTGTTGGAATGTTAATGAAAATTCCGTATTTTAATCG 983

PMP1615_wciG TACTTTAAGTGTTAGTTTTATCATTGTTGGAATGTTAATGAAAATTCCGTATTTTAATCG 1077

************************************************************

PMP1623_wciG AATCGTCAAATTATAAAAAGGAGAAAAAATGTACGATTATCTTGTTGTTGTGCCTGGGTC 1129

33G_wciG AATCGTCAAATTATAA-------------------------------------------- 999

PMP1615_wciG AATCGTCAAATTATAAAAAGGAGAAAAAATGTACGATTATCTTGTTGTTGTGCC------ 1131

****************

PMP1623_wciG TCAA 1133

33G_wciG ---- 999

PMP1615_wciG ---- 1131

Supplementary Figure 5: DNA sequence alignment from Sanger sequencing assemblies of *wciG* PCR products amplified from DNA extracted from pneumococcal 33G-like isolates PMP1615 and PMP1623. Highlighted positions denote mutations compared with the reference intact *wciG* gene from the 33G *cps* locus.

Platesweep_PMP1623_wciG TTTCTAGCCCATTGCAGCGCTTGGTGGAATCATTTATCTTTTTGCAATTCTATCTCTGAA 60

33G_wciG ------------------------------------------------------------ 0

Platesweep_PMP1615_wciG ----------TTTTCAGCGCTTGGTGGAATCATTTATCTTTTTGCAATTCTATCTCTGAA 50

Platesweep_PMP1623_wciG AGTGGTAGATGTGAAAGAATTAAAACAAATTATTAGGAAAAACTAAAATGAGAAAAATTC 120

33G_wciG -----------------------------------------------ATGAGAAAAATTC 13

Platesweep_PMP1615_wciG AGTGGTAGATGTGAAAGAATTAAAACAAATTATTAGGAAAAACTAAAATGAGAAAAATTC 110

*************

Platesweep_PMP1623_wciG GAAATATCAACCTAGATTTACTAAAAGTGCTTGCATGTGTTGGAGTTGTTTTACTTCATA 180

33G_wciG GAAATATCAACCTAGATTTACTAAAAGTGCTTGCATGTGTTGGAGTTGTTTTACTTCATA 73

Platesweep_PMP1615_wciG GAAATATCAACCTAGATTTACTAAAAGTGCTTGCATGTGTTGGAGTTGTTTTACTTCATA 170

************************************************************

Platesweep_PMP1623_wciG CAACAATGGGCGGATTTAAAGAGACAGGCTCATATAATCTTTTGGCATATTTATATTATT 240

33G_wciG CAACAATGGGCGGATTTAAAGAGACAGGCTCATATAATCTTTTGGCATATTTATATTATT 133

Platesweep_PMP1615_wciG CAACAATGGGCGGATTTAAAGAGACAGGCTCATATAATCTTTTGGCATATTTATATTATT 230

************************************************************

Platesweep_PMP1623_wciG TAGGTACTTACTCTATTCCCCTGTTTTTTTATGATCAATGGTTATTTATTGTTAGGCAAG 300

33G_wciG TAGGTACTTACTCTATTCCCCTGTTT-TTTATGATCAATGGTTATTTATTGTTAGGCAAG 192

Platesweep_PMP1615_wciG TAGGTACTTACTCTATTCCCCTGTTT-TTTATGATCAATGGTTATTTATTGTTAGGCAAG 289

************************** *********************************

Platesweep_PMP1623_wciG AGGGAAATAACTTATCTTTACATACTCCAGAAAGTAAAATGGATTTTAATAACAGTGTCA 360

33G_wciG AGGGAAATAACTTATCTTTACATACTCCAGAAAGTAAAATGGATTTTAATAACAGTGTCA 252

Platesweep_PMP1615_wciG AGGGAAATAACTTATCTTTACATACTCCAGAAAGTAAAATGGATTTTAATAACAGTGTCA 349

************************************************************

Platesweep_PMP1623_wciG TCATGGACATTTATCGTATGGCTTTTTTATCGTGATTTTACAACTAATCCTATTAAAAAA 420

33G_wciG TCATGGACATTTATCGTATGGCTTTTTTATCGTGATTTTACAACTAATCCTATTAAAAAA 312

Platesweep_PMP1615_wciG TCATGGACATTTATCGTATGGCTTTTTTATCGTGATTTTACAACTAATCCTATTAAA-AA 408

********************************************************* **

Platesweep_PMP1623_wciG ATTGTAGGTTCTTTGATACAAAGAGGTTATTTCTCTCAGTTTTGGTTTTTCGGTGCACTA 480

33G_wciG ATTGTAGGTTCTTTGATACAAAGAGGTTATTTCTCTCAGTTTTGGTTTTTCGGTGCACTA 372

Platesweep_PMP1615_wciG ATTGTAGGTTCTTTGATACAAAGAGGTTATTTCTCTCAGTTTTGGTTTTTCGGTGCACTA 468

************************************************************

Platesweep_PMP1623_wciG ATACTTATCTATTTATGTTTGCCAATTGTGAGACAATTTCTAAATTCAAAAAGAAGCTAT 540

33G_wciG ATACTTATCTATTTATGTTTGCCAATTGTGAGACAATTTCTAAATTCAAAAAGAAGCTAT 432

Platesweep_PMP1615_wciG ATACTTATCTATTTATGTTTGCCAATTGTGAGACAATTTCTAAATTCAAAAAGAAGCTAT 528

************************************************************

Platesweep_PMP1623_wciG TTATACAGTTTATCTTTATTGATGACTATTGGTTTGATTTTTGAGTTATTAAATATCCTA 600

33G_wciG TTATACAGTTTATCTTTATTGATGACTATTGGTTTGATTTTTGAGTTATTAAATATCCTA 492

Platesweep_PMP1615_wciG TTATACAGTTTATCTTTATTGATGACTATTGGTTTGATTTTTGAGTTATTAAATATCCTA 588

************************************************************

Platesweep_PMP1623_wciG CTTCAGATGCCAATACAAACATATGTAATACAGACTTTTAGATTATGGACGTGGTTTTTT 660

33G_wciG CTTCAGATGCCAATACAAACATATGTAATACAGACTTTTAGATTATGGACGTGGTTTTTT 552

Platesweep_PMP1615_wciG CTTCAGATGCCAATACAAACATATGTAATACAGACTTTTAGATTATGGACGTGGTTTTTT 648

************************************************************

Platesweep_PMP1623_wciG TACTATCTTTTAGGTGGTTATATAGCGCAATTCACTAAAGAAGAAATCGAATCAAGATTT 720

33G_wciG TACTATCTTTTAGGTGGTTATATAGCGCAATTCACTAAAGAAGAAATCGAATCAAGATTT 612

Platesweep_PMP1615_wciG TACTATCTTTTAGGTGGTTATATAGCGCAATTCACTAAAGAAGAAATCGAATCAAGATTT 708

************************************************************

Platesweep_PMP1623_wciG AAGAATTGGATGAAAATAGCTAGCATACTTTTGTTATTGATTTCACCAATAATATTATTT 780

33G_wciG AAGAATTGGATGAAAATAGCTAGCATACTTTTGTTATTGATTTCACCAATAATATTATTT 672

Platesweep_PMP1615_wciG AAGAATTGGATGAAAATAGCTAGCATACTTTTGTTATTGATTTCACCAATAATATTATTT 768

************************************************************

Platesweep_PMP1623_wciG TTCATAGCAAAGACCACTTACCATAATTTTTTTGCTGAATATTTTTATGATATTTTATTA 840

33G_wciG TTCATAGCAAAGACCACTTACCATAATTTTTTTGCTGAATATTTTTATGATATTTTATTA 732

Platesweep_PMP1615_wciG TTCATAGCAAAGACCACTTACCATAATTTTTTTGCTGAATATTTTTATGATATTTTATTA 828

************************************************************

Platesweep_PMP1623_wciG GTAAAAGTGGTAAGCGTAGGGATTTTTCTA-----------ACTTGTATTGAATGAAAAT 889

33G_wciG GTAAAAGTGGTAAGCGTAGGGATTTTTCTAACTATTTTCTCACTTGTATTGAATGAAAAT 792

Platesweep_PMP1615_wciG GTAAAAGTGGTAAGCGTAGGGATTTTTCTAACTATTTTCTCACTTGTATTGAATGAAAAT 888

****************************** *******************

Platesweep_PMP1623_wciG AGCAACAAATGGATTATTTTTCTTTCTAATCAAACTATGGGTATCTTTATAATACACACT 949

33G_wciG AGCAACAAATGGATTATTTTTCTTTCTAATCAAACTATGGGTATCTTTATAATACACACT 852

Platesweep_PMP1615_wciG AGCAACAAATGGATTATTTTTCTTTCTAATCAAACTATGGGTATCTTTATAATACACACT 948

************************************************************

Platesweep_PMP1623_wciG TATATTATGAAGGTATGGGAAAAACTATTTGGTTTTAGTTTTATAGGTTCATATTTACTT 1009

33G_wciG TATATTATGAAGGTATGGGAAAAACTATTTGGTTTTAGTTTTATAGGTTCATATTTACTT 912

Platesweep_PMP1615_wciG TATATTATGAAGGTATGGGAAAAACTATTTGGTTTTAGTTTTATAGGTTCATATTTACTT 1008

************************************************************

Platesweep_PMP1623_wciG TTTGCTATATTTACTTTAAGTGTTAGTTTTATCATTGTTGGAATGTTAATGAAAATTCCG 1069

33G_wciG TTTGCTATATTTACTTTAAGTGTTAGTTTTATCATTGTTGGAATGTTAATGAAAATTCCG 972

Platesweep_PMP1615_wciG TTTGCTATATTTACTTTAAGTGTTAGTTTTATCATTGTTGGAATGTTAATGAAAATTCCG 1068

************************************************************

Platesweep_PMP1623_wciG TATTTTAATCGAATCGTCAAATTATAAAAAGGAGAAAAAATGTACGATTATCTTGTTGTT 1129

33G_wciG TATTTTAATCGAATCGTCAAATTATAA--------------------------------- 999

Platesweep_PMP1615_wciG TATTTTAATCGAATCGTCAAATTATAAAAAGGAGAAAAAATGTACGATTATCTTGTTGTT 1128

***************************

Platesweep_PMP1623_wciG GTGCCTGGGTCT 1141

33G_wciG ------------ 999

Platesweep_PMP1615_wciG GTGG-------- 1132

Supplementary Figure 6: DNA sequence alignment from Sanger sequencing assemblies of *wciG* PCR products amplified from DNA extracted from a sweep of bacterial growth cultured from the nasopharyngeal swab. Highlighted positions denote mutations compared with the reference intact *wciG* gene from the 33G *cps* locus.


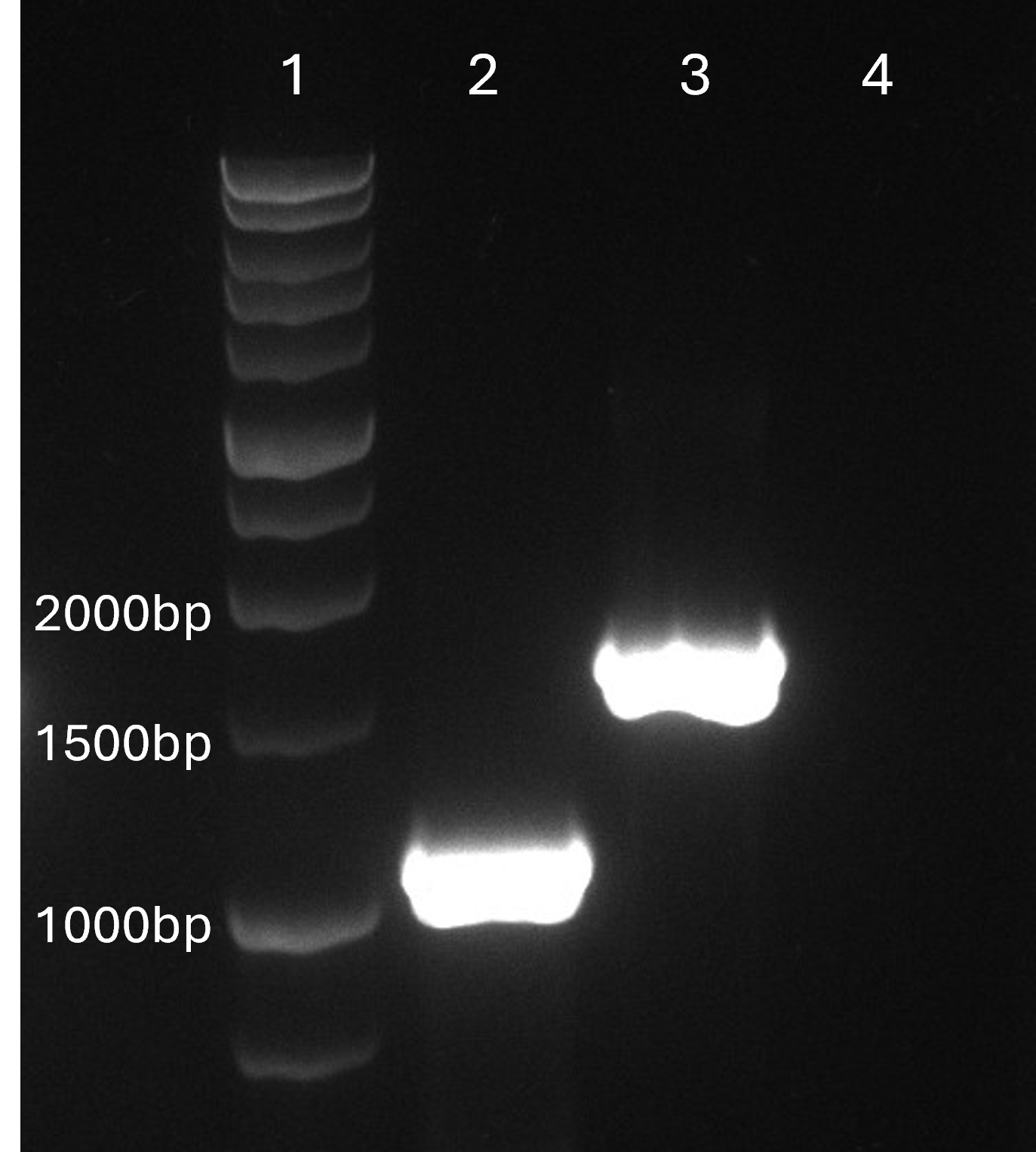


Supplementary Figure 7: PCR amplification of *wciG* region in 33G strain PMP1612 (lane 2) and Δ*wciG* mutant (lane 3) constructed in PMP1612 background where 798bp of the *wciG* gene was deleted and replaced with the Janus cassette (1390bp). Lane 1 is a 1 kb ladder (Promega). Lane 4 is a no template control. PCRs were performed as described in the methods using primers listed in Supplementary Table 1 that bind and up and downstream of the *wciG* gene. Expected amplicon sizes were 1197bp and 1789bp for PMP1612 and Δ*wciG,* respectively.

Janus ------------------------------------------------------------ 0

ΔwciG CTTTTTGCATTCTATCTCTGAAAGTGGTAGATGTGAAAGAATTAAAACAAATTATTAGGA 60

Janus --------------------------------------------------------CCGT 4

ΔwciG AAAACTAAAATGAGAAAAATTCGAAATATCAACCTAGATTTACTAAAAGTGCTTGCCCGT 120

****

Janus TTGATTTTTAATGGATAATGTGATATAATCTTTAAATACTGTAGAAAAGAGGAAGGAAAT 64

ΔwciG TTGATTTTTAATGGATAATGTGATATAATCTTTAAATACTGTAGAAAAGAGGAAGGAAAT 180

************************************************************

Janus AATAAATGGCTAAAATGAGAATATCACCGGAATTGAAAAAACTGATCGAAAAATACCGCT 124

ΔwciG AATAAATGGCTAAAATGAGAATATCACCGGAATTGAAAAAACTGATCGAAAAATACCGCT 240

************************************************************

Janus GCGTAAAAGATACGGAAGGAATGTCTCCTGCTAAGGTATATAAGCTGGTGGGAGAAAATG 184

ΔwciG GCGTAAAAGATACGGAAGGAATGTCTCCTGCTAAGGTATATAAGCTGGTGGGAGAAAATG 300

************************************************************

Janus AAAACCTATATTTAAAAATGACGGACAGCCGGTATAAAGGGACCACCTATGATGTGGAAC 244

ΔwciG AAAACCTATATTTAAAAATGACGGACAGCCGGTATAAAGGGACCACCTATGATGTGGAAC 360

************************************************************

Janus GGGAAAAGGACATGATGCTATGGCTGGAAGGAAAGCTGCCTGTTCCAAAGGTCCTGCACT 304

ΔwciG GGGAAAAGGACATGATGCTATGGCTGGAAGGAAAGCTGCCTGTTCCAAAGGTCCTGCACT 420

************************************************************

Janus TTGAACGGCATGATGGCTGGAGCAATCTGCTCATGAGTAAGGCCGATGGCGTCCTTTGCT 364

ΔwciG TTGAACGGCATGATGGCTGGAGCAATCTGCTCATGAGTAAGGCCGATGGCGTCCTTTGCT 480

************************************************************

Janus CGGAAGAGTATGAAGATGAACAAAGCCCTGAAAAGATTATCGAGCTGTATGCGGAGTGCA 424

ΔwciG CGGAAGAGTATGAAGATGAACAAAGCCCTGAAAAGATTATCGAGCTGTATGCGGAGTGCA 540

************************************************************

Janus TCAGGCTCTTTCACTCCATCGACATACCGGATTGTCCCTATACGAATAGCTTAGACAGCC 484

ΔwciG TCAGGCTCTTTCACTCCATCGACATACCGGATTGTCCCTATACGAATAGCTTAGACAGCC 600

************************************************************

Janus GCTTAGCCGAATTGGATTACTTACTGAATAACGATCTGGCCGATGTGGATTGCGAAAACT 544

ΔwciG GCTTAGCCGAATTGGATTACTTACTGAATAACGATCTGGCCGATGTGGATTGCGAAAACT 660

************************************************************

Janus GGGAAGAAGACACTCCATTTAAAGATCCGCGCGAGCTGTATGATTTTTTAAAGACGGAAA 604

ΔwciG GGGAAGAAGACACTCCATTTAAAGATCCGCGCGAGCTGTATGATTTTTTAAAGACGGAAA 720

************************************************************

Janus AGCCCGAAGAGGAACCTGTCTTTTCCCACGGCGACCTGGGAGACAGCAACATCTTTGTGA 664

ΔwciG AGCCCGAAGAGGAACCTGTCTTTTCCCACGGCGACCTGGGAGACAGCAACATCTTTGTGA 780

************************************************************

Janus AAGATGGCAAAGTAAGTGGCCTTATTGATCTTGGGAGAAGCGGCAGGGCGGACAAGTGGT 724

ΔwciG AAGATGGCAAAGTAAGTGGCCTTATTGATCTTGGGAGAAGCGGCAGGGCGGACAAGTGGT 840

************************************************************

Janus ATGACATTGCCTTCTGCGTCCGGTCGATCAGGGAGGATATCGGGGAAGAACAGTATGTCG 784

ΔwciG ATGACATTGCCTTCTGCGTCCGGTCGATCAGGGAGGATATCGGGGAAGAACAGTATGTCG 900

************************************************************

Janus AGCTATTTTTTGACTTACTGGGGATCAAGCCTGATTGGGAGAAAATAAAATATTATATTT 844

ΔwciG AGCTATTTTTTGACTTACTGGGGATCAAGCCTGATTGGGAGAAAATAAAATATTATATTT 960

************************************************************

Janus TACTGGATGAATTGTTTTAGTACCTAGAATTCACCAAAAATAAAAAAACACAGGAGAATG 904

ΔwciG TACTGGATGAATTGTTTTAGTACCTAGAATTCACCAAAAATAAAAAAACACAGGAGAATG 1020

************************************************************

Janus TAGATGCCTACAATTAACCAATTGGTTCGCAAACCGCGTAAATCAAAAGTAGAAAAATCT 964

ΔwciG TAGATGCCTACAATTAACCAATTGGTTCGCAAACCGCGTAAATCAAAAGTAGAAAAATCT 1080

************************************************************

Janus AAATCACCAGCTTTGAACGTTGGTTACAATAGTCATAAAAAAGTTCAAACAAACGTTTCT 1024

ΔwciG AAATCACCAGCTTTGAACGTTGGTTACAATAGTCATAAAAAAGTTCAAACAAACGTTTCT 1140

************************************************************

Janus TCACCGCAAAAACGTGGTGTCGCAACTCGTGTTGGAACAATGACACCTAAAAAACCTAAC 1084

ΔwciG TCACCGCAAAAACGTGGTGTCGCAACTCGTGTTGGAACAATGACACCTAAAAAACCTAAC 1200

************************************************************

Janus TCAGCCCTTCGTAAATTCGCTCGTGTACGTTTGAGCAACCTTATCGAAGTTACTGCCTAC 1144

ΔwciG TCAGCCCTTCGTAAATTCGCTCGTGTACGTTTGAGCAACCTTATCGAAGTTACTGCCTAC 1260

************************************************************

Janus ATCCCAGGTATCGGACACAACTTGCAAGAGCACAGCGTGGTGCTTCTTCGCGGTGGACGT 1204

ΔwciG ATCCCAGGTATCGGACACAACTTGCAAGAGCACAGCGTGGTGCTTCTTCGCGGTGGACGT 1320

************************************************************

Janus GTAAAAGACCTTCCAGGGGTACGTTACCATATCGTCCGTGGTGCACTTGATACTGCAGGT 1264

ΔwciG GTAAAAGACCTTCCAGGGGTACGTTACCATATCGTCCGTGGTGCACTTGATACTGCAGGT 1380

************************************************************

Janus GTTAACGATCGTAAACAAGGCCGTTCTAAATACGGTACTAAACGTCCAAAAGCATAAGGA 1324

ΔwciG GTTAACGATCGTAAACAAGGCCGTTCTAAATACGGTACTAAACGTCCAAAAGCATAAGGA 1440

************************************************************

Janus AAGGGGATAAAGAGAAA------------------------------------------- 1341

ΔwciG AAGGGGATAAAGAGAAAACACACTTATATTATGAAGGTATGGGAAAAACTATTTGGTTTT 1500

*****************

Janus ------------------------------------------------------------ 1341

ΔwciG AGTTTTATAGGTTCATATTTACTTTTTGCTATATTTACTTTAAGTGTTAGTTTTATCATT 1560

Janus ------------------------------------------------------------ 1341

ΔwciG GTTGGAATGTTAATGAAAATTCCGTATTTTAATCGAATCGTCAAATTATAAAAAGGGAAA 1620

Janus -- 1341

ΔwciG AA 1622

Supplementary Figure 8: DNA sequence alignment the Janus selectable marker cassette compared with the Sanger sequence of the PCR product of the *wciG* region in the Δ*wciG* mutant amplified using primers listed in Supplementary Table 1 that bind and up and downstream of the *wciG* gene. The Δ*wciG* mutant was constructed in a PMP1612 background where 798bp of the *wciG* gene (original length = 999bp) was deleted and replaced with the Janus cassette. Blue highlight is the remnant *wciG* sequence.

35B_wciG ATGCATAAATTTAAAGATATTAACTTGGATTTACTAAAAGTAATTGCATGTGTTGGAGTT 60

33G_wciG ATGAGAAAAATTCGAAATATCAACCTAGATTTACTAAAAGTGCTTGCATGTGTTGGAGTT 60

*** *** ** * **** *** * ************** *****************

35B_wciG GTTTTACTCCATACAGAGATGGGGGGATTTAAAGAGACAGGTTCATGGAATTTTTCGACA 120

33G_wciG GTTTTACTTCATACAACAATGGGCGGATTTAAAGAGACAGGCTCATATAATCTTTTGGCA 120

******** ****** ***** ***************** **** *** *** * **

35B_wciG TATTTATACTACTTAGGAACCTATTCTATCCCTCTATTTTTTATAGTTAATGGTTATTTA 180

33G_wciG TATTTATATTATTTAGGTACTTACTCTATTCCCCTGTTTTTTATGATCAATGGTTATTTA 180

******** ** ***** ** ** ***** ** ** ******** * ************

35B_wciG TTGTTAGGGAAGAAAGAGATTACCTATTCCTACATATTACAGAAAGTAAAATATATTCTA 240

33G_wciG TTGTTAGGCAAGAGGGAAATAACTTATCTTTACATACTCCAGAAAGTAAAATGGATTTTA 240

******** **** ** ** ** *** ****** * ************* *** **

35B_wciG ATAACAGTGCTGTCATGGACCTTTATAGTTTGGTTATTTAAACGGGATTTTTCAGTTAAT 300

33G_wciG ATAACAGTGTCATCATGGACATTTATCGTATGGCTTTTTTATCGTGATTTTACAACTAAT 300

********* ******** ***** ** *** * *** * ** ****** ** ****

35B_wciG CCAATAAAAAAACTTATAGGATCATTGGTGCAAAAAGGTTATTTCTTTCAGTTTTGGTTT 360

33G_wciG CCTATTAAAAAAATTGTAGGTTCTTTGATACAAAGAGGTTATTTCTCTCAGTTTTGGTTT 360

** ** ****** ** **** ** *** * **** *********** *************

35B_wciG TTTGGTGCGCTTATACTTATTTATATATGTCTCCCCGTTTTGAAACAATTTCTTAATTCA 420

33G_wciG TTCGGTGCACTAATACTTATCTATTTATGTTTGCCAATTGTGAGACAATTTCTAAATTCA 420

** ***** ** ******** *** ***** * ** ** *** ********* ******

35B_wciG AAAAGAAGTTATTTATACTTTTTATCTGTATTGCTAGTTATTGGTTTAATTTTTGAGTTA 480

33G_wciG AAAAGAAGCTATTTATACAGTTTATCTTTATTGATGACTATTGGTTTGATTTTTGAGTTA 480

******** ********* ******* ***** * ********* ************

35B_wciG ACAAATATTGTACTTCAAATGCCAATACAAACATATGTAATACAAACTTTTAGATTATGG 540

33G_wciG TTAAATATCCTACTTCAGATGCCAATACAAACATATGTAATACAGACTTTTAGATTATGG 540

****** ******* ************************** ***************

35B_wciG ACTTGGCTTTTCTATTATATTTTAGGGGGCTTTATATCTCAGTTTGATAAAAATACCATC 600

33G_wciG ACGTGGTTTTTTTACTATCTTTTAGGTGGTTATATAGCGCAATTCACTAAAGAAGAAATC 600

** *** **** ** *** ******* ** * **** * ** ** **** * ***

35B_wciG AAAAATGGGTTTAAGAGATGGATGAAAGTAATTGCAGTACTTTTATTATTAGTTTCGCCA 660

33G_wciG GAATCAAGATTTAAGAATTGGATGAAAATAGCTAGCATACTTTTGTTATTGATTTCACCA 660

** * ******* ********* ** * ******* ***** **** ***

35B_wciG TTTATATTATTTTTCATAGCAAAGACCACTTACCATAATTTTTTTGCTGAATATTTTTAT 720

33G_wciG ATAATATTATTTTTCATAGCAAAGACCACTTACCATAATTTTTTTGCTGAATATTTTTAT 720

* *********************************************************

35B_wciG GATATTTTATTAGTAAAAGTTGTAAGCGTAGGGATTTTTCTAACTATTTTCTCAATTGTA 780

33G_wciG GATATTTTATTAGTAAAAGTGGTAAGCGTAGGGATTTTTCTAACTATTTTCTCACTTGTA 780

******************** ********************************* *****

35B_wciG TTGAATGAAAATAGCAACAAATGGATTATTTTTCTTTCTAATCAAACTATGGGTATCTTT 840

33G_wciG TTGAATGAAAATAGCAACAAATGGATTATTTTTCTTTCTAATCAAACTATGGGTATCTTT 840

************************************************************

35B_wciG ATAATACACACTTATATTATGAAGGTATGGGAAAAACTATTTGGTTTTAATTTTATAGGT 900

33G_wciG ATAATACACACTTATATTATGAAGGTATGGGAAAAACTATTTGGTTTTAGTTTTATAGGT 900

************************************************* **********

35B_wciG TCATATTTACTTTTTGCTATATTTACTTTAAGTGTTAGTTTTATCATTGTTGGAATGTTA 960

33G_wciG TCATATTTACTTTTTGCTATATTTACTTTAAGTGTTAGTTTTATCATTGTTGGAATGTTA 960

************************************************************

35B_wciG ATGAAAATTCCGTATTTTAATCGAATCGTCAAATTATAA 999

33G_wciG ATGAAAATTCCGTATTTTAATCGAATCGTCAAATTATAA 999

***************************************

Supplementary Figure 9: DNA sequence alignment of *wciG* gene from representative strains of serotypes 35B (strain 4356/39, Genbank accession CR931705 (2)) and 33G (strain PMP1612, Genbank accession OR50958 (3)). Highlighted sequences denote homopolymeric regions where frameshift mutations have been reported previously in 35D isolates (4) and in 33H isolates from the current study.

**References**:

1. Sung, C. K., Li, H., Claverys, J. P., and Morrison, D. A. (2001) An *rpsL* Cassette, Janus, for Gene Replacement through Negative Selection in *Streptococcus* *pneumoniae*. *Appl. Environ. Microbiol.* **67**, 5190–5196

2. Bentley, S. D., Aanensen, D. M., Mavroidi, A., Saunders, D., Rabbinowitsch, E., Collins, M., Donohoe, K., Harris, D., Murphy, L., Quail, M. A., Samuel, G., Skovsted, I. C., Kaltoft, M. S., Barrell, B., Reeves, P. R., Parkhill, J., and Spratt, B. G. (2006) Genetic analysis of the capsular biosynthetic locus from all 90 pneumococcal serotypes. *PLoS Genet.* **2**, 0262–0269

3. Manna, S., Werren, J. P., Ortika, B. D., Bellich, B., Pell, C. L., Nikolaou, E., Gjuroski, I., Lo, S., Hinds, J., Tundev, O., Dunne, E. M., Gessner, B. D., Bentley, S. D., Russell, F. M., Mulholland, E. K., Mungun, T., von Mollendorf, C., Licciardi, P. V., Cescutti, P., Ravenscroft, N., Hilty, M., and Satzke, C. (2024) *Streptococcus* *pneumoniae* serotype 33G: genetic, serological, and structural analysis of a new capsule type. *Microbiol. Spectr.* 10.1128/spectrum.03579-23

4. Lo, S. W., Gladstone, R. A., Van Tonder, A. J., Hawkins, P. A., Kwambana-Adams, B., Cornick, J. E., Madhi, S. A., Nzenze, S. A., Du Plessis, M., Kandasamy, R., Carter, P. E., Eser, Ö. K., Ho, P. L., Elmdaghri, N., Shakoor, S., Clarke, S. C., Antonio, M., Everett, D. B., Von Gottberg, A., Klugman, K. P., McGee, L., Breiman, R. F., and Bentley, S. D. (2018) Global Distribution of Invasive Serotype 35D *Streptococcus* *pneumoniae* Isolates following Introduction of 13-Valent Pneumococcal Conjugate Vaccine. *J. Clin. Microbiol.* 10.1128/JCM.00228-18
